# The ADHD deficit in school performance across sex and parental education: A prospective sibling‐comparison register study of 344,152 Norwegian adolescents

**DOI:** 10.1002/jcv2.12064

**Published:** 2022-02-12

**Authors:** Hans Fredrik Sunde, Thomas H. Kleppestø, Kristin Gustavson, Magnus Nordmo, Bjørn‐Atle Reme, Fartein Ask Torvik

**Affiliations:** ^1^ Centre for Fertility and Health Norwegian Institute of Public Health Oslo Norway; ^2^ Department of Psychology University of Oslo Oslo Norway; ^3^ Department of Mental Disorders Norwegian Institute of Public Health Oslo Norway; ^4^ Promenta Research Centre, Department of Psychology, University of Oslo Oslo Norway

**Keywords:** ADHD, register data, school performance, sex differences, socioeconomic status

## Abstract

**Background:**

Attention‐Deficit Hyperactivity Disorder (ADHD) is associated with impaired school performance, but the impact of ADHD may vary across sex, family background, and school subjects. By using prospective population‐wide register data, we describe impairment in academic performance related to ADHD across different school subjects and investigate how this impairment differ across sex and parental education.

**Methods:**

We examined grades and Grade Point Averages (GPA) at age ∼16 among 344,152 Norwegian children born between 1997 and 2002. We linked grades with diagnoses from publicly funded general practitioners and with demographic information. Associations between ADHD diagnosed between age 10 and 16 and school performance were estimated with linear models, including sibling‐models which control for unobserved variables shared within families.

**Results:**

Children with ADHD (4.0%) had −1.11 standard deviations lower GPAs compared to children without ADHD. This difference remained substantial after adjusting for demographic factors (−0.87), comorbid mental disorders (−0.82), early school performance (−0.54), and when comparing full siblings (−0.60). The relative ADHD deficit was 22% larger for girls than for boys and 39% larger for children with highly educated parents than for children of parents without completed high school, but the absolute deficit was smaller.

**Conclusion:**

The ADHD deficit in school performance was large and not easily attributable to other factors. Because the ADHD deficit was large in all school subjects, interventions should ideally address factors that affect school performance broadly, although targeting theoretical subjects specifically may be most effective given limited resources.

## Graphical abstract

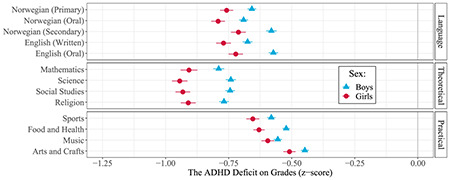

Children with ADHD have substantially lower grades in all school subjects compared to children without ADHD. The deficit is larger for girls and for children of highly educated parents. This was found when investigating register data on school performance at age 16 linked with health data from primary care use.

**Key points**

- Children with ADHD have substantially poorer school performance than unaffected children. The ADHD deficit is not easily attributable to other factors, which suggest that interventions must target ADHD symptoms directly independent of sex, parental education, early school performance, and other psychiatric disorders
- The relative ADHD deficit is larger for girls and for children of highly educated parents, but the absolute deficit is smaller (i.e., girls with ADHD still outperform boys with ADHD, and children with ADHD and highly educated parents still outperform children with ADHD and less educated parents with ADHD)
- The ADHD deficit is large in all school subjects, meaning interventions should target factors that is shared across different school subject, although the potential for improvement appears to be largest in theoretical subjects where the ADHD deficit is largest.

## INTRODUCTION

Attention Deficit‐Hyperactivity Disorder (ADHD) is a heritable neurodevelopmental disorder characterized by dysfunctional inattention, impulsivity, and hyperactivity (APA, 2013). ADHD has substantial socioeconomic costs, both for individuals and society (Faraone et al., 2021). Reduced school performance may be partially responsible for this: ADHD is associated with impaired school performance (Arnold et al., 2020; Daley & Birchwood, 2010; Kent et al., 2011; Polderman et al., 2010; Sayal et al., 2015), which again is an important determinant for adult education, employment, and income (Jangmo et al., 2021; Markussen et al., 2020).

Attention‐Deficit Hyperactivity Disorder is substantially more prevalent among boys than girls and among children of parents with less education or less income (Kinge et al., 2021; Larsson et al., 2014; Russell et al., 2016; Torvik et al., 2020). School performance also varies with sex and social background, like ADHD does. Girls generally outperform boys (OECD, 2015; Voyer & Voyer, 2014), and children with highly educated parents or from high income households generally outperform less advantaged children (OECD, 2016; Sirin, 2005; Statistics Norway, 2020). Different school performance in groups with different prevalence of ADHD raises the question of how the impact of ADHD may differ across groups. To the best of our knowledge, no study has investigated how the impact of ADHD varies by social background, and the studies on sex differences have been small and underpowered (e.g., DuPaul et al., 2006; Gershon, 2002). Furthermore, although ADHD has been linked with both reading disabilities and arithmetic difficulties (Taanila et al., 2012), little is known about to what extent ADHD leads to general deficits in school performance versus deficits specific to some school subjects. By investigating relative performance in different school subjects, we can point out where children are most affected by ADHD and hence where to target potential interventions.

The association between ADHD and school performance could be confounded by several factors that influence both (Polderman et al., 2010). First, family characteristics such as parental education are robustly associated with both school performance and ADHD (Kinge et al., 2021; Sirin, 2005). We account for this by comparing full siblings, which controls for unobserved variables shared within families, such as social, geographic, and parental characteristics, as well as half of the genetic risk (Taylor, 2021). Second, children with ADHD often have comorbid mental disorders, which are also associated with lower academic performance (Fröjd et al., 2008; Lawrence et al., 2019). By adjusting for comorbid mental disorders, we test whether reduced school performance is specific to ADHD. Third, children may be diagnosed with ADHD because they perform poorly in school, rather than the other way around. We therefore control for early school performance to see how ADHD is associated with progress.

To summarize, the aims of this study are to (1) accurately describe the ADHD deficit in school performance, (2) describe how the ADHD deficit in school performance varies across sex and parental education, and (3) compare the ADHD deficit in different school subjects. We do this using register data on all Norwegian children with appropriate statistical controls, including sibling‐comparisons, which should result in precise and representative analyses.

## METHODS

### Sample

This study comprises all Norwegian inhabitants born between 1997 and 2002 that were alive and living in Norway between age 10 and 16 (*N* = 359,492). The Norwegian national population register includes personal identification numbers of all inhabitants, which allowed us to link data from separate sources. We linked data from publicly funded health services with data on school performance, parental education, and parentage from Statistics Norway. Norwegian school is compulsory up to 10th grade (age 16), meaning the resulting grades and GPAs are largely representative of the population. We identified 344,523 (95.7%) individuals who had GPA registered within 1 year of normed time (age 16) which we included in the main analyses (see Figure S1). Only an additional 14,969 (4.3%) individuals did not have a GPA registered within normed time, which we included in sensitivity analyses. Relatedness data were used to identify 145,051 full siblings nested in 69,765 sibships. The dataset was constructed to maximize the number of children with health data (born 1997 and later) and school performance data (born 2002 and before).

### Measures

#### Exposure: ADHD

Due to the subsidized nature of the Norwegian healthcare system, general practitioners send reimbursement claims to the government each time a patient uses primary care facilities. These data are then indexed in the Norwegian Control and Payment of Health Refunds Database. These claims include the reason for encounter, which is documented with diagnostic codes or symptom codes according to the International Classification of Primary Care version 2 (ICPC‐2, WONCA, 2005). Just like ICD‐10 (WHO, 1992), ICPC‐2 does not differentiate between different kinds of attention disorders such as ADD and ADHD, but instead uses the term *Hyperkinetic Disorder* (code P81). Henceforth, we treat ADHD and hyperkinetic disorder as synonymous. We defined individuals as diagnosed with ADHD if they, between the age of 10 and 16, had at least one contact with the primary care system registered with code P81.

#### Outcome: School performance

At the end of 10th grade, children – normally 16 years old – are graded based on their whole‐year performance and take exams. Grades are integers on a scale from one to six and the grade point average (GPA) are the mean of all grades, including final exams (*M* = 4.12, SD = 0.83). The higher the grade, the better the performance. GPA was standardized to z‐scores (*M* = 0, SD = 1) before analyses, meaning all coefficients can be interpreted as standard deviations. For individual school subjects, we limited our analyses to grades that most pupils share (e.g., excluding electives, see Table S1). For these grades, we standardized using the combined mean and standard deviation of all grades irrespective of school subject (*M* = 4.17, SD = 1.08).

We also used scores from standardized national tests in mathematics and reading, which pupils take in fifth, eight, and ninth grade (approx. 10, 13, and 14 years old, respectively). These were also standardized to z‐scores.

#### Parental education and other demographic information

Statistics Norway provided information on parental education at the time of the child's graduation (i.e., age 16), which we coded into four categories reflecting the highest achieved education of either parent: Master's degree or equivalent (*n* = 53,648), Bachelor's degree or equivalent (*n* = 130,155), high school (*n* = 116,920), or not completed high school (*n* = 30,860), and a fifth category for children with missing information (*n* = 12,569). Statistics Norway also provided information on sex, parentage (for within‐family analyses, see below), birth month, and parity (i.e., maternal birth order).

#### Comorbid psychiatric disorders

Comorbid psychiatric disorders were defined in a similar way to ADHD: at least one contact with the primary care system registered with diagnostic codes for psychiatric disorders between the ages of 10 and 16. For each individual, we counted the total number of unique psychological diagnostic codes (P70 and above, excluding P81) from the ICPC‐2, and entered the count as a categorical variable (truncated at three). In an alternative model, we used separate indicator variables for all individual disorders with a prevalence above 0.1% (see Table S2).

### Statistical analyses

To estimate the ADHD deficit, we calculated bivariate and adjusted associations between ADHD and GPA with linear regression models. The covariates included in the adjusted models were sex, parental education, birth year, parity (truncated at five), and birth month. All of these were treated as categorical variables. The adjusted model only included covariates where reverse causation (and hence potential collider bias) is impossible or unlikely. Early school performance and comorbid mental disorders were therefore included in separate models. We used standardized test scores from fifth grade as a measure of early school performance. This variable had 28,765 missing observations, which were not included in the analyses using this variable. To investigate how the ADHD deficit varied across sex and parental education, we allowed ADHD to interact with sex and parental education in separate models. For comparison, we also estimated a regression model with covariates only (i.e., without ADHD).

The models were then re‐estimated in within‐family models (random‐intercept multilevel models with parents as grouping variable) where we compared full siblings with and without ADHD. Only children with full siblings in the sample were included in these models (*N* = 145,051). Of the 69,765 sibships, 3952 (5.7%) included siblings discordant on ADHD. Covariates that vary within families (sex, parity, birth year, birth month) were included. As most full siblings will have equally educated parents, only the interaction between ADHD and sex was estimated with a within‐family model.

Finally, the adjusted and within‐family models with and without interactions were re‐estimated with each individual school subject as the dependent variable.

We performed sensitivity analyses with several alternative outcomes. First, we re‐estimated all models with standardized tests from eighth and ninth grade as dependent variables. Second, we included the 14,969 individuals who did not have registered GPA within normed time and used logistic regressions to re‐run the adjusted models with missing GPA as the dependent variable.

Analyses were conducted in *R 4.0.3* (R Core Team, 2021), using *tidyverse* (Wickham et al., 2019), *lme4* (Bates et al., 2015) and *emmeans* (Lenth, 2021).

## RESULTS

**FIGURE 1:**
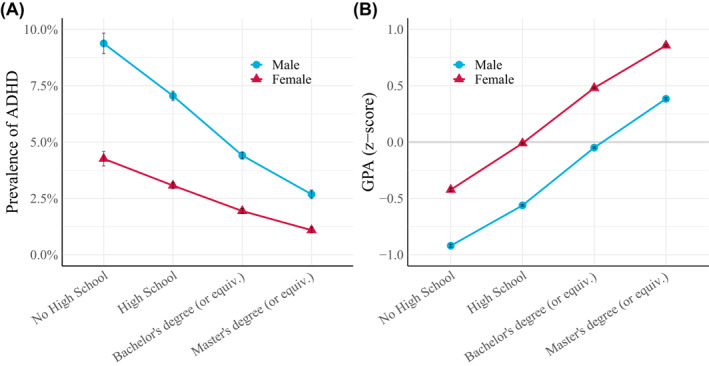
(A) Prevalence of Attention‐Deficit Hyperactivity Disorder (ADHD) (with 95% CIs) across sex and parental education among 331,583 Norwegian children born between 1997 and 2002. ADHD were defined as at least one contact with the primary care system registered with code P81 (hyperkinetic disorder) between age 10 and 16. (B) Standardized GPAs (with 95% CIs) at the end of 10th grade (∼age 16) across sex and parental education for the same sample

Of the 344,152 included children (51% boys), 13,800 (4.01%) were diagnosed with ADHD (see Figure 1). The prevalence was more than twice as high among boys (5.54%) than among girls (2.42%). The prevalence was more than three times higher among children of parents where neither had completed high school (6.87%) compared with children where at least one parent had a Master's degree or equivalent (1.91%).

### The ADHD deficit in GPA

**FIGURE 2:**
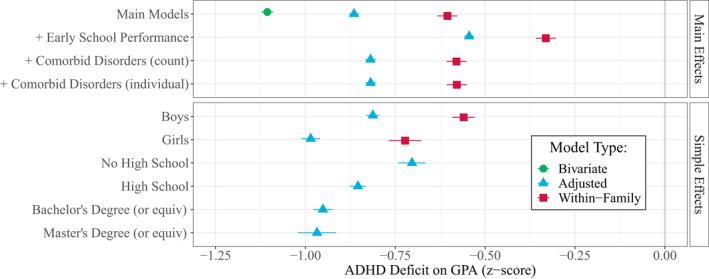
Coefficients (with 95% CIs) showing the Attention‐Deficit Hyperactivity Disorder (ADHD) deficit on GPA (z‐scores) bivariate and adjusted for sex, parental education, birth year, parity, and birth month, and additionally adjusted for early school performance and comorbid disorders. The lower panel shows the ADHD deficit by sex and parental education. As most full siblings will have equally educated parents, we did not include parental education in the within‐family models (hence no interaction between parental education and ADHD in the within‐family model)

ADHD‐affected children's average GPA was 3.24 (SD = 0.75) whereas unaffected children's average GPA was 4.16 (SD = 0.82), meaning children with ADHD had on average −1.11 (95% CI: −1.12, −1.09) standard deviations lower GPA than children without ADHD (see Figure 2). When adjusting for covariates, the difference was reduced to −0.86 (−0.88, −0.85). In the within‐family model, children with ADHD had on average −0.60 (−0.63, −0.58) standard deviations lower GPA than their same‐sex siblings without ADHD.

The ADHD deficit was reduced but still substantial when accounting for earlier school performance. Among pupils who did equally well in fifth grade, those with ADHD had on average −0.54 (−0.56, −0.53) standard deviations lower GPA at the end of 10th grade compared to those without ADHD. Among siblings who did equally well in fifth grade, those with ADHD had on average −0.33 (−0.36, −0.30) standard deviations lower GPA.

Of the 13,800 individuals with ADHD, 2293 (19.8%) had at least one other psychiatric diagnosis. When statistically controlling for other diagnoses, the ADHD deficit was attenuated down from −0.86 to −0.82 (−0.83, −0.80) standard deviations in the adjusted model and from −0.60 to −0.58 (−0.61, −0.55) in the sibling model. This is still larger than the deficit associated with having three or more other registered diagnoses. Entering each individual diagnosis as separate indicator variables yielded similar coefficients (see Figure 2). The ADHD deficit was substantially larger than deficits associated with any other diagnoses (see Table S3‐S4).

The effects of covariates on GPA were only negligibly attenuated compared to a model where ADHD was not included (see Table S3‐S4). For example, in a model without ADHD, girls had on average 0.52 (0.52, 0.53) standard deviations higher GPA than boys, whereas in a model with ADHD, the difference was 0.50 (0.49, 0.50).

### The ADHD deficit across sex and parental education

The ADHD deficit was −0.17 (−0.20, −0.14) standard deviations larger for girls than for boys. As seen in Figure 2 **,** boys with ADHD had on average −0.81 (−0.83, −0.80) standard deviations lower GPA than boys without ADHD, whereas girls with ADHD had on average −0.99 (−1.01, −0.96) lower GPA than girls without ADHD. That is a 22% bigger deficit. The within‐family model had similar results (see Table S4).

Similarly, the ADHD deficit was substantially greater among children of more educated parents, although it did not differ between the two highest education levels as illustrated by the overlapping confidence intervals in Figure 2. Among children of parents who did not complete high school, those with ADHD had on average −0.70 (−0.74, −0.67) standard deviations lower GPA than those without ADHD. Among children with at least one highly educated parent, the deficit was 39% larger, or −0.26 (−0.33, −0.20) standard deviations: Those with ADHD had on average −0.97 (−1.02, −0.92) standard deviations lower GPAs than those without ADHD.

### Which school subjects are most strongly affected by ADHD?

**FIGURE 3:**
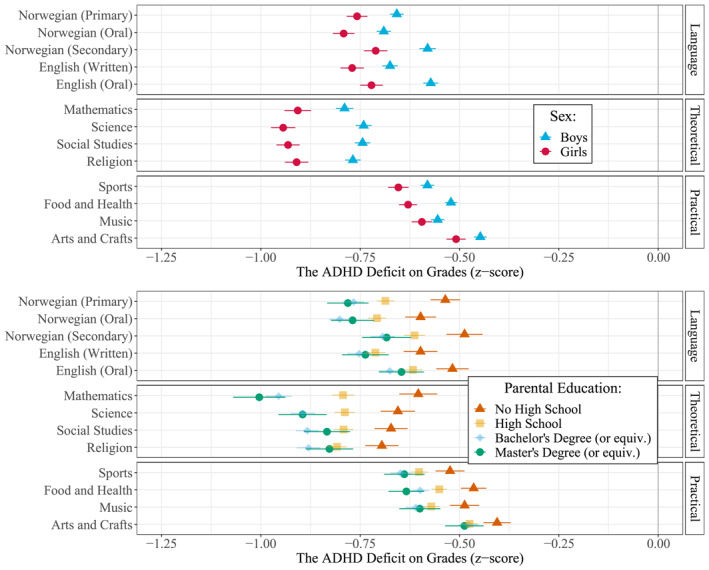
Coefficients (with 95% CIs) showing the relative Attention‐Deficit Hyperactivity Disorder (ADHD) deficit on grades in specific subjects (z‐scores) stratified by sex (top) and parental education (bottom). All coefficients are adjusted for birth year, parity, and birth month, in addition to parental education (top) and sex (bottom)

We repeated the above analyses for each school subject, with similar results (See Figure 3 and Tables S5–S9). Those with ADHD had lower grades in all school subjects compared to those without ADHD. Nonetheless, we found some variation, with the average ADHD deficit varying from −0.47 (−0.48, −0.45) standard deviations in Arts and Crafts to −0.82 (−0.84, −0.81) in Mathematics. To ease interpretation, we can broadly categorize school subjects into three groups: language subjects (i.e., Norwegian and English), theoretical subjects (i.e., Mathematics and Social Studies), and practical subjects (i.e., Sports and Arts/Crafts). The ADHD deficit was largest and most consistent in the theoretical subjects (−0.82 to −0.80), slightly smaller and more variable for language subjects (−0.70 to −0.62), and smaller still for practical subjects (−0.60 to −0.47).

Similar interactions were also observed across each subject: The ADHD deficit was consistently larger for girls than for boys in all subjects. The deficit difference varied from −0.20 (−0.24, −0.17) in Science to −0.04 (−0.07, −0.01) in Music. Regarding interaction effects between ADHD and parental education, we found few differences between the top three education levels, but the ADHD deficit was consistently larger than for children of parents who had not completed high school. The difference in ADHD deficit was particularly pronounced in Mathematics, with the ADHD deficit being −0.40 (−0.48, −0.32) standard deviations larger for children of highly educated parents compared to children of parents who had not completed high school.

**FIGURE 4:**
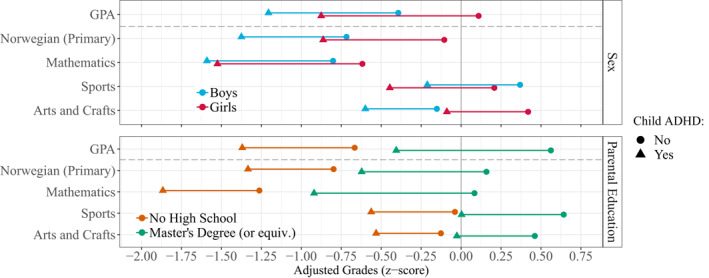
Adjusted mean grades (z‐scores) for those with and without Attention‐Deficit Hyperactivity Disorder (ADHD) in a selection of subjects stratified by sex (top) and parental education (bottom). See Supplementary Figures S2 and S3 for extended versions of these plots with all school subjects

Neither the ADHD deficit, general sex differences, nor parental educational differences are constant across school subjects, which must be considered when interpreting the relative deficits. Figure 4 presents expected grades (z‐scores) for individuals with and without ADHD across sex or parental education (see also Figure S2‐S3). In some school subjects, the sex difference is almost as large as the ADHD deficit. Thus, in school subjects with large sex differences such as Norwegian (i.e., language skills), boys *without* ADHD barely outperformed girls *with* ADHD, despite the ADHD deficit being larger for girls. Because grade differences by parental education are larger than sex differences, this pattern becomes more pronounced for parental education. Children with ADHD and highly educated parents perform better in most school subjects than children without ADHD but with parents who had not completed high school.

### Sensitivity analyses

All analyses were repeated with standardized tests from eighth and ninth grade as outcome variables, which yielded similar results (Tables S10–S17 and Figures S4–S7). Missing GPA was also analyzed with logistic regressions, again with similar results. Children with ADHD were 4.28 (4.08, 4.49) times more likely than unaffected children to not have GPA registered, adjusting for covariates (see Table S18 and Figure S8). There were no statistically significant interaction effects in this set of analyses.

## DISCUSSION

Using large and representative register data, we have found: (1) that the ADHD deficit in school performance is large and evident even when comparing siblings and when adjusting for comorbid disorders and early school performance, (2) that the relative ADHD deficit is somewhat larger for girls and children of highly educated parents but the absolute deficit is smaller, and (3) the ADHD deficit is largest in theoretical school subjects, but still substantial in all school subjects. In the sensitivity analyses, we also found that ADHD increased the likelihood of not being registered with a GPA, meaning the deficit on GPA is a conservative estimate of the association between ADHD and poor school performance.

Even though children diagnosed with ADHD had substantially lower GPAs than other children, with an overall deficit of −1.11 standard deviations, this was partly due to family confounding. When comparing same‐sex siblings and therefore controlling for unobserved family characteristics and genetic similarity, the ADHD deficit was reduced to −0.60 standard deviations. The ADHD deficit remain large after adjusting for potential confounders, but it would nevertheless be premature to conclude that the relationship is causal. For example, siblings share only half of the genetic factors that vary in the population, implying that residual genetic variation may still influence both ADHD and school performance. However, despite both ADHD and school performance being highly heritable (Faraone & Larsson, 2019), the attenuation of the ADHD deficit was relatively small in the within‐family model compared to the fully adjusted model, indicating that an association would plausibly exist even with full adjustment for genetic factors.

A second caveat to causal interpretations is reverse causation: ADHD and school performance pose a chicken and egg problem in that children must show age‐inappropriate levels of inattention and/or hyperactivity in at least two contexts to be eligible for ADHD diagnosis (APA, 2013; WHO, 1992). For children, school will likely be one of these contexts, meaning that many may have received an ADHD diagnosis because they struggled in school. While our data does not permit us to completely discount reverse causation, controlling for early school performance should give an indication: If poor early school performance was the primary reason children were diagnosed with ADHD, then the association between ADHD and later school performance should disappear or be substantially reduced when adjusting for early school performance. Instead, we found that among children with similar performance in fifth grade, those with ADHD still had on average −0.54 standard deviations lower GPAs at the end of 10th grade compared to those without ADHD. In other words, ADHD is not only associated with poor school performance, but also with worsening school performance relative to peers. This is not consistent with poor early school performance being the primary reason children are diagnosed with ADHD.

Most of the ADHD deficit remained, but it was attenuated by about a third from −0.86 down to −0.54. It can be tempting to interpret this to mean that a third of the ADHD deficit is due to selection bias. We caution against this interpretation, as we cannot disentangle the degree to which the attenuation is caused by selection bias (poor early school performance → ADHD) or mediation (ADHD → poor early school performance), and adjusting for mediators will underestimate the effect.

A third caveat is that ADHD often covaries with other mental disorders, such as depression and anxiety, which might confound the association between ADHD and school performance. We found that adjusting for other mental disorders had only a negligible impact on the ADHD deficit, meaning the ADHD deficit is generated independently of other mental disorders. This mirrors earlier research finding that ADHD impacts school performance independently of comorbid conduct disorders (Daley & Birchwood, 2010). Overall, we find that the ADHD deficit is not easily attributed to other factors, and that ADHD therefore reflects an independent risk factor of poor school performance.

The effect of covariates on GPA remained similar in models with and without ADHD, suggesting their effect on GPA is not partly mediated by ADHD diagnoses. Differences in school performance across, e.g., sex and parental education must therefore be explained by other factors.

The *relative* ADHD deficit was larger in groups with lower prevalence of ADHD: It was 22% larger for girls than for boys, and 39% larger for children with highly educated parents compared with children of parents who did not complete high school. Similar findings were found for individual school subjects. There are at least two ways of interpreting the larger relative deficit in low‐prevalence groups: First, the threshold for receiving an ADHD diagnosis may be higher for girls and for children of highly educated parents, which would result in the average diagnosed case being more severe and consequently would impact school performance more. Whether there are true differences or merely different thresholds for receiving a diagnosis remain debated (e.g., Slobodin & Davidovitch, 2019), but one representative study found sex differences to result from true differences in mean and variance of ADHD symptom severity, not selection bias (Arnett et al., 2015). The second and in our opinion more likely interpretation is that girls and children of highly educated parents appear more impacted by ADHD because their undiagnosed peers have relatively higher grades. In fact, the differences in school performance associated with parental education are often bigger than the relative ADHD deficits, meaning that ADHD‐affected children of highly educated parents on average outperform children *without* ADHD with parents who did not complete high school (see Figure 4). Likewise, in some school subjects (e.g., Norwegian), girls with ADHD perform similarly to boys *without* ADHD. In terms of absolute performance, then, girls and children of highly educated parents are less impacted by ADHD.

We found that the ADHD deficit was large in all school subjects. This mirrors Jangmo et al. (2019), who reported similar findings from Swedish registers (see Appendix S1 for a more detailed comparison). We also investigated interactions and found it particularly large between ADHD and parental education in Mathematics. The interaction between sex and ADHD were mostly similar across school subjects, despite varying sex differences. Large ADHD deficits in all school subjects suggest that the effect of ADHD is largely mediated through general factors shared across school subjects. We do not know what these are, but they could relate to classroom size, organisation of homework, or emotion regulation (e.g., Daley & Birchwood, 2010; Rushton et al., 2020). Potential interventions should therefore target general factors that impact school performance in a way that is shared across school subjects. However, if limited resources forces interventions to target specific school subjects, then the potential for improvement appears to be largest in theoretical subjects such as Mathematics or Science, where the ADHD deficit was largest.

### Strengths and limitations

Population‐wide register studies like this have numerous strengths (Thygesen & Ersbøll, 2014). First, unlike clinic‐referred samples and cohort samples, this study does not suffer from non‐random attrition and is much less affected by selection bias. In addition to unrepresentative results, selection bias can systematically bias estimates when the investigated variables are associated with likelihood of participation (i.e., collider bias: Munafò et al., 2018). The subsidized and equal‐access nature of the Norwegian healthcare system means cases are unlikely to go unregistered, and only a few children (4.3%) did not have GPA registered (which we included in sensitivity analyses), meaning this study captures a representative picture of the association between ADHD and school performance. Second, register studies have very large sample sizes, which results in narrow confidence intervals and consequently high statistical power. Even large cohort studies can have few participants satisfying several criteria, such as being girls with ADHD and highly educated parents, which would result in low power and large confidence intervals even if the original cohort sample was large (Button et al., 2013). The difference in clinical consequences between the ends of large confidence intervals can be considerable, and small but meaningful differences may go undetected (Funder & Ozer, 2019; Götz et al., 2021; Schönbrodt & Perugini, 2013). Large register studies, on the other hand, can accurately estimate small differences in effects between subgroups, even for relatively rare disorders.

Nevertheless, this study has some limitations. First, diagnoses are all‐or‐none, meaning we are unable to attribute the ADHD deficit to the different facets of ADHD (e.g., inattention vs. impulsivity) or different symptom severities. Second, we only have data on primary health care visits, not medication use. Medication is common among Norwegian children with ADHD (Karlstad et al., 2017) and has been shown to have a positive effect on school performance (Jangmo et al., 2019), meaning our analyses may underestimate the size of the ADHD deficit. Third, physicians often register only one diagnostic code per visit, meaning the data do not adequately capture concurrent comorbidity, only temporal comorbidity. Fourth, we did not observe individuals before age 10. Nonetheless, because parents would often need renewed medical certificates or prescriptions for medications, it is unlikely that individuals who received their first diagnosis before the observation period would not be re‐registered in the observation period. Finally, some relevant variables, such as cognitive ability, are not available in administrative register data and could therefore only be accounted for indirectly – and imperfectly – through early school performance (Borghans et al., 2016).

## CONCLUSION

We have found that the ADHD deficit in school performance is large, apparent in all school subjects, and not easily attributable to other factors. This strongly suggests that ADHD symptoms is an important risk factor for poor school performance which must be addressed directly independent of sex, parental education, early school performance, and other psychiatric disorders. Because it affects performance in all school subjects, ADHD must ideally be addressed by intervening on factors that affect school performance broadly, although interventions targeting theoretical subjects like Mathematics may be most effective given limited resources.

## CONFLICT OF INTEREST

The authors have declared that they have no competing or potential conflicts of interest.

## ETHICAL CONSIDERATION

The study was approved by the Regional Committee for Medical and Health Research Ethics.

## AUTHOR CONTRIBUTIONS

**Hans Fredrik Sunde:** Conceptualization; Data curation; Formal analysis; Investigation; Methodology; Visualization; Writing – original draft; Writing – review & editing. **Thomas H. Kleppestø:** Conceptualization; Investigation; Methodology; Writing – review & editing. **Kristin Gustavson:** Conceptualization; Investigation; Methodology; Writing – review & editing. **Magnus Nordmo:** Conceptualization; Investigation; Methodology; Writing – review & editing. **Bjørn‐Atle Reme:** Conceptualization; Investigation; Methodology; Writing – review & editing. **Fartein A. Torvik:** Conceptualization; Data curation; Funding acquisition; Investigation; Methodology; Project administration; Resources; Supervision; Writing – review & editing.

## Supporting information

Supplementary Material S1

## Data Availability

The register data can be accessed by application to the Regional Committee for Medical and Health Research Ethics in Norway, Statistics Norway, and the Norwegian Directorate of Health. Our ethical approval does not open for storage of data on an individual level in repositories or journals.
