# Supplementary material for: The ADHD deficit in school performance across sex and parental education: A prospective sibling‐comparison register study of 344,152 Norwegian adolescents
Source: JCPP Adv. 2022 Feb 12;2(1):e12064. doi: 10.1002/jcv2.12064 (PMC10242882; doi:10.1002/jcv2.12064)
Supplement: Supplementary file 1 — Supplementary Material S1 [file JCV2-2-e12064-s001.zip › Supporting Information/Appendix S1_ComparisonJangmo.docx]

Appendix: Comparison with Jangmo et al (2019)

**The ADHD deficit in school performance across sex and parental education: a prospective sibling-comparison register study of 344,152 Norwegian adolescents**

*Hans Fredrik Sunde (*), Thomas H. Kleppestø, Kristin Gustavson, Magnus Nordmo, Bjørn-Atle Reme, Fartein Ask Torvik*

Just like our study, Jangmo et al (2019)^[[1]](#footnote-1)^ compared the association between ADHD and grades across different school subjects among similarly aged Swedish adolescents, which they presented in Supplementary Table S3. The list of school subjects differs slightly from our study owing to the differences between the Norwegian and Swedish school systems. For example, Norway have gathered all the natural sciences into one school subject, whereas Sweden has separate school subjects for Biology, Chemistry, Physics, and Technology. To compare our result with those of Jangmo et al, we averaged the coefficients where multiple school subjects in one study matched onto a single school subject in the other (see R script at the end of this document). Jangmo also included weighted tests in their list, which we did not include in our comparison.

The grading system also differs between Sweden and Norway, and because Jangmo et al did not standardize the outcome variables prior to analysis^[[2]](#footnote-2)^, the coefficients are not directly comparable. However, they report means and standard deviations for the ADHD and non-ADHD group, as well as the observed prevalence of ADHD (4.4%). This allows us to easily calculate the combined standard deviations for each school subjects, and hence calculate what the coefficients would have been if they had used a z-score of the outcome variables instead (rounding errors notwithstanding). This allows for a direct comparison of their findings with our findings.

To do this, one must first calculate the combined means, $\bar{X}_{c}$, of the two groups (ADHD vs. Non-ADHD) for each school subject. This is done my simply weighting the two means by the relative group size:

$$\bar{X}_{c}=p\bar{X}_{1}+\left( 1-p \right)\bar{X}_{2}$$

where *p* is the proportion of people who are in group 1 (i.e., prevalence of ADHD, 0.044) and $\bar{X}_{1}$ and $\bar{X}_{2}$ are the means of each group. We can then calculate the combined standard deviations, $\sigma_{c}$, for each school subject with the following equation:

$$\sigma_{c}=\sqrt{p(\sigma_{1}^{2}+\left( \bar{X}_{1}-\bar{X}_{c} \right)^{2})+(1-p)(\sigma_{2}^{2}+\left( \bar{X}_{2}-\bar{X}_{c} \right)^{2})}$$

where $\sigma_{1}$ and $\sigma_{2}$ are the standard deviations of each group.

In our study, we standardized using the mean and standard deviation for all grades regardless of school subject, which allowed us to retain information on relative performance in each school subject. To make the coefficients from Jangmo et al (2019) comparable, we first took the average standard deviation of the grades in all school subjects (excluding the weighted tests), before dividing the unstandardized adjusted coefficients by this average:

$$\beta_{z_{i}}=\frac{\beta_{i}}{\bar{\sigma}_{c}}$$

where $i$ is the different school subjects, $\beta_{i}$ is the original coefficient, $\beta_{z_{i}}$ is the new, standardized coefficient, and $\bar{\sigma}_{c}$ is the average standard deviation of all grades regardless of school subject.

We then plotted their transformed coefficients together with the corresponding coefficients in our study (reported in Supplementary Table S5). We find that the associations are similar in strength, but with less systematic variation between school subjects.


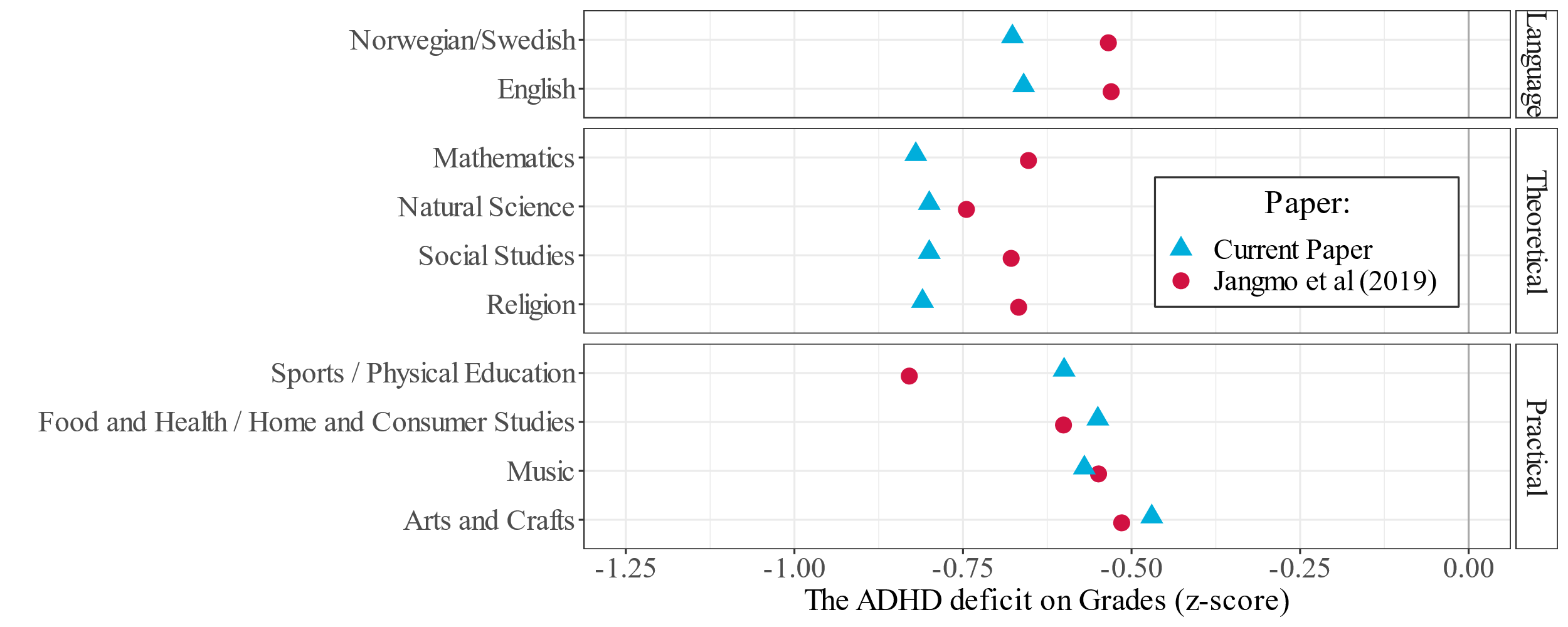


To reproduce this figure, see the R script in the accompanying supplementary materials (zip-file)

1. Jangmo et al (2019). Attention-Deficit/Hyperactivity Disorder, School Performance, and Effect of Medication. *Journal of the American Academy of Child & Adolescent Psychiatry*, *58*(4), 423-432. <https://doi.org/10.1016/j.jaac.2018.11.014> [↑](#footnote-ref-1)
2. They did also report standardized coefficients, but not in a way that makes them directly comparable to our coefficients. [↑](#footnote-ref-2)
