# Supplementary material for: The ADHD deficit in school performance across sex and parental education: A prospective sibling‐comparison register study of 344,152 Norwegian adolescents
Source: JCPP Adv. 2022 Feb 12;2(1):e12064. doi: 10.1002/jcv2.12064 (PMC10242882; doi:10.1002/jcv2.12064)
Supplement: Supplementary file 1 — Supplementary Material S1 [file JCV2-2-e12064-s001.zip › Supporting Information/Supplementary Figures.docx]

SUPPLEMENTARY FIGURES

# The ADHD deficit in school performance across sex and parental education: a prospective sibling-comparison register study of 344,152 Norwegian adolescents

*Hans Fredrik Sunde (*), Thomas Kleppestø, Kristin Gustavson,
Magnus Nordmo, Bjørn-Atle Reme, Fartein Ask Torvik,*

Table of Contents

[Figure S1: Sample Determination Flowchart 2](#_Toc76143268)

[Figure S2: Adjusted Grades by Sex 3](#_Toc76143269)

[Figure S3: Adjusted Grades by Parental Education 4](#_Toc76143270)

[Figure S4: Mathematics, 8^th^ Grade 5](#_Toc76143271)

[Figure S5: Reading, 8^th^ Grade 6](#_Toc76143272)

[Figure S6: Mathematics, 9^th^ Grade 7](#_Toc76143273)

[Figure S7: Reading, 9^th^ Grade 8](#_Toc76143274)

[Figure S8: Registered GPA (Logistic Regressions) 9](#_Toc76143275)

# Figure S1: Sample Determination Flowchart

Population register of Norway
N = 8 423 884

Other age group
N = 8 011 738

Born between 1997 and 2002
N = 412 146

Dead before graduation
N = 2 174

Alive at age 16
N = 409 972

Living abroad during observational period
N = 50 480

Living in Norway from 10 to 16
N = 359 492

Missing GPA

N = 15 293

Registered with GPA
N = 344 199

GPA not registered between age 15 and 17

N = 47

Registered with GPA between age 15 and 17
N = 344 152

Figure S1: Sample Determination Flowchart

# Figure S2: Adjusted Grades by Sex


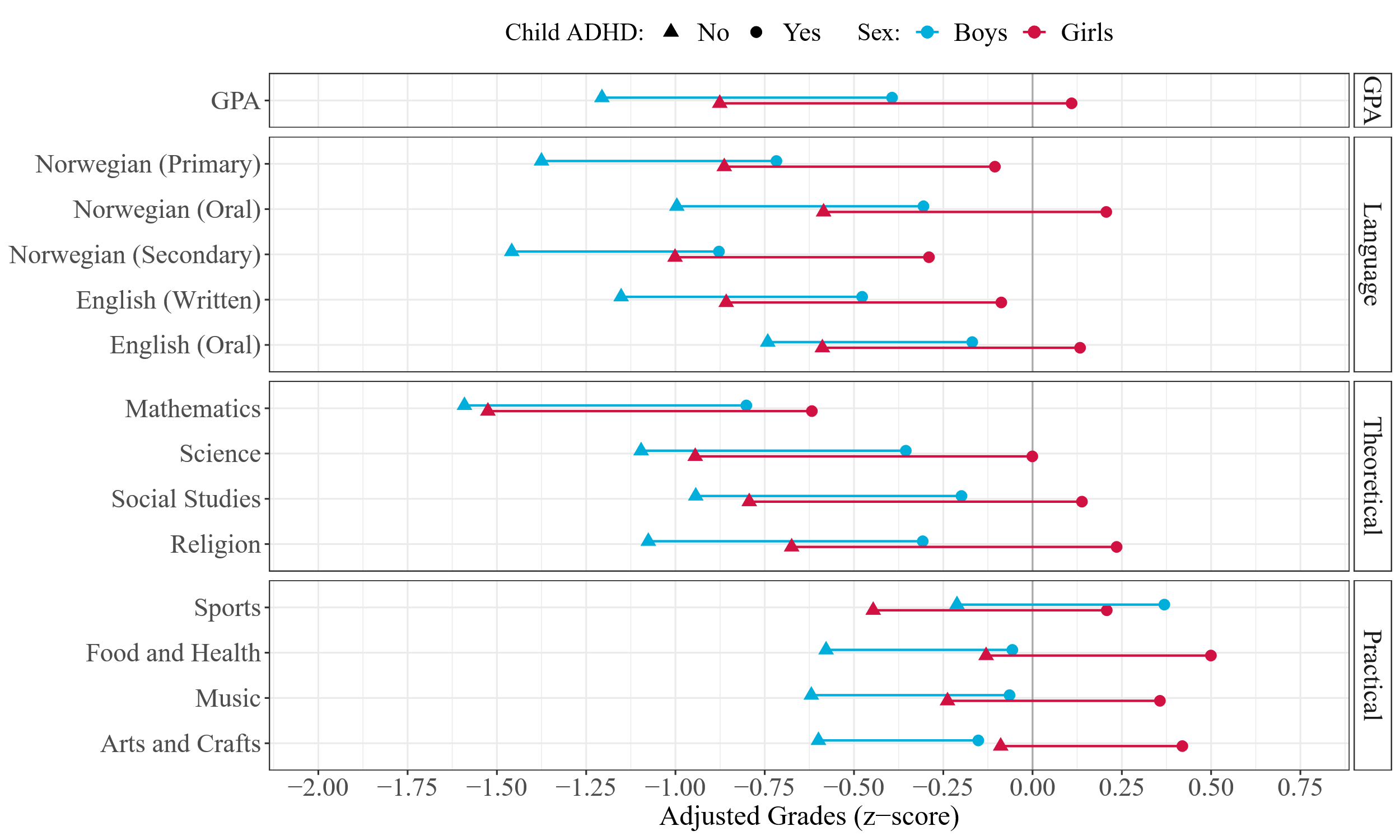


Figure S2: Adjusted mean grades (z-scores) for those with and without ADHD in a selection of subjects stratified by sex.

# Figure S3: Adjusted Grades by Parental Education


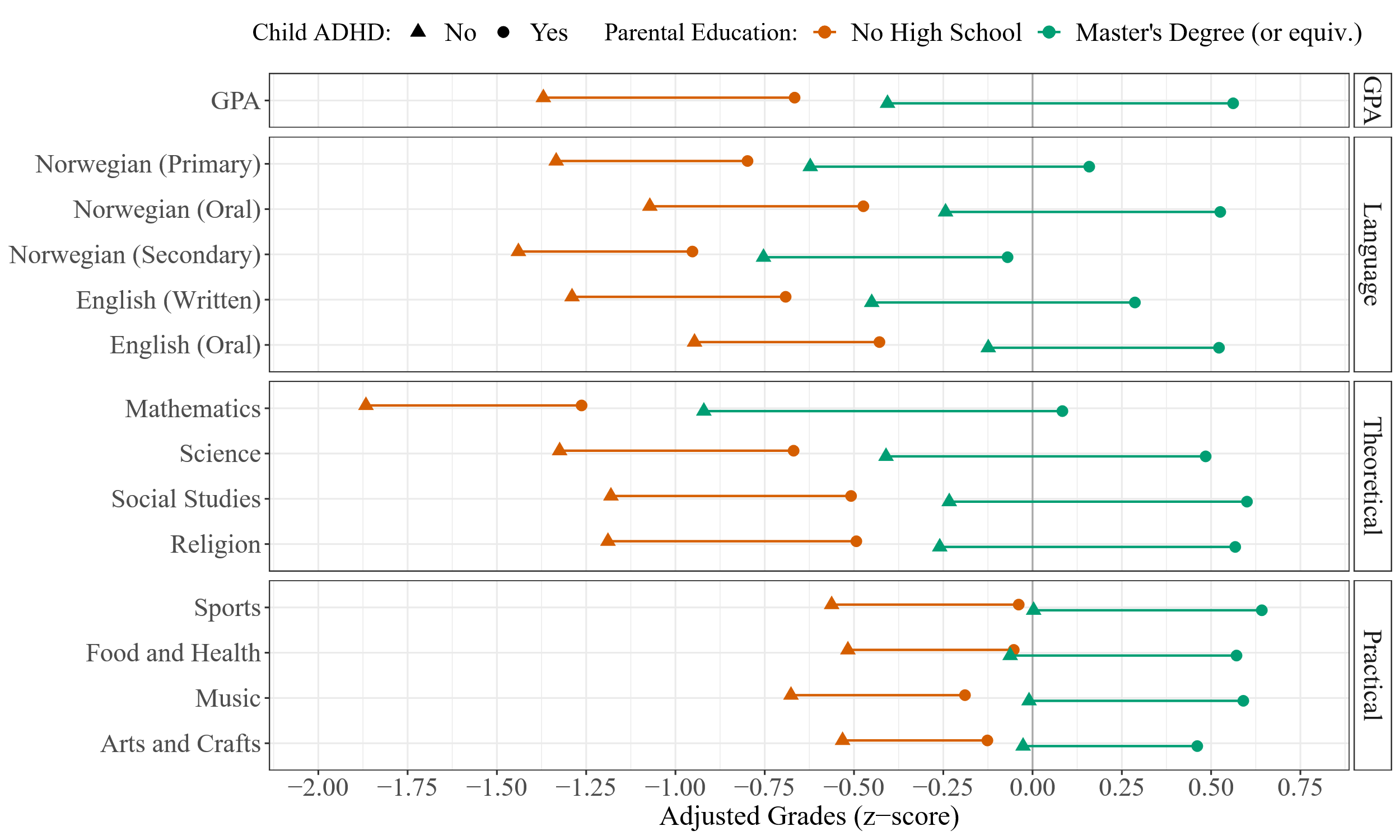


Figure S3: Adjusted mean grades (z-scores) for those with and without ADHD in a selection of subjects stratified by parental education (the most and least educated).

# Figure S4: Mathematics, 8^th^ Grade


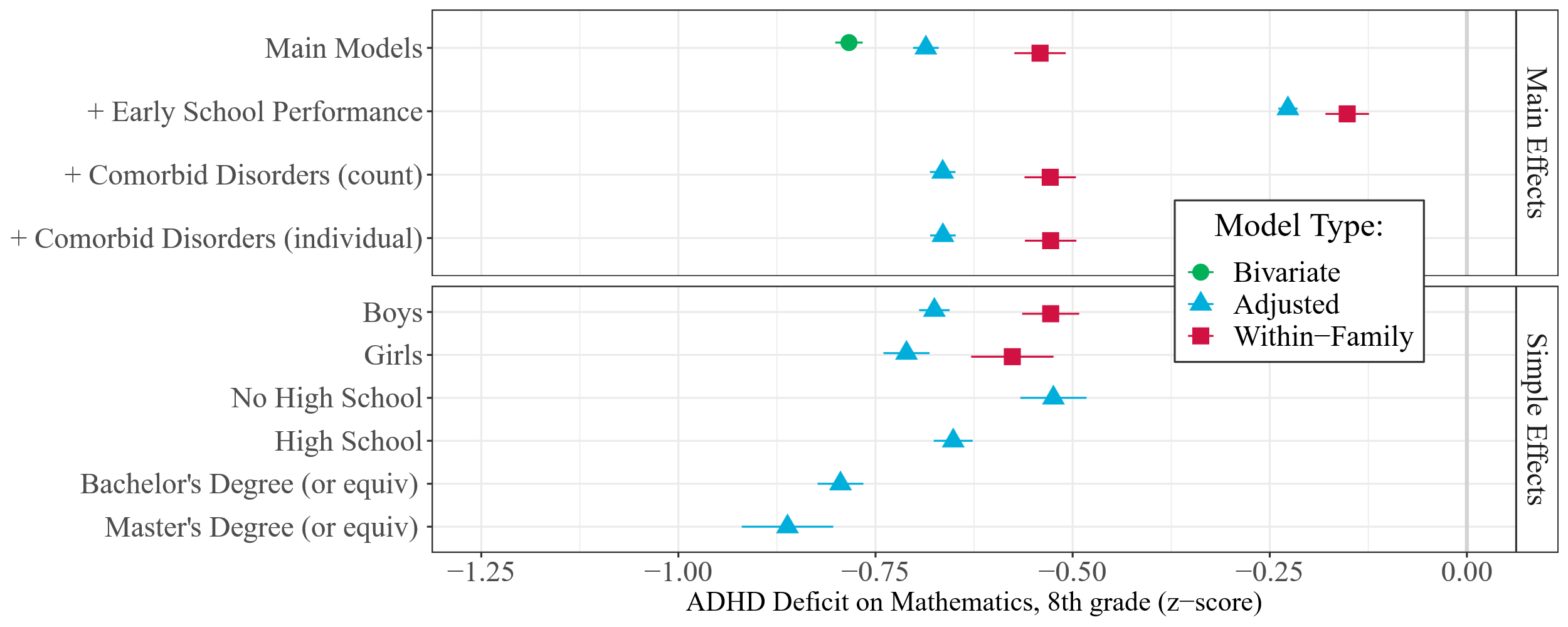


Figure S4: Coefficients (with 95% CIs) showing the ADHD deficit on standardized mathematics tests in 8^th^ grade (z-scores) bivariate and adjusted for sex, parental education, birth year, parity, and birth month, and additionally adjusted for early school performance and comorbid disorders. The lower panel shows the ADHD deficit by sex and parental education. As most full siblings will have equally educated parents, we did not include parental education in the within-family models (hence no interaction between parental education and ADHD in the within-family model).

# Figure S5: Reading, 8^th^ Grade


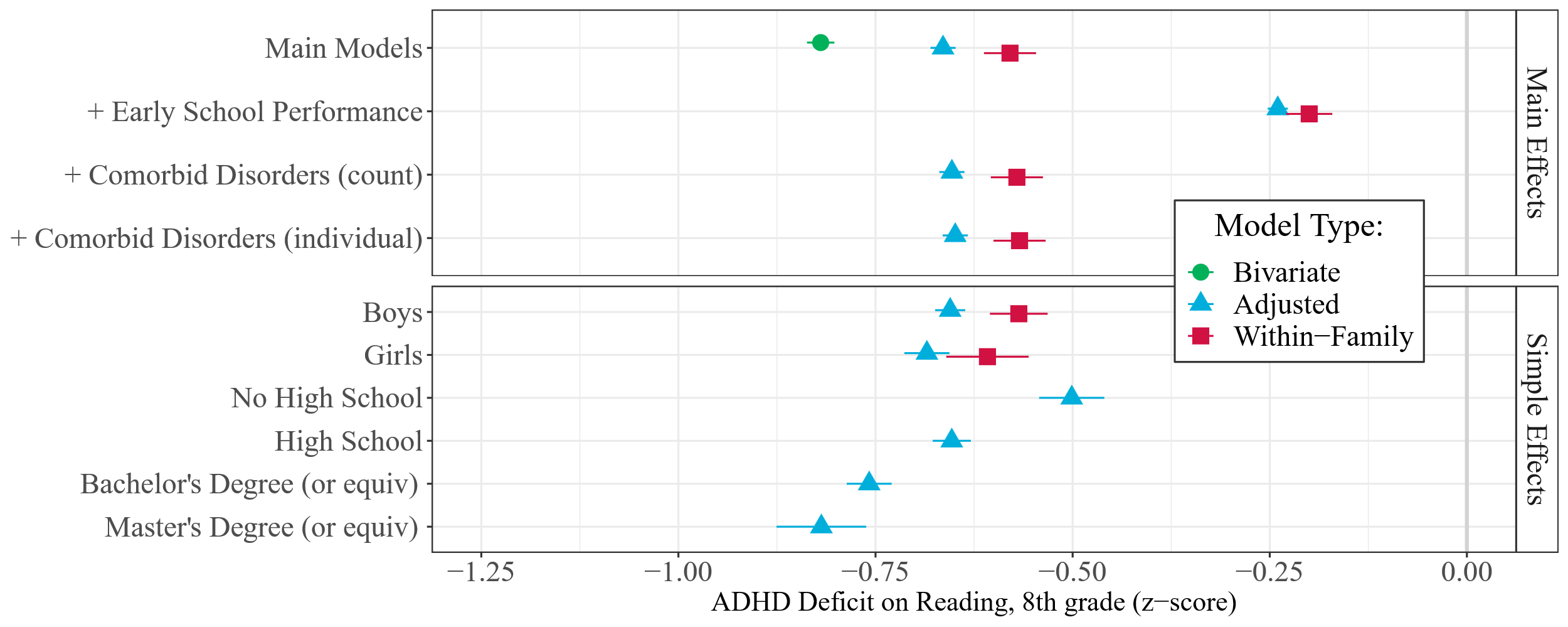


Figure S5: Coefficients (with 95% CIs) showing the ADHD deficit on standardized reading tests in 8^th^ grade (z-scores) bivariate and adjusted for sex, parental education, birth year, parity, and birth month, and additionally adjusted for early school performance and comorbid disorders. The lower panel shows the ADHD deficit by sex and parental education. As most full siblings will have equally educated parents, we did not include parental education in the within-family models (hence no interaction between parental education and ADHD in the within-family model).

# Figure S6: Mathematics, 9^th^ Grade


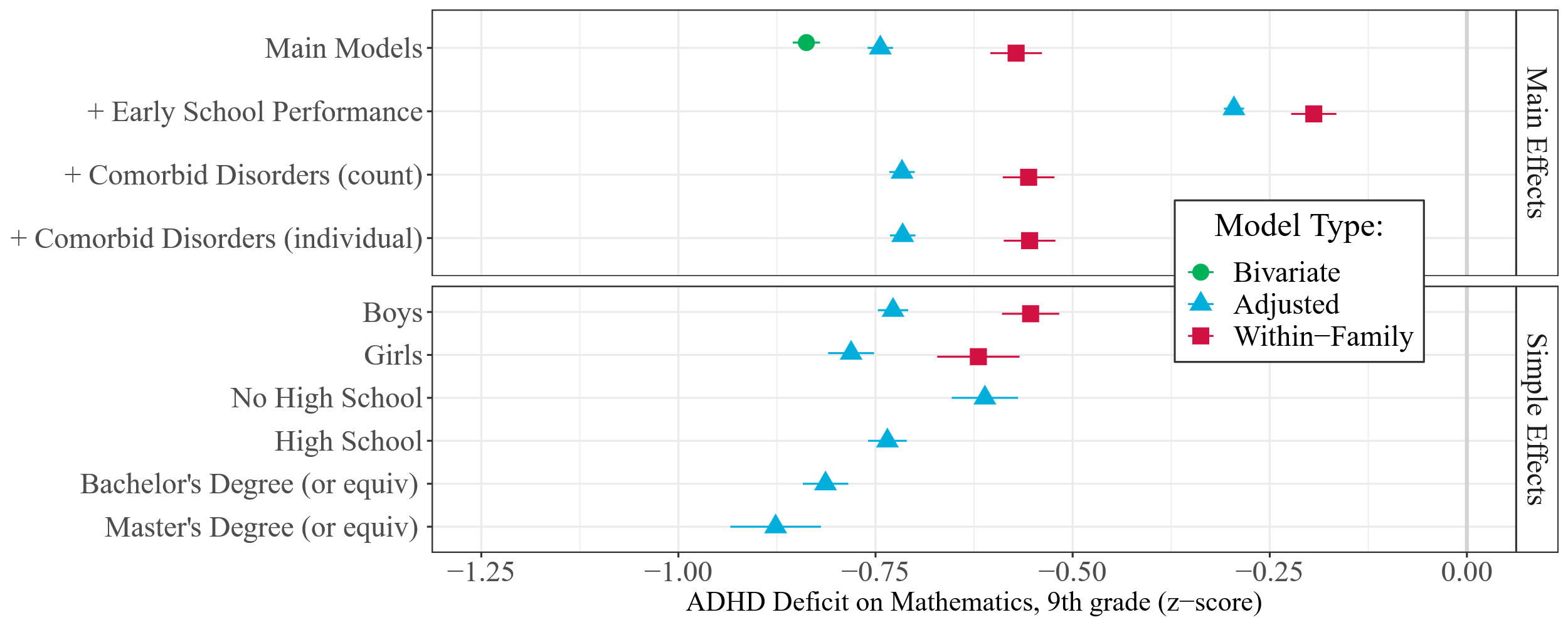


Figure S6: Coefficients (with 95% CIs) showing the ADHD deficit on standardized mathematics tests in 9^th^ grade (z-scores) bivariate and adjusted for sex, parental education, birth year, parity, and birth month, and additionally adjusted for early school performance and comorbid disorders. The lower panel shows the ADHD deficit by sex and parental education. As most full siblings will have equally educated parents, we did not include parental education in the within-family models (hence no interaction between parental education and ADHD in the within-family model).

# Figure S7: Reading, 9^th^ Grade


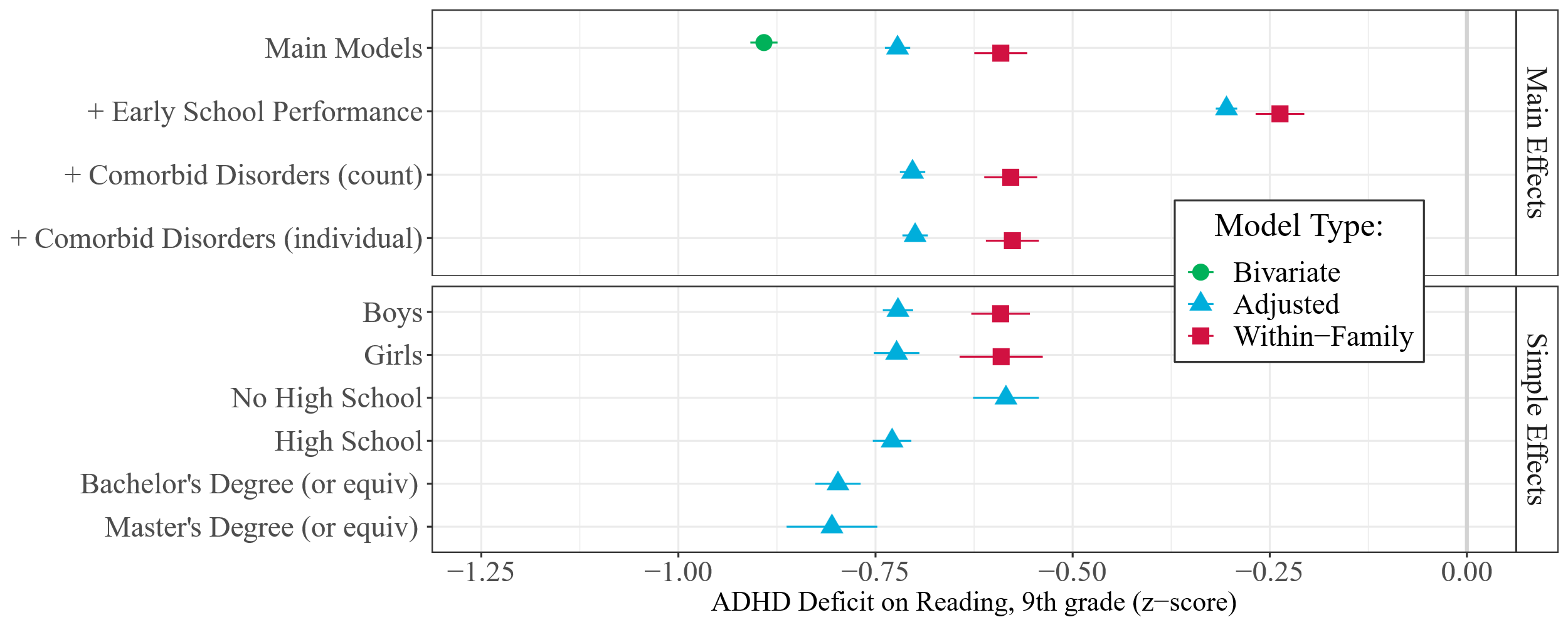


Figure S7: Coefficients (with 95% CIs) showing the ADHD deficit on standardized reading tests in 9^th^ grade (z-scores) bivariate and adjusted for sex, parental education, birth year, parity, and birth month, and additionally adjusted for early school performance and comorbid disorders. The lower panel shows the ADHD deficit by sex and parental education. As most full siblings will have equally educated parents, we did not include parental education in the within-family models (hence no interaction between parental education and ADHD in the within-family model).

# Figure S8: Registered GPA (Logistic Regressions)


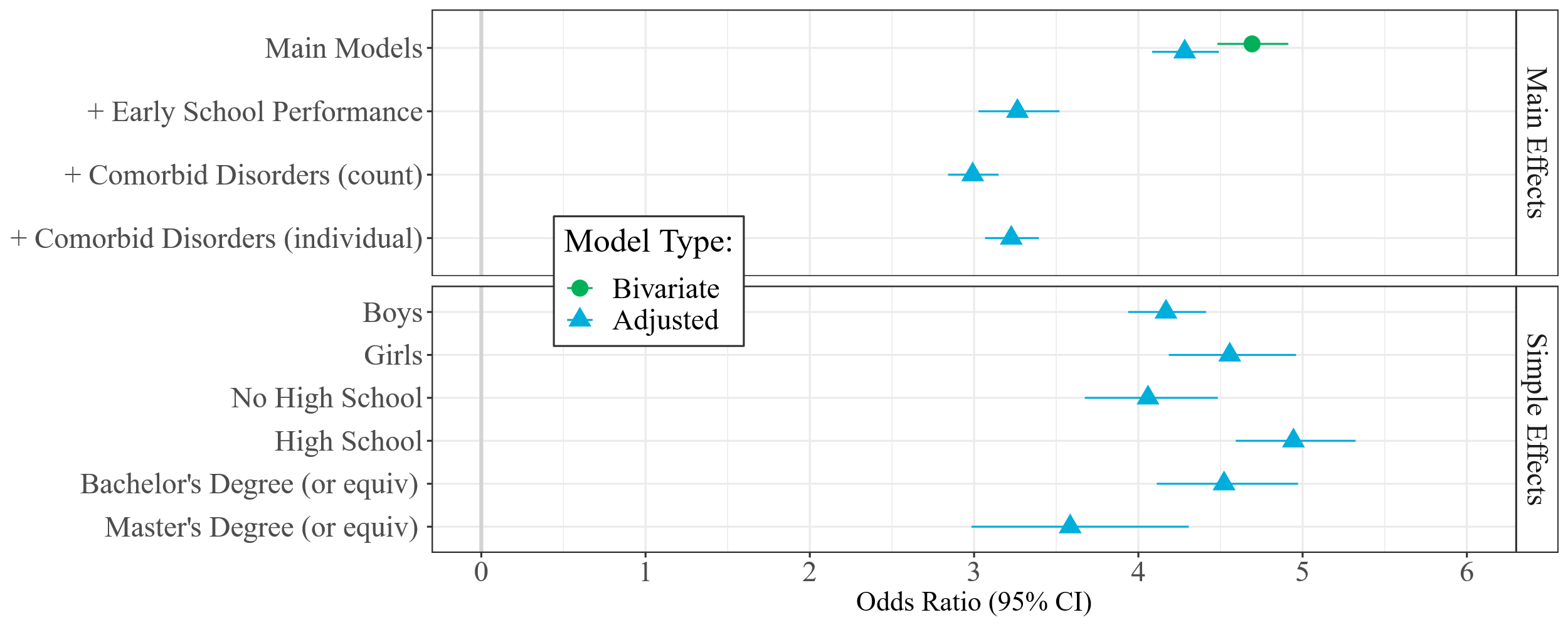


Figure S8: Odds Ratios (with 95% CIs) showing how much more likely children with ADHD are to not have registered GPA (i.e. registered GPA = 0, not registered GPA = 1), bivariate and adjusted for sex, parental education, birth year, parity, and birth month, and additionally adjusted for early school performance and comorbid disorders. The lower panel shows the odds ratios stratified by sex and parental education.
