# Supplementary material for: The ADHD deficit in school performance across sex and parental education: A prospective sibling‐comparison register study of 344,152 Norwegian adolescents
Source: JCPP Adv. 2022 Feb 12;2(1):e12064. doi: 10.1002/jcv2.12064 (PMC10242882; doi:10.1002/jcv2.12064)
Supplement: Supplementary file 1 — Supplementary Material S1 [file JCV2-2-e12064-s001.zip › Supporting Information/Supplementary Tables/Overview of Supplementary Tables.docx]

Overview of Supplementary Tables

**The ADHD deficit in school performance across sex and parental education: a prospective sibling-comparison register study of 344,152 Norwegian adolescents**

*Hans Fredrik Sunde (*), Thomas H. Kleppestø, Kristin Gustavson,
 Magnus Nordmo, Bjørn-Atle Reme, Fartein Ask Torvik*

- **Descriptive Statistics**
  - - **Table S1:** List of School Subjects and Grades
    - **Table S2:** Prevalence of Different Disorders
- **Regression Tables:**
  - GPA
    - **Table S3:** Bivariate and Adjusted Models
    - **Table S4:** Sibling Models
  - Individual School Subjects
    - **Table S5:** Fully Adjusted Models
    - **Table S6:** Sibling Models
    - **Table S7:** Interaction with Sex (Adjusted Models)
    - **Table S8:** Interaction with Parental Education (Adjusted Models)
    - **Table S9:** Interaction with Sex (Sibling Models)
  - Standardized Tests
    - **Table S10:** Mathematics, 8^th^ grade (Bivariate and Adjusted Models)
    - **Table S11:** Mathematics, 8^th^ grade (Sibling Models)
    - **Table S12:** Reading, 8^th^ grade (Bivariate and Adjusted Models)
    - **Table S13:** Reading, 8^th^ grade (Sibling Models)
    - **Table S14:** Mathematics, 9^th^ grade (Bivariate and Adjusted Models)
    - **Table S15:** Mathematics, 9^th^ grade (Sibling Models)
    - **Table S16:** Reading, 9^th^ grade (Bivariate and Adjusted Models)
    - **Table S17:** Reading, 9^th^ grade (Sibling Models)
  - Registered GPA
    - **Table S18:** Bivariate and Adjusted Logistic Regression Models
