# Supplementary material for: The ADHD deficit in school performance across sex and parental education: A prospective sibling‐comparison register study of 344,152 Norwegian adolescents
Source: JCPP Adv. 2022 Feb 12;2(1):e12064. doi: 10.1002/jcv2.12064 (PMC10242882; doi:10.1002/jcv2.12064)
Supplement: Supplementary file 1 — Supplementary Material S1 [file JCV2-2-e12064-s001.zip › Supporting Information/Supplementary Tables/Table S1.docx]

# Table S1: List of School Subjects and Grades

| **Table S1** | | |
| --- | --- | --- |
| List of School Subjects and Grades* | | |
|  | Mean | *SD* |
| GPA | 4.12 | 0.83 |
|  |  |  |
| **Language** |  |  |
| Norwegian (Primary)* | 3.88 | 1.02 |
| Norwegian (Secondary) | 3.68 | 1.00 |
| Norwegian (Oral) | 4.25 | 1.04 |
| English (Written) | 3.97 | 1.08 |
| English (Oral) | 4.25 | 1.04 |
|  |  |  |
| **Theoretical** |  |  |
| Mathematics | 3.58 | 1.23 |
| Science | 4.14 | 1.15 |
| Social Studies | 4.27 | 1.11 |
| Religion | 4.24 | 1.13 |
|  |  |  |
| **Practical** |  |  |
| Sports | 4.57 | 0.92 |
| Food and Health | 4.52 | 0.87 |
| Arts and Crafts | 4.40 | 0.92 |
| Music | 4.43 | 0.96 |
|  |  |  |
| *Combined Mean*** | *4.17* | *1.08* |
|  | | |

**This list only includes the school subjects that all pupils normally would have. The GPA also comprise school subjects that varies between pupils, such as electives and foreign language subjects. Some pupils take French, others take German. Additionally, grades from final exams are also included in the GPA. However, pupils only take two final exams (one written, one oral) in randomly selected subjects, meaning they vary between pupils.*

*** Norway has two official written versions of Norwegian (“Bokmål” and “Nynorsk”), and pupils receive a grade in both. Which one is considered primary depends on the pupils main language form and/or geographical area. (*[*Read more at Wikipedia*](https://en.wikipedia.org/wiki/Norwegian_language#Bokm%C3%A5l_and_Nynorsk)*)*

**** The mean and SD of all subjects combined were used for standardization. The SD is larger for the overall mean than for the GPA because the GPA will tend to regress towards the mean resulting in fewer extreme grades and consequently less variation around GPA.*
