# Supplementary material for: The ADHD deficit in school performance across sex and parental education: A prospective sibling‐comparison register study of 344,152 Norwegian adolescents
Source: JCPP Adv. 2022 Feb 12;2(1):e12064. doi: 10.1002/jcv2.12064 (PMC10242882; doi:10.1002/jcv2.12064)
Supplement: Supplementary file 1 — Supplementary Material S1 [file JCV2-2-e12064-s001.zip › Supporting Information/Supplementary Tables/Table S10.html]

Table S10: Regression Table – Mathematics, 8th grade (Bivariate and Adjusted Models)

| Dependent Variable: Test Score (z-score) | Bivariate: ADHD | Covariates Only | Fully Adjusted | + Number of Diagnoses | + Specific Diagnoses | + Early School Performance | Interaction w/ Sex | Interaction w/ Parental Education |
| Predictors | Estimates (95% CIs) | Estimates (95% CIs) | Estimates (95% CIs) | Estimates (95% CIs) | Estimates (95% CIs) | Estimates (95% CIs) | Estimates (95% CIs) | Estimates (95% CIs) |
| ADHD (P81) | -0.78 (-0.80 – -0.77) |  | -0.69 (-0.70 – -0.67) | -0.66 (-0.68 – -0.65) | -0.66 (-0.68 – -0.65) | -0.23 (-0.24 – -0.21) | -0.68 (-0.69 – -0.66) | -0.52 (-0.57 – -0.48) |
| Sex: Boys |  | *Reference* | *Reference* | *Reference* | *Reference* | *Reference* | *Reference* | *Reference* |
| Sex: Girls |  | -0.12 (-0.13 – -0.12) | -0.14 (-0.15 – -0.14) | -0.14 (-0.14 – -0.13) | -0.14 (-0.15 – -0.13) | -0.08 (-0.09 – -0.08) | -0.14 (-0.15 – -0.14) | -0.14 (-0.15 – -0.14) |
| Parental Education: No High School |  | *Reference* | *Reference* | *Reference* | *Reference* | *Reference* | *Reference* | *Reference* |
| Parental Education: High School |  | 0.30 (0.29 – 0.32) | 0.29 (0.28 – 0.30) | 0.29 (0.28 – 0.30) | 0.29 (0.28 – 0.30) | 0.07 (0.06 – 0.08) | 0.29 (0.28 – 0.30) | 0.30 (0.29 – 0.31) |
| Parental Education: Bachelor's Degree (or equiv) |  | 0.70 (0.68 – 0.71) | 0.67 (0.66 – 0.68) | 0.67 (0.66 – 0.68) | 0.67 (0.66 – 0.68) | 0.18 (0.17 – 0.19) | 0.67 (0.66 – 0.68) | 0.69 (0.67 – 0.70) |
| Parental Education: Master's Degree (or equiv) |  | 1.07 (1.06 – 1.08) | 1.04 (1.02 – 1.05) | 1.03 (1.02 – 1.04) | 1.03 (1.02 – 1.04) | 0.30 (0.29 – 0.31) | 1.04 (1.02 – 1.05) | 1.05 (1.04 – 1.06) |
| Parental Education: Missing |  | 0.08 (0.06 – 0.10) | 0.06 (0.04 – 0.08) | 0.06 (0.04 – 0.08) | 0.06 (0.04 – 0.08) | 0.06 (0.04 – 0.07) | 0.06 (0.04 – 0.08) | 0.06 (0.04 – 0.08) |
| ADHD \* Girls *(Interaction)* |  |  |  |  |  |  | -0.04 (-0.07 – -0.00) |  |
| ADHD \* Parental Ed: High School *(Interaction)* |  |  |  |  |  |  |  | -0.13 (-0.18 – -0.08) |
| ADHD \* Parental Ed: Bachelor *(Interaction)* |  |  |  |  |  |  |  | -0.27 (-0.32 – -0.22) |
| ADHD \* Parental Ed: Master *(Interaction)* |  |  |  |  |  |  |  | -0.34 (-0.41 – -0.27) |
| ADHD \* Parental Ed: Missing*(Interaction)* |  |  |  |  |  |  |  | 0.06 (-0.03 – 0.15) |
| Early School Performance: Mathematics (z-score) |  |  |  |  |  | 0.62 (0.62 – 0.62) |  |  |
| Early School Performance: Reading (z-score) |  |  |  |  |  | 0.15 (0.15 – 0.16) |  |  |
| Number of Diagnoses: No other diagnoses |  |  |  | *Reference* |  |  |  |  |
| Number of Diagnoses: One other diagnosis |  |  |  | -0.19 (-0.20 – -0.18) |  |  |  |  |
| Number of Diagnoses: Two other diagnoses |  |  |  | -0.24 (-0.28 – -0.20) |  |  |  |  |
| Number of Diagnoses: Three or more other diagnoses |  |  |  | -0.19 (-0.28 – -0.10) |  |  |  |  |
| Anxiety Disorder (P74) |  |  |  |  | -0.20 (-0.23 – -0.17) |  |  |  |
| Somatization Disorder (P75) |  |  |  |  | -0.08 (-0.14 – -0.03) |  |  |  |
| Depressive Disorder (P76 |  |  |  |  | -0.12 (-0.14 – -0.10) |  |  |  |
| Suicide / Suicide Attempt (P77) |  |  |  |  | -0.16 (-0.22 – -0.11) |  |  |  |
| Phobia / Compulsive Disorder (P79) |  |  |  |  | -0.08 (-0.12 – -0.05) |  |  |  |
| Personality Disorder (P80) |  |  |  |  | -0.14 (-0.23 – -0.05) |  |  |  |
| PTSD (P82) |  |  |  |  | -0.29 (-0.37 – -0.22) |  |  |  |
| Anorexia Nervosa / Bulimia (P86) |  |  |  |  | 0.12 (0.04 – 0.19) |  |  |  |
| Other Psychological Disorders (P99) |  |  |  |  | -0.24 (-0.26 – -0.21) |  |  |  |
| Birth Year: 1997 |  | *Reference* | *Reference* | *Reference* | *Reference* | *Reference* | *Reference* | *Reference* |
| Birth Year: 1998 |  | 0.10 (0.09 – 0.11) | 0.10 (0.09 – 0.11) | 0.10 (0.09 – 0.11) | 0.10 (0.09 – 0.11) | 0.43 (0.42 – 0.44) | 0.10 (0.09 – 0.11) | 0.10 (0.09 – 0.11) |
| Birth Year: 1999 |  | -0.21 (-0.22 – -0.20) | -0.21 (-0.22 – -0.20) | -0.21 (-0.22 – -0.20) | -0.21 (-0.22 – -0.20) | 0.11 (0.10 – 0.11) | -0.21 (-0.22 – -0.20) | -0.21 (-0.22 – -0.20) |
| Birth Year: 2000 |  | -0.18 (-0.19 – -0.17) | -0.17 (-0.18 – -0.16) | -0.17 (-0.18 – -0.16) | -0.17 (-0.18 – -0.16) | 0.20 (0.19 – 0.21) | -0.17 (-0.18 – -0.16) | -0.17 (-0.18 – -0.16) |
| Birth Year: 2001 |  | -0.20 (-0.21 – -0.19) | -0.20 (-0.21 – -0.19) | -0.20 (-0.21 – -0.18) | -0.20 (-0.21 – -0.18) | 0.13 (0.12 – 0.14) | -0.20 (-0.21 – -0.19) | -0.20 (-0.21 – -0.19) |
| Birth Year: 2002 |  | -0.43 (-0.44 – -0.42) | -0.42 (-0.43 – -0.41) | -0.42 (-0.43 – -0.41) | -0.42 (-0.43 – -0.41) | -0.09 (-0.10 – -0.08) | -0.42 (-0.43 – -0.41) | -0.42 (-0.43 – -0.41) |
| Birth Month: January |  | *Reference* | *Reference* | *Reference* | *Reference* | *Reference* | *Reference* | *Reference* |
| Birth Month: February |  | -0.02 (-0.04 – -0.01) | -0.02 (-0.04 – -0.01) | -0.02 (-0.04 – -0.01) | -0.02 (-0.04 – -0.01) | 0.00 (-0.01 – 0.01) | -0.02 (-0.04 – -0.01) | -0.02 (-0.04 – -0.01) |
| Birth Month: March |  | -0.05 (-0.06 – -0.03) | -0.05 (-0.06 – -0.03) | -0.05 (-0.06 – -0.03) | -0.05 (-0.06 – -0.03) | 0.00 (-0.01 – 0.01) | -0.05 (-0.06 – -0.03) | -0.05 (-0.06 – -0.03) |
| Birth Month: April |  | -0.05 (-0.07 – -0.04) | -0.05 (-0.07 – -0.04) | -0.05 (-0.07 – -0.04) | -0.05 (-0.07 – -0.04) | 0.01 (0.00 – 0.02) | -0.05 (-0.07 – -0.04) | -0.05 (-0.07 – -0.04) |
| Birth Month: May |  | -0.08 (-0.09 – -0.06) | -0.07 (-0.09 – -0.06) | -0.07 (-0.09 – -0.06) | -0.07 (-0.09 – -0.06) | 0.02 (0.01 – 0.03) | -0.07 (-0.09 – -0.06) | -0.08 (-0.09 – -0.06) |
| Birth Month: June |  | -0.11 (-0.12 – -0.09) | -0.11 (-0.12 – -0.09) | -0.11 (-0.12 – -0.09) | -0.11 (-0.12 – -0.09) | 0.02 (0.01 – 0.03) | -0.11 (-0.12 – -0.09) | -0.11 (-0.12 – -0.09) |
| Birth Month: July |  | -0.13 (-0.15 – -0.12) | -0.13 (-0.14 – -0.11) | -0.13 (-0.14 – -0.11) | -0.13 (-0.14 – -0.11) | 0.02 (0.01 – 0.03) | -0.13 (-0.14 – -0.11) | -0.13 (-0.14 – -0.11) |
| Birth Month: August |  | -0.15 (-0.17 – -0.13) | -0.14 (-0.16 – -0.13) | -0.14 (-0.16 – -0.13) | -0.14 (-0.16 – -0.13) | 0.03 (0.02 – 0.04) | -0.14 (-0.16 – -0.13) | -0.14 (-0.16 – -0.13) |
| Birth Month: September |  | -0.18 (-0.19 – -0.16) | -0.17 (-0.19 – -0.16) | -0.17 (-0.18 – -0.15) | -0.17 (-0.18 – -0.15) | 0.03 (0.02 – 0.04) | -0.17 (-0.19 – -0.16) | -0.17 (-0.19 – -0.15) |
| Birth Month: October |  | -0.21 (-0.22 – -0.19) | -0.20 (-0.21 – -0.18) | -0.20 (-0.21 – -0.18) | -0.20 (-0.21 – -0.18) | 0.04 (0.03 – 0.05) | -0.20 (-0.21 – -0.18) | -0.20 (-0.21 – -0.18) |
| Birth Month: November |  | -0.23 (-0.25 – -0.22) | -0.22 (-0.24 – -0.21) | -0.22 (-0.24 – -0.21) | -0.22 (-0.24 – -0.20) | 0.04 (0.03 – 0.06) | -0.22 (-0.24 – -0.21) | -0.22 (-0.24 – -0.21) |
| Birth Month: December |  | -0.25 (-0.26 – -0.23) | -0.24 (-0.25 – -0.22) | -0.23 (-0.25 – -0.22) | -0.23 (-0.25 – -0.22) | 0.05 (0.03 – 0.06) | -0.24 (-0.25 – -0.22) | -0.24 (-0.25 – -0.22) |
| Parity: First-Born |  | *Reference* | *Reference* | *Reference* | *Reference* | *Reference* | *Reference* | *Reference* |
| Parity: Second-Born |  | -0.07 (-0.08 – -0.07) | -0.08 (-0.08 – -0.07) | -0.08 (-0.08 – -0.07) | -0.08 (-0.08 – -0.07) | -0.02 (-0.02 – -0.01) | -0.07 (-0.08 – -0.07) | -0.08 (-0.08 – -0.07) |
| Parity: Third-Born |  | -0.12 (-0.13 – -0.11) | -0.13 (-0.14 – -0.12) | -0.13 (-0.14 – -0.12) | -0.13 (-0.14 – -0.12) | -0.03 (-0.03 – -0.02) | -0.13 (-0.14 – -0.12) | -0.13 (-0.14 – -0.12) |
| Parity: Fourth-Born |  | -0.19 (-0.20 – -0.17) | -0.19 (-0.21 – -0.18) | -0.20 (-0.21 – -0.18) | -0.19 (-0.21 – -0.18) | -0.03 (-0.04 – -0.02) | -0.19 (-0.21 – -0.18) | -0.19 (-0.21 – -0.18) |
| Parity: Fifth-Born or later |  | -0.25 (-0.28 – -0.23) | -0.26 (-0.28 – -0.24) | -0.27 (-0.29 – -0.24) | -0.26 (-0.29 – -0.24) | -0.03 (-0.05 – -0.01) | -0.26 (-0.28 – -0.24) | -0.26 (-0.28 – -0.24) |
| Parity: Missing |  | -0.20 (-0.30 – -0.09) | -0.20 (-0.31 – -0.10) | -0.21 (-0.31 – -0.10) | -0.21 (-0.31 – -0.10) | -0.01 (-0.10 – 0.08) | -0.20 (-0.31 – -0.10) | -0.20 (-0.31 – -0.10) |
| (Intercept) | 0.03 (0.03 – 0.03) | -0.15 (-0.16 – -0.13) | -0.09 (-0.11 – -0.08) | -0.09 (-0.10 – -0.07) | -0.09 (-0.10 – -0.07) | -0.19 (-0.20 – -0.18) | -0.10 (-0.11 – -0.08) | -0.11 (-0.12 – -0.09) |
| Observations | 336923 | 336923 | 336923 | 336923 | 336923 | 310407 | 336923 | 336923 |
| R2 / R2 adjusted | 0.023 / 0.023 | 0.145 / 0.145 | 0.163 / 0.163 | 0.165 / 0.165 | 0.165 / 0.165 | 0.604 / 0.604 | 0.163 / 0.163 | 0.163 / 0.163 |
