# Supplementary material for: The ADHD deficit in school performance across sex and parental education: A prospective sibling‐comparison register study of 344,152 Norwegian adolescents
Source: JCPP Adv. 2022 Feb 12;2(1):e12064. doi: 10.1002/jcv2.12064 (PMC10242882; doi:10.1002/jcv2.12064)
Supplement: Supplementary file 1 — Supplementary Material S1 [file JCV2-2-e12064-s001.zip › Supporting Information/Supplementary Tables/Table S12.html]

Table S12: Regression Table – Reading, 8th grade (Bivariate and Adjusted Models)

| Dependent Variable: Test Score (z-score) | Bivariate: ADHD | Covariates Only | Fully Adjusted | + Number of Diagnoses | + Specific Diagnoses | + Early School Performance | Interaction w/ Sex | Interaction w/ Parental Education |
| Predictors | Estimates (95% CIs) | Estimates (95% CIs) | Estimates (95% CIs) | Estimates (95% CIs) | Estimates (95% CIs) | Estimates (95% CIs) | Estimates (95% CIs) | Estimates (95% CIs) |
| ADHD (P81) | -0.82 (-0.84 – -0.80) |  | -0.66 (-0.68 – -0.65) | -0.65 (-0.67 – -0.64) | -0.65 (-0.66 – -0.63) | -0.24 (-0.25 – -0.23) | -0.66 (-0.67 – -0.64) | -0.50 (-0.54 – -0.46) |
| Sex: Boys |  | *Reference* | *Reference* | *Reference* | *Reference* | *Reference* | *Reference* | *Reference* |
| Sex: Girls |  | 0.26 (0.25 – 0.26) | 0.24 (0.23 – 0.24) | 0.24 (0.23 – 0.24) | 0.24 (0.23 – 0.24) | 0.18 (0.17 – 0.18) | 0.24 (0.23 – 0.24) | 0.24 (0.23 – 0.24) |
| Parental Education: No High School |  | *Reference* | *Reference* | *Reference* | *Reference* | *Reference* | *Reference* | *Reference* |
| Parental Education: High School |  | 0.27 (0.26 – 0.28) | 0.26 (0.24 – 0.27) | 0.25 (0.24 – 0.27) | 0.25 (0.24 – 0.27) | 0.05 (0.04 – 0.06) | 0.26 (0.24 – 0.27) | 0.27 (0.25 – 0.28) |
| Parental Education: Bachelor's Degree (or equiv) |  | 0.65 (0.63 – 0.66) | 0.62 (0.61 – 0.63) | 0.62 (0.61 – 0.63) | 0.62 (0.61 – 0.63) | 0.17 (0.16 – 0.17) | 0.62 (0.61 – 0.63) | 0.63 (0.62 – 0.65) |
| Parental Education: Master's Degree (or equiv) |  | 0.99 (0.98 – 1.00) | 0.96 (0.94 – 0.97) | 0.95 (0.94 – 0.97) | 0.95 (0.94 – 0.97) | 0.28 (0.27 – 0.29) | 0.96 (0.94 – 0.97) | 0.97 (0.96 – 0.98) |
| Parental Education: Missing |  | 0.02 (0.00 – 0.04) | 0.01 (-0.01 – 0.03) | 0.01 (-0.01 – 0.02) | 0.01 (-0.01 – 0.02) | 0.05 (0.03 – 0.06) | 0.01 (-0.01 – 0.03) | 0.00 (-0.02 – 0.02) |
| ADHD \* Girls *(Interaction)* |  |  |  |  |  |  | -0.03 (-0.06 – 0.00) |  |
| ADHD \* Parental Ed: High School *(Interaction)* |  |  |  |  |  |  |  | -0.15 (-0.20 – -0.10) |
| ADHD \* Parental Ed: Bachelor *(Interaction)* |  |  |  |  |  |  |  | -0.26 (-0.31 – -0.21) |
| ADHD \* Parental Ed: Master *(Interaction)* |  |  |  |  |  |  |  | -0.32 (-0.39 – -0.25) |
| ADHD \* Parental Ed: Missing *(Interaction)* |  |  |  |  |  |  |  | 0.16 (0.07 – 0.25) |
| Early School Performance: Mathematics (z-score) |  |  |  |  |  | 0.21 (0.21 – 0.22) |  |  |
| Early School Performance: Reading (z-score) |  |  |  |  |  | 0.49 (0.48 – 0.49) |  |  |
| Number of Diagnoses: No other diagnoses |  |  |  | *Reference* |  |  |  |  |
| Number of Diagnoses: One other diagnosis |  |  |  | -0.10 (-0.12 – -0.09) |  |  |  |  |
| Number of Diagnoses: Two other diagnoses |  |  |  | -0.10 (-0.14 – -0.06) |  |  |  |  |
| Number of Diagnoses: Three or more other diagnoses |  |  |  | 0.01 (-0.08 – 0.10) |  |  |  |  |
| Anxiety Disorder (P74) |  |  |  |  | -0.08 (-0.11 – -0.05) |  |  |  |
| Somatization Disorder (P75) |  |  |  |  | -0.07 (-0.12 – -0.01) |  |  |  |
| Depressive Disorder (P76 |  |  |  |  | 0.00 (-0.02 – 0.02) |  |  |  |
| Suicide / Suicide Attempt (P77) |  |  |  |  | -0.11 (-0.16 – -0.05) |  |  |  |
| Phobia / Compulsive Disorder (P79) |  |  |  |  | -0.01 (-0.05 – 0.02) |  |  |  |
| Personality Disorder (P80) |  |  |  |  | -0.15 (-0.24 – -0.07) |  |  |  |
| PTSD (P82) |  |  |  |  | -0.20 (-0.27 – -0.13) |  |  |  |
| Anorexia Nervosa / Bulimia (P86) |  |  |  |  | 0.17 (0.10 – 0.24) |  |  |  |
| Other Psychological Disorders (P99) |  |  |  |  | -0.24 (-0.27 – -0.21) |  |  |  |
| Birth Year: 1997 |  | *Reference* | *Reference* | *Reference* | *Reference* | *Reference* | *Reference* | *Reference* |
| Birth Year: 1998 |  | 0.49 (0.48 – 0.50) | 0.49 (0.48 – 0.50) | 0.49 (0.48 – 0.50) | 0.49 (0.48 – 0.50) | 0.53 (0.52 – 0.53) | 0.49 (0.48 – 0.50) | 0.49 (0.48 – 0.50) |
| Birth Year: 1999 |  | 0.61 (0.60 – 0.62) | 0.62 (0.61 – 0.63) | 0.62 (0.61 – 0.63) | 0.62 (0.61 – 0.63) | 0.56 (0.55 – 0.57) | 0.62 (0.61 – 0.63) | 0.62 (0.61 – 0.63) |
| Birth Year: 2000 |  | 0.67 (0.66 – 0.68) | 0.67 (0.66 – 0.68) | 0.67 (0.66 – 0.68) | 0.67 (0.66 – 0.68) | 0.73 (0.72 – 0.73) | 0.67 (0.66 – 0.68) | 0.67 (0.66 – 0.68) |
| Birth Year: 2001 |  | 0.33 (0.32 – 0.34) | 0.34 (0.33 – 0.35) | 0.34 (0.33 – 0.35) | 0.34 (0.33 – 0.35) | 0.21 (0.20 – 0.22) | 0.34 (0.33 – 0.35) | 0.34 (0.33 – 0.35) |
| Birth Year: 2002 |  | 0.34 (0.32 – 0.35) | 0.34 (0.33 – 0.35) | 0.34 (0.33 – 0.35) | 0.34 (0.33 – 0.35) | 0.40 (0.39 – 0.41) | 0.34 (0.33 – 0.35) | 0.34 (0.33 – 0.35) |
| Birth Month: January |  | *Reference* | *Reference* | *Reference* | *Reference* | *Reference* | *Reference* | *Reference* |
| Birth Month: February |  | -0.03 (-0.04 – -0.01) | -0.03 (-0.04 – -0.01) | -0.03 (-0.04 – -0.01) | -0.03 (-0.04 – -0.01) | -0.00 (-0.01 – 0.01) | -0.03 (-0.04 – -0.01) | -0.03 (-0.04 – -0.01) |
| Birth Month: March |  | -0.05 (-0.06 – -0.03) | -0.05 (-0.06 – -0.03) | -0.05 (-0.06 – -0.03) | -0.05 (-0.06 – -0.03) | -0.00 (-0.01 – 0.01) | -0.05 (-0.06 – -0.03) | -0.05 (-0.06 – -0.03) |
| Birth Month: April |  | -0.06 (-0.07 – -0.04) | -0.06 (-0.07 – -0.04) | -0.06 (-0.07 – -0.04) | -0.06 (-0.07 – -0.04) | 0.00 (-0.01 – 0.02) | -0.06 (-0.07 – -0.04) | -0.06 (-0.07 – -0.04) |
| Birth Month: May |  | -0.09 (-0.11 – -0.08) | -0.09 (-0.11 – -0.08) | -0.09 (-0.11 – -0.08) | -0.09 (-0.11 – -0.08) | 0.00 (-0.01 – 0.01) | -0.09 (-0.11 – -0.08) | -0.09 (-0.11 – -0.08) |
| Birth Month: June |  | -0.12 (-0.14 – -0.11) | -0.12 (-0.13 – -0.10) | -0.12 (-0.13 – -0.10) | -0.12 (-0.13 – -0.10) | 0.00 (-0.01 – 0.02) | -0.12 (-0.13 – -0.10) | -0.12 (-0.13 – -0.10) |
| Birth Month: July |  | -0.15 (-0.16 – -0.13) | -0.14 (-0.16 – -0.13) | -0.14 (-0.15 – -0.13) | -0.14 (-0.16 – -0.13) | 0.00 (-0.01 – 0.01) | -0.14 (-0.16 – -0.13) | -0.14 (-0.16 – -0.13) |
| Birth Month: August |  | -0.17 (-0.19 – -0.16) | -0.17 (-0.18 – -0.15) | -0.16 (-0.18 – -0.15) | -0.16 (-0.18 – -0.15) | 0.00 (-0.01 – 0.02) | -0.17 (-0.18 – -0.15) | -0.17 (-0.18 – -0.15) |
| Birth Month: September |  | -0.20 (-0.22 – -0.19) | -0.20 (-0.21 – -0.18) | -0.19 (-0.21 – -0.18) | -0.19 (-0.21 – -0.18) | 0.00 (-0.01 – 0.01) | -0.20 (-0.21 – -0.18) | -0.20 (-0.21 – -0.18) |
| Birth Month: October |  | -0.22 (-0.23 – -0.20) | -0.21 (-0.22 – -0.19) | -0.21 (-0.22 – -0.19) | -0.21 (-0.22 – -0.19) | 0.01 (0.00 – 0.03) | -0.21 (-0.22 – -0.19) | -0.21 (-0.22 – -0.19) |
| Birth Month: November |  | -0.25 (-0.27 – -0.24) | -0.24 (-0.26 – -0.22) | -0.24 (-0.25 – -0.22) | -0.24 (-0.25 – -0.22) | 0.02 (0.00 – 0.03) | -0.24 (-0.25 – -0.22) | -0.24 (-0.25 – -0.22) |
| Birth Month: December |  | -0.27 (-0.29 – -0.26) | -0.26 (-0.28 – -0.25) | -0.26 (-0.28 – -0.25) | -0.26 (-0.28 – -0.25) | 0.01 (-0.00 – 0.02) | -0.26 (-0.28 – -0.25) | -0.26 (-0.28 – -0.25) |
| Parity: First-Born |  | *Reference* | *Reference* | *Reference* | *Reference* | *Reference* | *Reference* | *Reference* |
| Parity: Second-Born |  | -0.11 (-0.12 – -0.11) | -0.12 (-0.12 – -0.11) | -0.12 (-0.12 – -0.11) | -0.12 (-0.12 – -0.11) | -0.04 (-0.05 – -0.04) | -0.12 (-0.12 – -0.11) | -0.12 (-0.12 – -0.11) |
| Parity: Third-Born |  | -0.16 (-0.17 – -0.15) | -0.16 (-0.17 – -0.16) | -0.17 (-0.17 – -0.16) | -0.17 (-0.17 – -0.16) | -0.05 (-0.06 – -0.05) | -0.16 (-0.17 – -0.16) | -0.16 (-0.17 – -0.16) |
| Parity: Fourth-Born |  | -0.23 (-0.24 – -0.21) | -0.24 (-0.25 – -0.22) | -0.24 (-0.25 – -0.22) | -0.24 (-0.25 – -0.22) | -0.07 (-0.08 – -0.06) | -0.24 (-0.25 – -0.22) | -0.24 (-0.25 – -0.22) |
| Parity: Fifth-Born or later |  | -0.29 (-0.32 – -0.27) | -0.30 (-0.33 – -0.28) | -0.31 (-0.33 – -0.28) | -0.31 (-0.33 – -0.28) | -0.08 (-0.09 – -0.06) | -0.30 (-0.33 – -0.28) | -0.30 (-0.33 – -0.28) |
| Parity: Missing |  | -0.24 (-0.35 – -0.14) | -0.25 (-0.36 – -0.15) | -0.25 (-0.36 – -0.15) | -0.25 (-0.36 – -0.15) | -0.03 (-0.12 – 0.06) | -0.25 (-0.36 – -0.15) | -0.25 (-0.35 – -0.14) |
| (Intercept) | 0.03 (0.03 – 0.04) | -0.81 (-0.83 – -0.79) | -0.76 (-0.78 – -0.75) | -0.76 (-0.77 – -0.74) | -0.76 (-0.77 – -0.74) | -0.54 (-0.56 – -0.53) | -0.76 (-0.78 – -0.75) | -0.77 (-0.79 – -0.76) |
| Observations | 334583 | 334583 | 334583 | 334583 | 334583 | 308537 | 334583 | 334583 |
| R2 / R2 adjusted | 0.025 / 0.025 | 0.177 / 0.177 | 0.193 / 0.193 | 0.194 / 0.194 | 0.194 / 0.194 | 0.553 / 0.553 | 0.193 / 0.193 | 0.194 / 0.194 |
