# Supplementary material for: The ADHD deficit in school performance across sex and parental education: A prospective sibling‐comparison register study of 344,152 Norwegian adolescents
Source: JCPP Adv. 2022 Feb 12;2(1):e12064. doi: 10.1002/jcv2.12064 (PMC10242882; doi:10.1002/jcv2.12064)
Supplement: Supplementary file 1 — Supplementary Material S1 [file JCV2-2-e12064-s001.zip › Supporting Information/Supplementary Tables/Table S13.html]

Table S13: Regression Table – Reading, 8th grade (Sibling Models)

| Dependent Variable: Test Score (z-score) | Empty Sibling Model | ADHD Only | Covariates Only | Full Sibling Model | + Number of Diagnoses | + Specific Diagnoses | + Early School Performance | Interaction w/ Sex |
| Predictors | Estimates (95% CIs) | Estimates (95% CIs) | Estimates (95% CIs) | Estimates (95% CIs) | Estimates (95% CIs) | Estimates (95% CIs) | Estimates (95% CIs) | Estimates (95% CIs) |
| ADHD (P81) Within Families |  | -0.64 (-0.68 – -0.61) |  | -0.58 (-0.61 – -0.55) | -0.57 (-0.60 – -0.54) | -0.57 (-0.60 – -0.53) | -0.20 (-0.23 – -0.17) | -0.57 (-0.60 – -0.53) |
| ADHD (P81) Between Families |  | -0.39 (-0.45 – -0.33) |  | -0.39 (-0.44 – -0.34) | -0.38 (-0.43 – -0.33) | -0.38 (-0.43 – -0.32) | -0.11 (-0.15 – -0.07) | -0.39 (-0.44 – -0.33) |
| Sex: Boys |  |  | *Reference* | *Reference* | *Reference* | *Reference* | *Reference* | *Reference* |
| Sex: Girls |  |  | 0.23 (0.22 – 0.24) | 0.21 (0.20 – 0.22) | 0.21 (0.20 – 0.22) | 0.21 (0.20 – 0.22) | 0.17 (0.16 – 0.18) | 0.21 (0.20 – 0.22) |
| ADHD \* Girls *(Interaction)* |  |  |  |  |  |  |  | -0.04 (-0.10 – 0.02) |
| Early School Performance: Mathematics (z-score) |  |  |  |  |  |  | 0.23 (0.22 – 0.23) |  |
| Early School Performance: Reading (z-score) |  |  |  |  |  |  | 0.50 (0.49 – 0.50) |  |
| Number of Diagnoses: No other diagnoses |  |  |  |  | *Reference* |  |  |  |
| Number of Diagnoses: One other diagnosis |  |  |  |  | -0.13 (-0.15 – -0.11) |  |  |  |
| Number of Diagnoses: Two other diagnoses |  |  |  |  | -0.09 (-0.15 – -0.02) |  |  |  |
| Number of Diagnoses: Three or more other diagnoses |  |  |  |  | -0.09 (-0.24 – 0.06) |  |  |  |
| Anxiety Disorder (P74) |  |  |  |  |  | -0.12 (-0.17 – -0.07) |  |  |
| Somatization Disorder (P75) |  |  |  |  |  | -0.08 (-0.17 – 0.01) |  |  |
| Depressive Disorder (P76) |  |  |  |  |  | -0.02 (-0.06 – 0.01) |  |  |
| Suicide / Suicide Attempt (P77) |  |  |  |  |  | -0.08 (-0.18 – 0.01) |  |  |
| Phobia / Compulsive Disorder (P79) |  |  |  |  |  | -0.02 (-0.07 – 0.04) |  |  |
| Personality Disorder (P80) |  |  |  |  |  | -0.09 (-0.23 – 0.05) |  |  |
| PTSD (P82) |  |  |  |  |  | -0.18 (-0.30 – -0.06) |  |  |
| Anorexia Nervosa / Bulimia (P86) |  |  |  |  |  | 0.22 (0.11 – 0.32) |  |  |
| Other Psychological Disorders (P99) |  |  |  |  |  | -0.27 (-0.31 – -0.22) |  |  |
| Birth Year: 1997 |  |  | *Reference* | *Reference* | *Reference* | *Reference* | *Reference* | *Reference* |
| Birth Year: 1998 |  |  | 0.52 (0.50 – 0.53) | 0.52 (0.50 – 0.53) | 0.52 (0.50 – 0.53) | 0.51 (0.50 – 0.53) | 0.54 (0.53 – 0.56) | 0.52 (0.50 – 0.53) |
| Birth Year: 1999 |  |  | 0.66 (0.65 – 0.68) | 0.66 (0.65 – 0.68) | 0.66 (0.65 – 0.68) | 0.66 (0.65 – 0.68) | 0.58 (0.57 – 0.59) | 0.66 (0.65 – 0.68) |
| Birth Year: 2000 |  |  | 0.75 (0.73 – 0.77) | 0.75 (0.73 – 0.77) | 0.75 (0.74 – 0.77) | 0.75 (0.74 – 0.77) | 0.77 (0.76 – 0.78) | 0.75 (0.73 – 0.77) |
| Birth Year: 2001 |  |  | 0.42 (0.41 – 0.44) | 0.43 (0.41 – 0.44) | 0.43 (0.41 – 0.44) | 0.43 (0.41 – 0.44) | 0.23 (0.21 – 0.24) | 0.43 (0.41 – 0.44) |
| Birth Year: 2002 |  |  | 0.43 (0.41 – 0.45) | 0.43 (0.41 – 0.45) | 0.43 (0.41 – 0.45) | 0.43 (0.41 – 0.45) | 0.42 (0.41 – 0.43) | 0.43 (0.41 – 0.45) |
| Birth Month: January |  |  | *Reference* | *Reference* | *Reference* | *Reference* | *Reference* | *Reference* |
| Birth Month: February |  |  | -0.03 (-0.06 – -0.01) | -0.03 (-0.06 – -0.01) | -0.03 (-0.05 – -0.01) | -0.03 (-0.05 – -0.01) | -0.01 (-0.03 – 0.01) | -0.03 (-0.06 – -0.01) |
| Birth Month: March |  |  | -0.04 (-0.07 – -0.02) | -0.04 (-0.07 – -0.02) | -0.04 (-0.07 – -0.02) | -0.04 (-0.07 – -0.02) | -0.01 (-0.02 – 0.01) | -0.04 (-0.07 – -0.02) |
| Birth Month: April |  |  | -0.05 (-0.08 – -0.03) | -0.05 (-0.08 – -0.03) | -0.05 (-0.08 – -0.03) | -0.05 (-0.08 – -0.03) | 0.00 (-0.01 – 0.02) | -0.05 (-0.08 – -0.03) |
| Birth Month: May |  |  | -0.09 (-0.11 – -0.07) | -0.09 (-0.11 – -0.07) | -0.09 (-0.11 – -0.07) | -0.09 (-0.11 – -0.07) | 0.00 (-0.01 – 0.02) | -0.09 (-0.11 – -0.07) |
| Birth Month: June |  |  | -0.11 (-0.13 – -0.08) | -0.10 (-0.12 – -0.08) | -0.10 (-0.12 – -0.08) | -0.10 (-0.12 – -0.08) | 0.01 (-0.01 – 0.03) | -0.10 (-0.12 – -0.08) |
| Birth Month: July |  |  | -0.14 (-0.16 – -0.12) | -0.13 (-0.16 – -0.11) | -0.13 (-0.16 – -0.11) | -0.13 (-0.16 – -0.11) | 0.01 (-0.01 – 0.03) | -0.13 (-0.16 – -0.11) |
| Birth Month: August |  |  | -0.16 (-0.18 – -0.14) | -0.15 (-0.17 – -0.13) | -0.15 (-0.17 – -0.13) | -0.15 (-0.17 – -0.13) | 0.02 (-0.00 – 0.03) | -0.15 (-0.17 – -0.13) |
| Birth Month: September |  |  | -0.20 (-0.22 – -0.17) | -0.19 (-0.21 – -0.17) | -0.19 (-0.21 – -0.17) | -0.19 (-0.21 – -0.17) | 0.01 (-0.01 – 0.03) | -0.19 (-0.21 – -0.17) |
| Birth Month: October |  |  | -0.21 (-0.23 – -0.18) | -0.20 (-0.22 – -0.18) | -0.20 (-0.22 – -0.18) | -0.20 (-0.22 – -0.18) | 0.02 (0.00 – 0.04) | -0.20 (-0.22 – -0.18) |
| Birth Month: November |  |  | -0.25 (-0.27 – -0.23) | -0.24 (-0.26 – -0.21) | -0.24 (-0.26 – -0.21) | -0.24 (-0.26 – -0.21) | 0.02 (0.00 – 0.04) | -0.24 (-0.26 – -0.21) |
| Birth Month: December |  |  | -0.26 (-0.29 – -0.24) | -0.25 (-0.28 – -0.23) | -0.25 (-0.28 – -0.23) | -0.25 (-0.28 – -0.23) | 0.02 (0.00 – 0.04) | -0.25 (-0.28 – -0.23) |
| Parity: First-Born |  |  | *Reference* | *Reference* | *Reference* | *Reference* | *Reference* | *Reference* |
| Parity: Second-Born |  |  | -0.16 (-0.17 – -0.15) | -0.16 (-0.17 – -0.14) | -0.16 (-0.17 – -0.14) | -0.16 (-0.17 – -0.15) | -0.04 (-0.05 – -0.03) | -0.16 (-0.17 – -0.14) |
| Parity: Third-Born |  |  | -0.28 (-0.30 – -0.26) | -0.28 (-0.30 – -0.26) | -0.28 (-0.30 – -0.26) | -0.28 (-0.30 – -0.26) | -0.08 (-0.09 – -0.06) | -0.28 (-0.30 – -0.26) |
| Parity: Fourth-Born or later |  |  | -0.42 (-0.45 – -0.40) | -0.42 (-0.44 – -0.39) | -0.42 (-0.44 – -0.39) | -0.42 (-0.44 – -0.39) | -0.11 (-0.13 – -0.09) | -0.42 (-0.44 – -0.39) |
| Parity: Fifth-Born |  |  | -0.55 (-0.58 – -0.51) | -0.54 (-0.58 – -0.51) | -0.54 (-0.58 – -0.51) | -0.54 (-0.58 – -0.51) | -0.15 (-0.17 – -0.12) | -0.54 (-0.58 – -0.51) |
| (Intercept) | 0.04 (0.03 – 0.04) | 0.07 (0.07 – 0.08) | -0.27 (-0.29 – -0.25) | -0.24 (-0.26 – -0.22) | -0.23 (-0.25 – -0.21) | -0.23 (-0.25 – -0.21) | -0.42 (-0.43 – -0.40) | -0.24 (-0.26 – -0.22) |
| Random Effects | | | | | | | | |
| σ2 | 0.64 | 0.63 | 0.55 | 0.54 | 0.54 | 0.54 | 0.37 | 0.54 || τ00 | 0.36 parents | 0.34 parents | 0.35 parents | 0.34 parents | 0.34 parents | 0.34 parents | 0.05 parents | 0.34 parents || ICC | 0.36 | 0.35 | 0.39 | 0.38 | 0.38 | 0.38 | 0.11 | 0.38 || N | 69550 parents | 69550 parents | 69550 parents | 69550 parents | 69550 parents | 69550 parents | 68328 parents | 69550 parents || Observations | 141336 | 141336 | 141336 | 141336 | 141336 | 141336 | 131307 | 141336 |
| Marginal R2 / Conditional R2 | 0.000 / 0.359 | 0.025 / 0.370 | 0.088 / 0.443 | 0.110 / 0.453 | 0.111 / 0.453 | 0.111 / 0.453 | 0.548 / 0.599 | 0.110 / 0.453 |
