# Supplementary material for: The ADHD deficit in school performance across sex and parental education: A prospective sibling‐comparison register study of 344,152 Norwegian adolescents
Source: JCPP Adv. 2022 Feb 12;2(1):e12064. doi: 10.1002/jcv2.12064 (PMC10242882; doi:10.1002/jcv2.12064)
Supplement: Supplementary file 1 — Supplementary Material S1 [file JCV2-2-e12064-s001.zip › Supporting Information/Supplementary Tables/Table S14.html]

Table S14: Regression Table – Mathematics, 9th grade (Bivariate and Adjusted Models)

| Dependent Variable: Test Score (z-score) | Bivariate: ADHD | Covariates Only | Fully Adjusted | + Number of Diagnoses | + Specific Diagnoses | + Early School Performance | Interaction w/ Sex | Interaction w/ Parental Education |
| Predictors | Estimates (95% CIs) | Estimates (95% CIs) | Estimates (95% CIs) | Estimates (95% CIs) | Estimates (95% CIs) | Estimates (95% CIs) | Estimates (95% CIs) | Estimates (95% CIs) |
| ADHD (P81) | -0.84 (-0.85 – -0.82) |  | -0.74 (-0.76 – -0.73) | -0.72 (-0.73 – -0.70) | -0.72 (-0.73 – -0.70) | -0.30 (-0.31 – -0.28) | -0.73 (-0.75 – -0.71) | -0.61 (-0.65 – -0.57) |
| Sex: Boys |  | *Reference* | *Reference* | *Reference* | *Reference* | *Reference* | *Reference* | *Reference* |
| Sex: Girls |  | -0.11 (-0.12 – -0.11) | -0.14 (-0.14 – -0.13) | -0.13 (-0.13 – -0.12) | -0.13 (-0.14 – -0.12) | -0.08 (-0.08 – -0.07) | -0.13 (-0.14 – -0.13) | -0.14 (-0.14 – -0.13) |
| Parental Education: No High School |  | *Reference* | *Reference* | *Reference* | *Reference* | *Reference* | *Reference* | *Reference* |
| Parental Education: High School |  | 0.32 (0.31 – 0.34) | 0.31 (0.30 – 0.32) | 0.31 (0.30 – 0.32) | 0.31 (0.30 – 0.32) | 0.10 (0.09 – 0.11) | 0.31 (0.30 – 0.32) | 0.32 (0.31 – 0.33) |
| Parental Education: Bachelor's Degree (or equiv) |  | 0.71 (0.70 – 0.72) | 0.68 (0.67 – 0.69) | 0.68 (0.67 – 0.69) | 0.68 (0.67 – 0.69) | 0.21 (0.20 – 0.22) | 0.68 (0.67 – 0.69) | 0.69 (0.68 – 0.70) |
| Parental Education: Master's Degree (or equiv) |  | 1.05 (1.03 – 1.06) | 1.01 (1.00 – 1.02) | 1.00 (0.99 – 1.02) | 1.00 (0.99 – 1.02) | 0.31 (0.30 – 0.32) | 1.01 (1.00 – 1.02) | 1.02 (1.01 – 1.03) |
| Parental Education: Missing |  | 0.08 (0.06 – 0.10) | 0.06 (0.04 – 0.08) | 0.06 (0.04 – 0.08) | 0.06 (0.04 – 0.08) | 0.06 (0.04 – 0.07) | 0.06 (0.04 – 0.08) | 0.06 (0.04 – 0.08) |
| ADHD \* Girls *(Interaction)* |  |  |  |  |  |  | -0.05 (-0.09 – -0.02) |  |
| ADHD \* Parental Ed: High School *(Interaction)* |  |  |  |  |  |  |  | -0.12 (-0.17 – -0.07) |
| ADHD \* Parental Ed: Bachelor *(Interaction)* |  |  |  |  |  |  |  | -0.20 (-0.25 – -0.15) |
| ADHD \* Parental Ed: Master *(Interaction)* |  |  |  |  |  |  |  | -0.27 (-0.34 – -0.19) |
| ADHD \* Parental Ed: Missing *(Interaction)* |  |  |  |  |  |  |  | 0.09 (0.00 – 0.19) |
| Early School Performance: Mathematics (z-score) |  |  |  |  |  | 0.58 (0.58 – 0.58) |  |  |
| Early School Performance: Reading (z-score) |  |  |  |  |  | 0.15 (0.14 – 0.15) |  |  |
| Number of Diagnoses: No other diagnoses |  |  |  | *Reference* |  |  |  |  |
| Number of Diagnoses: One other diagnosis |  |  |  | -0.24 (-0.25 – -0.23) |  |  |  |  |
| Number of Diagnoses: Two other diagnoses |  |  |  | -0.34 (-0.38 – -0.30) |  |  |  |  |
| Number of Diagnoses: Three or more other diagnoses |  |  |  | -0.27 (-0.36 – -0.18) |  |  |  |  |
| Anxiety Disorder (P74) |  |  |  |  | -0.25 (-0.28 – -0.22) |  |  |  |
| Somatization Disorder (P75) |  |  |  |  | -0.10 (-0.15 – -0.04) |  |  |  |
| Depressive Disorder (P76 |  |  |  |  | -0.17 (-0.19 – -0.15) |  |  |  |
| Suicide / Suicide Attempt (P77) |  |  |  |  | -0.25 (-0.31 – -0.19) |  |  |  |
| Phobia / Compulsive Disorder (P79) |  |  |  |  | -0.08 (-0.11 – -0.04) |  |  |  |
| Personality Disorder (P80) |  |  |  |  | -0.20 (-0.28 – -0.11) |  |  |  |
| PTSD (P82) |  |  |  |  | -0.37 (-0.44 – -0.30) |  |  |  |
| Anorexia Nervosa / Bulimia (P86) |  |  |  |  | 0.10 (0.02 – 0.17) |  |  |  |
| Other Psychological Disorders (P99) |  |  |  |  | -0.31 (-0.33 – -0.28) |  |  |  |
| Birth Year: 1997 |  | *Reference* | *Reference* | *Reference* | *Reference* | *Reference* | *Reference* | *Reference* |
| Birth Year: 1998 |  | -0.23 (-0.24 – -0.22) | -0.23 (-0.24 – -0.22) | -0.23 (-0.24 – -0.22) | -0.23 (-0.24 – -0.22) | 0.08 (0.07 – 0.09) | -0.23 (-0.24 – -0.22) | -0.23 (-0.24 – -0.22) |
| Birth Year: 1999 |  | -0.23 (-0.24 – -0.22) | -0.23 (-0.24 – -0.22) | -0.23 (-0.24 – -0.21) | -0.23 (-0.24 – -0.21) | 0.08 (0.07 – 0.09) | -0.23 (-0.24 – -0.22) | -0.23 (-0.24 – -0.22) |
| Birth Year: 2000 |  | -0.21 (-0.22 – -0.20) | -0.20 (-0.21 – -0.19) | -0.20 (-0.21 – -0.19) | -0.20 (-0.21 – -0.19) | 0.16 (0.16 – 0.17) | -0.20 (-0.21 – -0.19) | -0.20 (-0.21 – -0.19) |
| Birth Year: 2001 |  | -0.47 (-0.48 – -0.46) | -0.47 (-0.48 – -0.46) | -0.46 (-0.48 – -0.45) | -0.46 (-0.48 – -0.45) | -0.17 (-0.18 – -0.16) | -0.47 (-0.48 – -0.46) | -0.47 (-0.48 – -0.46) |
| Birth Year: 2002 |  | -0.59 (-0.60 – -0.58) | -0.59 (-0.60 – -0.57) | -0.58 (-0.59 – -0.57) | -0.58 (-0.59 – -0.57) | -0.28 (-0.28 – -0.27) | -0.59 (-0.60 – -0.57) | -0.59 (-0.60 – -0.57) |
| Birth Month: January |  | *Reference* | *Reference* | *Reference* | *Reference* | *Reference* | *Reference* | *Reference* |
| Birth Month: February |  | -0.02 (-0.04 – -0.01) | -0.02 (-0.04 – -0.01) | -0.02 (-0.04 – -0.01) | -0.02 (-0.04 – -0.01) | 0.00 (-0.01 – 0.02) | -0.02 (-0.04 – -0.01) | -0.02 (-0.04 – -0.01) |
| Birth Month: March |  | -0.05 (-0.06 – -0.03) | -0.04 (-0.06 – -0.03) | -0.04 (-0.06 – -0.03) | -0.04 (-0.06 – -0.03) | 0.00 (-0.01 – 0.01) | -0.04 (-0.06 – -0.03) | -0.04 (-0.06 – -0.03) |
| Birth Month: April |  | -0.04 (-0.06 – -0.03) | -0.04 (-0.06 – -0.03) | -0.04 (-0.05 – -0.03) | -0.04 (-0.05 – -0.03) | 0.02 (0.01 – 0.03) | -0.04 (-0.06 – -0.03) | -0.04 (-0.06 – -0.03) |
| Birth Month: May |  | -0.07 (-0.08 – -0.05) | -0.07 (-0.08 – -0.05) | -0.07 (-0.08 – -0.05) | -0.07 (-0.08 – -0.05) | 0.02 (0.01 – 0.03) | -0.07 (-0.08 – -0.05) | -0.07 (-0.08 – -0.05) |
| Birth Month: June |  | -0.10 (-0.11 – -0.08) | -0.09 (-0.11 – -0.08) | -0.09 (-0.11 – -0.08) | -0.09 (-0.11 – -0.08) | 0.03 (0.02 – 0.04) | -0.09 (-0.11 – -0.08) | -0.09 (-0.11 – -0.08) |
| Birth Month: July |  | -0.11 (-0.13 – -0.10) | -0.11 (-0.12 – -0.09) | -0.11 (-0.12 – -0.09) | -0.11 (-0.12 – -0.09) | 0.03 (0.02 – 0.04) | -0.11 (-0.12 – -0.09) | -0.11 (-0.12 – -0.09) |
| Birth Month: August |  | -0.13 (-0.15 – -0.12) | -0.13 (-0.14 – -0.11) | -0.12 (-0.14 – -0.11) | -0.12 (-0.14 – -0.11) | 0.04 (0.03 – 0.05) | -0.13 (-0.14 – -0.11) | -0.13 (-0.14 – -0.11) |
| Birth Month: September |  | -0.16 (-0.17 – -0.14) | -0.15 (-0.16 – -0.13) | -0.15 (-0.16 – -0.13) | -0.15 (-0.16 – -0.13) | 0.05 (0.04 – 0.06) | -0.15 (-0.16 – -0.13) | -0.15 (-0.16 – -0.13) |
| Birth Month: October |  | -0.18 (-0.20 – -0.16) | -0.17 (-0.19 – -0.16) | -0.17 (-0.18 – -0.15) | -0.17 (-0.18 – -0.15) | 0.05 (0.04 – 0.07) | -0.17 (-0.19 – -0.16) | -0.17 (-0.19 – -0.16) |
| Birth Month: November |  | -0.21 (-0.22 – -0.19) | -0.19 (-0.21 – -0.18) | -0.19 (-0.21 – -0.18) | -0.19 (-0.21 – -0.18) | 0.06 (0.05 – 0.07) | -0.19 (-0.21 – -0.18) | -0.19 (-0.21 – -0.18) |
| Birth Month: December |  | -0.21 (-0.23 – -0.20) | -0.20 (-0.22 – -0.18) | -0.20 (-0.21 – -0.18) | -0.20 (-0.21 – -0.18) | 0.07 (0.06 – 0.08) | -0.20 (-0.21 – -0.18) | -0.20 (-0.22 – -0.18) |
| Parity: First-Born |  | *Reference* | *Reference* | *Reference* | *Reference* | *Reference* | *Reference* | *Reference* |
| Parity: Second-Born |  | -0.07 (-0.08 – -0.06) | -0.07 (-0.08 – -0.06) | -0.07 (-0.08 – -0.07) | -0.07 (-0.08 – -0.07) | -0.02 (-0.02 – -0.01) | -0.07 (-0.08 – -0.06) | -0.07 (-0.08 – -0.07) |
| Parity: Third-Born |  | -0.11 (-0.12 – -0.10) | -0.12 (-0.13 – -0.11) | -0.12 (-0.13 – -0.11) | -0.12 (-0.13 – -0.11) | -0.02 (-0.03 – -0.01) | -0.12 (-0.13 – -0.11) | -0.12 (-0.13 – -0.11) |
| Parity: Fourth-Born |  | -0.18 (-0.19 – -0.16) | -0.18 (-0.20 – -0.17) | -0.19 (-0.20 – -0.17) | -0.19 (-0.20 – -0.17) | -0.03 (-0.04 – -0.02) | -0.18 (-0.20 – -0.17) | -0.18 (-0.20 – -0.17) |
| Parity: Fifth-Born or later |  | -0.25 (-0.27 – -0.22) | -0.26 (-0.28 – -0.23) | -0.26 (-0.29 – -0.24) | -0.26 (-0.29 – -0.24) | -0.04 (-0.05 – -0.02) | -0.26 (-0.28 – -0.23) | -0.26 (-0.28 – -0.23) |
| Parity: Missing |  | -0.16 (-0.26 – -0.05) | -0.17 (-0.27 – -0.06) | -0.17 (-0.28 – -0.07) | -0.17 (-0.28 – -0.06) | 0.04 (-0.05 – 0.13) | -0.17 (-0.27 – -0.06) | -0.16 (-0.27 – -0.06) |
| (Intercept) | 0.03 (0.03 – 0.04) | -0.04 (-0.06 – -0.03) | 0.01 (-0.00 – 0.03) | 0.02 (0.01 – 0.04) | 0.02 (0.01 – 0.04) | -0.08 (-0.09 – -0.06) | 0.01 (-0.01 – 0.03) | 0.00 (-0.01 – 0.02) |
| Observations | 337712 | 337712 | 337712 | 337712 | 337712 | 310577 | 337712 | 337712 |
| R2 / R2 adjusted | 0.026 / 0.026 | 0.145 / 0.145 | 0.165 / 0.165 | 0.169 / 0.169 | 0.169 / 0.169 | 0.562 / 0.562 | 0.165 / 0.165 | 0.166 / 0.166 |
