# Supplementary material for: The ADHD deficit in school performance across sex and parental education: A prospective sibling‐comparison register study of 344,152 Norwegian adolescents
Source: JCPP Adv. 2022 Feb 12;2(1):e12064. doi: 10.1002/jcv2.12064 (PMC10242882; doi:10.1002/jcv2.12064)
Supplement: Supplementary file 1 — Supplementary Material S1 [file JCV2-2-e12064-s001.zip › Supporting Information/Supplementary Tables/Table S15.html]

Table S15: Regression Table – Mathematics, 9th grade (Sibling Models)

| Dependent Variable: Test Score (z-score) | Empty Sibling Model | ADHD Only | Covariates Only | Full Sibling Model | + Number of Diagnoses | + Specific Diagnoses | + Early School Performance | Interaction w/ Sex |
| Predictors | Estimates (95% CIs) | Estimates (95% CIs) | Estimates (95% CIs) | Estimates (95% CIs) | Estimates (95% CIs) | Estimates (95% CIs) | Estimates (95% CIs) | Estimates (95% CIs) |
| ADHD (P81) Within Families |  | -0.57 (-0.60 – -0.53) |  | -0.57 (-0.60 – -0.54) | -0.56 (-0.59 – -0.52) | -0.55 (-0.59 – -0.52) | -0.19 (-0.22 – -0.17) | -0.55 (-0.59 – -0.52) |
| ADHD (P81) Between Families |  | -0.48 (-0.54 – -0.42) |  | -0.48 (-0.54 – -0.43) | -0.46 (-0.52 – -0.41) | -0.46 (-0.52 – -0.41) | -0.18 (-0.22 – -0.14) | -0.48 (-0.54 – -0.43) |
| Sex: Boys |  |  | *Reference* | *Reference* | *Reference* | *Reference* | *Reference* | *Reference* |
| Sex: Girls |  |  | -0.12 (-0.13 – -0.11) | -0.14 (-0.15 – -0.13) | -0.14 (-0.14 – -0.13) | -0.14 (-0.15 – -0.13) | -0.08 (-0.08 – -0.07) | -0.14 (-0.15 – -0.13) |
| ADHD \* Girls *(Interaction)* |  |  |  |  |  |  |  | -0.07 (-0.12 – -0.01) |
| Early School Performance: Mathematics (z-score) |  |  |  |  |  |  | 0.58 (0.58 – 0.58) |  |
| Early School Performance: Reading (z-score) |  |  |  |  |  |  | 0.16 (0.16 – 0.17) |  |
| Number of Diagnoses: No other diagnoses |  |  |  |  | *Reference* |  |  |  |
| Number of Diagnoses: One other diagnosis |  |  |  |  | -0.23 (-0.25 – -0.21) |  |  |  |
| Number of Diagnoses: Two other diagnoses |  |  |  |  | -0.29 (-0.35 – -0.23) |  |  |  |
| Number of Diagnoses: Three or more other diagnoses |  |  |  |  | -0.34 (-0.50 – -0.19) |  |  |  |
| Anxiety Disorder (P74) |  |  |  |  |  | -0.21 (-0.26 – -0.16) |  |  |
| Somatization Disorder (P75) |  |  |  |  |  | -0.18 (-0.26 – -0.09) |  |  |
| Depressive Disorder (P76) |  |  |  |  |  | -0.18 (-0.21 – -0.14) |  |  |
| Suicide / Suicide Attempt (P77) |  |  |  |  |  | -0.23 (-0.33 – -0.13) |  |  |
| Phobia / Compulsive Disorder (P79) |  |  |  |  |  | -0.07 (-0.13 – -0.02) |  |  |
| Personality Disorder (P80) |  |  |  |  |  | -0.16 (-0.29 – -0.02) |  |  |
| PTSD (P82) |  |  |  |  |  | -0.28 (-0.40 – -0.16) |  |  |
| Anorexia Nervosa / Bulimia (P86) |  |  |  |  |  | 0.13 (0.02 – 0.24) |  |  |
| Other Psychological Disorders (P99) |  |  |  |  |  | -0.30 (-0.35 – -0.26) |  |  |
| Birth Year: 1997 |  |  | *Reference* | *Reference* | *Reference* | *Reference* | *Reference* | *Reference* |
| Birth Year: 1998 |  |  | -0.22 (-0.23 – -0.20) | -0.22 (-0.23 – -0.20) | -0.22 (-0.23 – -0.20) | -0.22 (-0.23 – -0.20) | 0.08 (0.07 – 0.09) | -0.22 (-0.23 – -0.20) |
| Birth Year: 1999 |  |  | -0.19 (-0.21 – -0.18) | -0.19 (-0.21 – -0.18) | -0.19 (-0.21 – -0.18) | -0.19 (-0.21 – -0.18) | 0.08 (0.07 – 0.09) | -0.19 (-0.21 – -0.18) |
| Birth Year: 2000 |  |  | -0.14 (-0.15 – -0.12) | -0.14 (-0.15 – -0.12) | -0.13 (-0.15 – -0.12) | -0.13 (-0.15 – -0.12) | 0.18 (0.17 – 0.19) | -0.14 (-0.15 – -0.12) |
| Birth Year: 2001 |  |  | -0.40 (-0.42 – -0.39) | -0.40 (-0.42 – -0.39) | -0.40 (-0.42 – -0.38) | -0.40 (-0.42 – -0.38) | -0.18 (-0.20 – -0.17) | -0.40 (-0.42 – -0.39) |
| Birth Year: 2002 |  |  | -0.51 (-0.53 – -0.49) | -0.51 (-0.53 – -0.49) | -0.51 (-0.52 – -0.49) | -0.51 (-0.52 – -0.49) | -0.28 (-0.29 – -0.26) | -0.51 (-0.53 – -0.49) |
| Birth Month: January |  |  | *Reference* | *Reference* | *Reference* | *Reference* | *Reference* | *Reference* |
| Birth Month: February |  |  | -0.02 (-0.05 – -0.00) | -0.02 (-0.05 – -0.00) | -0.02 (-0.05 – -0.00) | -0.02 (-0.05 – -0.00) | -0.00 (-0.02 – 0.02) | -0.02 (-0.05 – -0.00) |
| Birth Month: March |  |  | -0.05 (-0.07 – -0.03) | -0.05 (-0.07 – -0.03) | -0.05 (-0.07 – -0.03) | -0.05 (-0.07 – -0.03) | -0.01 (-0.02 – 0.01) | -0.05 (-0.07 – -0.03) |
| Birth Month: April |  |  | -0.05 (-0.07 – -0.03) | -0.05 (-0.07 – -0.03) | -0.05 (-0.07 – -0.03) | -0.05 (-0.07 – -0.03) | 0.01 (-0.00 – 0.03) | -0.05 (-0.07 – -0.03) |
| Birth Month: May |  |  | -0.07 (-0.09 – -0.04) | -0.06 (-0.09 – -0.04) | -0.06 (-0.09 – -0.04) | -0.06 (-0.09 – -0.04) | 0.02 (0.00 – 0.04) | -0.06 (-0.09 – -0.04) |
| Birth Month: June |  |  | -0.09 (-0.11 – -0.07) | -0.08 (-0.11 – -0.06) | -0.08 (-0.10 – -0.06) | -0.08 (-0.10 – -0.06) | 0.03 (0.01 – 0.04) | -0.08 (-0.11 – -0.06) |
| Birth Month: July |  |  | -0.12 (-0.14 – -0.10) | -0.11 (-0.14 – -0.09) | -0.11 (-0.13 – -0.09) | -0.11 (-0.13 – -0.09) | 0.03 (0.02 – 0.05) | -0.11 (-0.13 – -0.09) |
| Birth Month: August |  |  | -0.12 (-0.14 – -0.10) | -0.11 (-0.13 – -0.09) | -0.11 (-0.13 – -0.09) | -0.11 (-0.13 – -0.09) | 0.05 (0.03 – 0.07) | -0.11 (-0.13 – -0.09) |
| Birth Month: September |  |  | -0.17 (-0.19 – -0.14) | -0.16 (-0.18 – -0.14) | -0.16 (-0.18 – -0.14) | -0.16 (-0.18 – -0.14) | 0.04 (0.02 – 0.06) | -0.16 (-0.18 – -0.14) |
| Birth Month: October |  |  | -0.17 (-0.19 – -0.15) | -0.16 (-0.18 – -0.14) | -0.16 (-0.18 – -0.14) | -0.16 (-0.18 – -0.14) | 0.06 (0.04 – 0.07) | -0.16 (-0.18 – -0.14) |
| Birth Month: November |  |  | -0.20 (-0.22 – -0.18) | -0.19 (-0.21 – -0.16) | -0.19 (-0.21 – -0.16) | -0.19 (-0.21 – -0.16) | 0.07 (0.05 – 0.08) | -0.19 (-0.21 – -0.16) |
| Birth Month: December |  |  | -0.21 (-0.23 – -0.18) | -0.20 (-0.22 – -0.17) | -0.20 (-0.22 – -0.17) | -0.20 (-0.22 – -0.17) | 0.07 (0.05 – 0.09) | -0.20 (-0.22 – -0.17) |
| Parity: First-Born |  |  | *Reference* | *Reference* | *Reference* | *Reference* | *Reference* | *Reference* |
| Parity: Second-Born |  |  | -0.13 (-0.14 – -0.11) | -0.13 (-0.14 – -0.11) | -0.13 (-0.14 – -0.11) | -0.13 (-0.14 – -0.11) | -0.03 (-0.04 – -0.02) | -0.13 (-0.14 – -0.11) |
| Parity: Third-Born |  |  | -0.24 (-0.26 – -0.23) | -0.24 (-0.26 – -0.22) | -0.24 (-0.26 – -0.22) | -0.24 (-0.26 – -0.22) | -0.05 (-0.06 – -0.04) | -0.24 (-0.26 – -0.22) |
| Parity: Fourth-Born |  |  | -0.37 (-0.39 – -0.34) | -0.36 (-0.39 – -0.34) | -0.36 (-0.39 – -0.34) | -0.36 (-0.39 – -0.34) | -0.08 (-0.10 – -0.06) | -0.36 (-0.39 – -0.34) |
| Parity: Fifth-Born or later |  |  | -0.52 (-0.55 – -0.48) | -0.51 (-0.55 – -0.47) | -0.51 (-0.55 – -0.48) | -0.51 (-0.55 – -0.48) | -0.13 (-0.15 – -0.10) | -0.51 (-0.55 – -0.47) |
| (Intercept) | 0.08 (0.08 – 0.09) | 0.12 (0.11 – 0.12) | 0.61 (0.59 – 0.63) | 0.65 (0.63 – 0.67) | 0.65 (0.64 – 0.67) | 0.65 (0.63 – 0.67) | 0.15 (0.13 – 0.16) | 0.65 (0.63 – 0.67) |
| Random Effects | | | | | | | | |
| σ2 | 0.62 | 0.61 | 0.55 | 0.54 | 0.54 | 0.54 | 0.35 | 0.54 || τ00 | 0.38 parents | 0.36 parents | 0.38 parents | 0.37 parents | 0.36 parents | 0.36 parents | 0.06 parents | 0.37 parents || ICC | 0.38 | 0.37 | 0.41 | 0.40 | 0.40 | 0.40 | 0.15 | 0.40 || N | 69694 parents | 69694 parents | 69694 parents | 69694 parents | 69694 parents | 69694 parents | 68474 parents | 69694 parents || Observations | 142757 | 142757 | 142757 | 142757 | 142757 | 142757 | 132285 | 142757 |
| Marginal R2 / Conditional R2 | 0.000 / 0.378 | 0.024 / 0.387 | 0.063 / 0.447 | 0.087 / 0.456 | 0.090 / 0.457 | 0.090 / 0.457 | 0.556 / 0.621 | 0.087 / 0.456 |
