# Supplementary material for: The ADHD deficit in school performance across sex and parental education: A prospective sibling‐comparison register study of 344,152 Norwegian adolescents
Source: JCPP Adv. 2022 Feb 12;2(1):e12064. doi: 10.1002/jcv2.12064 (PMC10242882; doi:10.1002/jcv2.12064)
Supplement: Supplementary file 1 — Supplementary Material S1 [file JCV2-2-e12064-s001.zip › Supporting Information/Supplementary Tables/Table S17.html]

Table S17: Regression Table – Reading, 9th grade (Sibling Models)

| Dependent Variable: Test Score (z-score) | Empty Sibling Model | ADHD Only | Covariates Only | Full Sibling Model | + Number of Diagnoses | + Specific Diagnoses | + Early School Performance | Interaction w/ Sex |
| Predictors | Estimates (95% CIs) | Estimates (95% CIs) | Estimates (95% CIs) | Estimates (95% CIs) | Estimates (95% CIs) | Estimates (95% CIs) | Estimates (95% CIs) | Estimates (95% CIs) |
| ADHD (P81) Within Families |  | -0.68 (-0.71 – -0.64) |  | -0.59 (-0.62 – -0.56) | -0.58 (-0.61 – -0.54) | -0.58 (-0.61 – -0.54) | -0.24 (-0.27 – -0.21) | -0.59 (-0.63 – -0.55) |
| ADHD (P81) Between Families |  | -0.41 (-0.47 – -0.36) |  | -0.42 (-0.48 – -0.37) | -0.41 (-0.46 – -0.36) | -0.41 (-0.46 – -0.35) | -0.15 (-0.19 – -0.11) | -0.42 (-0.48 – -0.37) |
| Sex: Boys |  |  | *Reference* | *Reference* | *Reference* | *Reference* | *Reference* | *Reference* |
| Sex: Girls |  |  | 0.27 (0.26 – 0.28) | 0.25 (0.24 – 0.26) | 0.26 (0.25 – 0.27) | 0.26 (0.25 – 0.26) | 0.21 (0.21 – 0.22) | 0.25 (0.24 – 0.26) |
| ADHD \* Girls *(Interaction)* |  |  |  |  |  |  |  | 0.00 (-0.06 – 0.06) |
| Early School Performance: Mathematics (z-score) |  |  |  |  |  |  | 0.22 (0.21 – 0.22) |  |
| Early School Performance: Reading (z-score) |  |  |  |  |  |  | 0.46 (0.45 – 0.46) |  |
| Number of Diagnoses: No other diagnoses |  |  |  |  | *Reference* |  |  |  |
| Number of Diagnoses: One other diagnosis |  |  |  |  | -0.19 (-0.21 – -0.17) |  |  |  |
| Number of Diagnoses: Two other diagnoses |  |  |  |  | -0.22 (-0.29 – -0.16) |  |  |  |
| Number of Diagnoses: Three or more other diagnoses |  |  |  |  | -0.16 (-0.32 – -0.01) |  |  |  |
| Anxiety Disorder (P74) |  |  |  |  |  | -0.16 (-0.21 – -0.11) |  |  |
| Somatization Disorder (P75) |  |  |  |  |  | -0.12 (-0.21 – -0.04) |  |  |
| Depressive Disorder (P76) |  |  |  |  |  | -0.10 (-0.13 – -0.06) |  |  |
| Suicide / Suicide Attempt (P77) |  |  |  |  |  | -0.29 (-0.39 – -0.19) |  |  |
| Phobia / Compulsive Disorder (P79) |  |  |  |  |  | -0.03 (-0.09 – 0.02) |  |  |
| Personality Disorder (P80) |  |  |  |  |  | -0.08 (-0.22 – 0.06) |  |  |
| PTSD (P82) |  |  |  |  |  | -0.23 (-0.35 – -0.11) |  |  |
| Anorexia Nervosa / Bulimia (P86) |  |  |  |  |  | 0.16 (0.06 – 0.27) |  |  |
| Other Psychological Disorders (P99) |  |  |  |  |  | -0.30 (-0.34 – -0.25) |  |  |
| Birth Year: 1997 |  |  | *Reference* | *Reference* | *Reference* | *Reference* | *Reference* | *Reference* |
| Birth Year: 1998 |  |  | 0.21 (0.19 – 0.22) | 0.21 (0.19 – 0.22) | 0.21 (0.19 – 0.22) | 0.21 (0.19 – 0.22) | 0.24 (0.22 – 0.25) | 0.21 (0.19 – 0.22) |
| Birth Year: 1999 |  |  | 0.23 (0.21 – 0.24) | 0.23 (0.21 – 0.24) | 0.23 (0.21 – 0.24) | 0.23 (0.21 – 0.24) | 0.16 (0.15 – 0.18) | 0.23 (0.21 – 0.24) |
| Birth Year: 2000 |  |  | -0.05 (-0.07 – -0.03) | -0.05 (-0.06 – -0.03) | -0.05 (-0.06 – -0.03) | -0.05 (-0.06 – -0.03) | -0.04 (-0.05 – -0.02) | -0.05 (-0.06 – -0.03) |
| Birth Year: 2001 |  |  | -0.07 (-0.09 – -0.06) | -0.07 (-0.09 – -0.06) | -0.07 (-0.09 – -0.05) | -0.07 (-0.09 – -0.06) | -0.26 (-0.28 – -0.25) | -0.07 (-0.09 – -0.06) |
| Birth Year: 2002 |  |  | -0.17 (-0.18 – -0.15) | -0.17 (-0.18 – -0.15) | -0.16 (-0.18 – -0.14) | -0.16 (-0.18 – -0.15) | -0.18 (-0.19 – -0.16) | -0.17 (-0.18 – -0.15) |
| Birth Month: January |  |  | *Reference* | *Reference* | *Reference* | *Reference* | *Reference* | *Reference* |
| Birth Month: February |  |  | -0.02 (-0.05 – -0.00) | -0.02 (-0.05 – -0.00) | -0.02 (-0.05 – -0.00) | -0.02 (-0.05 – -0.00) | -0.01 (-0.02 – 0.01) | -0.02 (-0.05 – -0.00) |
| Birth Month: March |  |  | -0.04 (-0.06 – -0.01) | -0.04 (-0.06 – -0.01) | -0.04 (-0.06 – -0.01) | -0.04 (-0.06 – -0.01) | 0.00 (-0.02 – 0.02) | -0.04 (-0.06 – -0.01) |
| Birth Month: April |  |  | -0.04 (-0.06 – -0.01) | -0.04 (-0.06 – -0.01) | -0.03 (-0.06 – -0.01) | -0.03 (-0.06 – -0.01) | 0.01 (-0.00 – 0.03) | -0.04 (-0.06 – -0.01) |
| Birth Month: May |  |  | -0.07 (-0.09 – -0.04) | -0.06 (-0.09 – -0.04) | -0.06 (-0.09 – -0.04) | -0.06 (-0.09 – -0.04) | 0.02 (0.00 – 0.04) | -0.06 (-0.09 – -0.04) |
| Birth Month: June |  |  | -0.08 (-0.10 – -0.05) | -0.07 (-0.10 – -0.05) | -0.07 (-0.09 – -0.05) | -0.07 (-0.09 – -0.05) | 0.03 (0.01 – 0.05) | -0.07 (-0.10 – -0.05) |
| Birth Month: July |  |  | -0.11 (-0.14 – -0.09) | -0.11 (-0.13 – -0.09) | -0.11 (-0.13 – -0.09) | -0.11 (-0.13 – -0.08) | 0.02 (0.01 – 0.04) | -0.11 (-0.13 – -0.09) |
| Birth Month: August |  |  | -0.13 (-0.15 – -0.11) | -0.12 (-0.15 – -0.10) | -0.12 (-0.14 – -0.10) | -0.12 (-0.14 – -0.10) | 0.03 (0.01 – 0.05) | -0.12 (-0.15 – -0.10) |
| Birth Month: September |  |  | -0.16 (-0.18 – -0.14) | -0.15 (-0.17 – -0.13) | -0.15 (-0.17 – -0.13) | -0.15 (-0.17 – -0.13) | 0.03 (0.02 – 0.05) | -0.15 (-0.17 – -0.13) |
| Birth Month: October |  |  | -0.17 (-0.19 – -0.14) | -0.16 (-0.18 – -0.14) | -0.16 (-0.18 – -0.14) | -0.16 (-0.18 – -0.14) | 0.04 (0.02 – 0.06) | -0.16 (-0.18 – -0.14) |
| Birth Month: November |  |  | -0.21 (-0.23 – -0.18) | -0.20 (-0.22 – -0.17) | -0.19 (-0.22 – -0.17) | -0.19 (-0.22 – -0.17) | 0.05 (0.03 – 0.06) | -0.20 (-0.22 – -0.17) |
| Birth Month: December |  |  | -0.22 (-0.25 – -0.20) | -0.22 (-0.24 – -0.19) | -0.21 (-0.24 – -0.19) | -0.21 (-0.24 – -0.19) | 0.04 (0.03 – 0.06) | -0.22 (-0.24 – -0.19) |
| Parity: First-Born |  |  | *Reference* | *Reference* | *Reference* | *Reference* | *Reference* | *Reference* |
| Parity: Second-Born |  |  | -0.17 (-0.18 – -0.16) | -0.17 (-0.18 – -0.15) | -0.17 (-0.18 – -0.15) | -0.17 (-0.18 – -0.16) | -0.06 (-0.07 – -0.05) | -0.17 (-0.18 – -0.15) |
| Parity: Third-Born |  |  | -0.29 (-0.31 – -0.27) | -0.29 (-0.30 – -0.27) | -0.29 (-0.30 – -0.27) | -0.29 (-0.30 – -0.27) | -0.09 (-0.10 – -0.08) | -0.29 (-0.30 – -0.27) |
| Parity: Fourth-Born |  |  | -0.41 (-0.43 – -0.38) | -0.40 (-0.43 – -0.38) | -0.40 (-0.43 – -0.38) | -0.40 (-0.43 – -0.38) | -0.10 (-0.12 – -0.08) | -0.40 (-0.43 – -0.38) |
| Parity: Fifth-Born or later |  |  | -0.56 (-0.60 – -0.52) | -0.55 (-0.59 – -0.52) | -0.56 (-0.59 – -0.52) | -0.56 (-0.59 – -0.52) | -0.18 (-0.21 – -0.16) | -0.55 (-0.59 – -0.52) |
| (Intercept) | 0.04 (0.04 – 0.05) | 0.08 (0.07 – 0.08) | 0.14 (0.12 – 0.16) | 0.17 (0.15 – 0.19) | 0.18 (0.16 – 0.20) | 0.18 (0.16 – 0.20) | 0.01 (-0.01 – 0.03) | 0.17 (0.15 – 0.19) |
| Random Effects | | | | | | | | |
| σ2 | 0.66 | 0.65 | 0.58 | 0.57 | 0.57 | 0.57 | 0.41 | 0.57 || τ00 | 0.33 parents | 0.31 parents | 0.33 parents | 0.32 parents | 0.32 parents | 0.32 parents | 0.05 parents | 0.32 parents || ICC | 0.33 | 0.32 | 0.36 | 0.36 | 0.36 | 0.36 | 0.12 | 0.36 || N | 69619 parents | 69619 parents | 69619 parents | 69619 parents | 69619 parents | 69619 parents | 68450 parents | 69619 parents || Observations | 142568 | 142568 | 142568 | 142568 | 142568 | 142568 | 132215 | 142568 |
| Marginal R2 / Conditional R2 | 0.000 / 0.329 | 0.028 / 0.342 | 0.074 / 0.412 | 0.099 / 0.422 | 0.100 / 0.423 | 0.100 / 0.423 | 0.480 / 0.540 | 0.099 / 0.422 |
