# Supplementary material for: The ADHD deficit in school performance across sex and parental education: A prospective sibling‐comparison register study of 344,152 Norwegian adolescents
Source: JCPP Adv. 2022 Feb 12;2(1):e12064. doi: 10.1002/jcv2.12064 (PMC10242882; doi:10.1002/jcv2.12064)
Supplement: Supplementary file 1 — Supplementary Material S1 [file JCV2-2-e12064-s001.zip › Supporting Information/Supplementary Tables/Table S18.html]

Table S18: Regression Table – Registered GPA (Bivariate and Adjusted Logistic Regression Models)

| Dependent Variable: Lack of GPA (GPA lacking = 1) | Bivariate: ADHD | Covariates Only | Fully Adjusted | + Number of Diagnoses | + Specific Diagnoses | + Early School Performance | Interaction w/ Sex | Interaction w/ Parental Education |
| Predictors | Odds Ratio (95% CIs) | Odds Ratio (95% CIs) | Odds Ratio (95% CIs) | Odds Ratio (95% CIs) | Odds Ratio (95% CIs) | Odds Ratio (95% CIs) | Odds Ratio (95% CIs) | Odds Ratio (95% CIs) |
| ADHD (P81) | 4.69 (4.48 – 4.91) |  | 4.28 (4.08 – 4.49) | 2.99 (2.84 – 3.15) | 3.23 (3.07 – 3.39) | 3.26 (3.03 – 3.52) | 4.17 (3.94 – 4.41) | 4.06 (3.67 – 4.48) |
| Sex: Boys |  | *Reference* | *Reference* | *Reference* | *Reference* | *Reference* | *Reference* | *Reference* |
| Sex: Girls |  | 0.78 (0.76 – 0.81) | 0.86 (0.83 – 0.89) | 0.73 (0.71 – 0.76) | 0.81 (0.79 – 0.84) | 1.03 (0.98 – 1.08) | 0.85 (0.82 – 0.88) | 0.86 (0.83 – 0.89) |
| Parental Education: No High School |  | *Reference* | *Reference* | *Reference* | *Reference* | *Reference* | *Reference* | *Reference* |
| Parental Education: High School |  | 0.47 (0.45 – 0.50) | 0.50 (0.47 – 0.52) | 0.51 (0.48 – 0.54) | 0.50 (0.47 – 0.53) | 0.59 (0.54 – 0.63) | 0.50 (0.47 – 0.52) | 0.48 (0.45 – 0.51) |
| Parental Education: Bachelor's Degree (or equiv) |  | 0.36 (0.35 – 0.38) | 0.40 (0.38 – 0.43) | 0.43 (0.41 – 0.45) | 0.41 (0.39 – 0.44) | 0.62 (0.57 – 0.67) | 0.40 (0.38 – 0.43) | 0.40 (0.38 – 0.42) |
| Parental Education: Master's Degree (or equiv) |  | 0.43 (0.41 – 0.46) | 0.50 (0.47 – 0.54) | 0.56 (0.53 – 0.60) | 0.53 (0.50 – 0.57) | 0.91 (0.83 – 0.99) | 0.50 (0.47 – 0.54) | 0.50 (0.47 – 0.54) |
| Parental Education: Missing |  | 1.35 (1.27 – 1.45) | 1.49 (1.39 – 1.59) | 1.65 (1.53 – 1.77) | 1.57 (1.47 – 1.69) | 1.53 (1.37 – 1.70) | 1.49 (1.39 – 1.59) | 1.57 (1.46 – 1.69) |
| ADHD \* Girls *(Interaction)* |  |  |  |  |  |  | 1.09 (0.99 – 1.21) |  |
| ADHD \* Parental Ed: High School *(Interaction)* |  |  |  |  |  |  |  | 1.22 (1.08 – 1.38) |
| ADHD \* Parental Ed: Bachelor *(Interaction)* |  |  |  |  |  |  |  | 1.11 (0.97 – 1.28) |
| ADHD \* Parental Ed: Master *(Interaction)* |  |  |  |  |  |  |  | 0.88 (0.72 – 1.09) |
| ADHD \* Parental Ed: Missing *(Interaction)* |  |  |  |  |  |  |  | 0.53 (0.42 – 0.66) |
| Early School Performance: Mathematics (z-score) |  |  |  |  |  | 0.71 (0.69 – 0.73) |  |  |
| Early School Performance: Reading (z-score) |  |  |  |  |  | 0.94 (0.91 – 0.97) |  |  |
| Number of Diagnoses: No other diagnoses |  |  |  | *Reference* |  |  |  |  |
| Number of Diagnoses: One other diagnosis |  |  |  | 7.10 (6.81 – 7.40) |  |  |  |  |
| Number of Diagnoses: Two other diagnoses |  |  |  | 15.34 (14.22 – 16.54) |  |  |  |  |
| Number of Diagnoses: Three or more other diagnoses |  |  |  | 23.31 (19.92 – 27.27) |  |  |  |  |
| Anxiety Disorder (P74) |  |  |  |  | 2.26 (2.04 – 2.49) |  |  |  |
| Somatization Disorder (P75) |  |  |  |  | 1.69 (1.37 – 2.07) |  |  |  |
| Depressive Disorder (P76 |  |  |  |  | 2.48 (2.31 – 2.66) |  |  |  |
| Suicide / Suicide Attempt (P77) |  |  |  |  | 2.53 (2.18 – 2.93) |  |  |  |
| Phobia / Compulsive Disorder (P79) |  |  |  |  | 2.12 (1.89 – 2.37) |  |  |  |
| Personality Disorder (P80) |  |  |  |  | 2.03 (1.57 – 2.61) |  |  |  |
| PTSD (P82) |  |  |  |  | 2.18 (1.74 – 2.72) |  |  |  |
| Anorexia Nervosa / Bulimia (P86) |  |  |  |  | 2.56 (2.01 – 3.26) |  |  |  |
| Other Psychological Disorders (P99) |  |  |  |  | 9.70 (9.15 – 10.28) |  |  |  |
| Birth Year: 1997 |  | *Reference* | *Reference* | *Reference* | *Reference* | *Reference* | *Reference* | *Reference* |
| Birth Year: 1998 |  | 1.03 (0.97 – 1.09) | 1.03 (0.97 – 1.09) | 1.01 (0.95 – 1.07) | 1.01 (0.95 – 1.07) | 0.94 (0.86 – 1.03) | 1.03 (0.97 – 1.09) | 1.03 (0.97 – 1.09) |
| Birth Year: 1999 |  | 1.08 (1.02 – 1.14) | 1.07 (1.01 – 1.13) | 1.03 (0.97 – 1.10) | 1.04 (0.98 – 1.10) | 0.97 (0.88 – 1.06) | 1.07 (1.01 – 1.13) | 1.07 (1.01 – 1.13) |
| Birth Year: 2000 |  | 1.09 (1.03 – 1.16) | 1.09 (1.02 – 1.15) | 1.06 (1.00 – 1.13) | 1.07 (1.01 – 1.13) | 1.04 (0.95 – 1.13) | 1.08 (1.02 – 1.15) | 1.08 (1.02 – 1.15) |
| Birth Year: 2001 |  | 1.17 (1.10 – 1.24) | 1.15 (1.09 – 1.22) | 1.11 (1.05 – 1.18) | 1.12 (1.06 – 1.19) | 1.06 (0.97 – 1.17) | 1.15 (1.09 – 1.22) | 1.15 (1.09 – 1.22) |
| Birth Year: 2002 |  | 1.43 (1.35 – 1.51) | 1.42 (1.35 – 1.51) | 1.39 (1.31 – 1.47) | 1.41 (1.33 – 1.49) | 1.70 (1.57 – 1.84) | 1.42 (1.35 – 1.50) | 1.42 (1.34 – 1.50) |
| Birth Month: January |  | *Reference* | *Reference* | *Reference* | *Reference* | *Reference* | *Reference* | *Reference* |
| Birth Month: February |  | 1.14 (1.05 – 1.24) | 1.14 (1.05 – 1.24) | 1.15 (1.06 – 1.25) | 1.15 (1.06 – 1.25) | 1.05 (0.93 – 1.18) | 1.14 (1.05 – 1.24) | 1.14 (1.05 – 1.24) |
| Birth Month: March |  | 0.99 (0.92 – 1.08) | 0.99 (0.91 – 1.07) | 0.99 (0.91 – 1.07) | 0.98 (0.90 – 1.07) | 1.05 (0.93 – 1.17) | 0.99 (0.91 – 1.07) | 0.99 (0.91 – 1.07) |
| Birth Month: April |  | 1.04 (0.96 – 1.12) | 1.03 (0.95 – 1.12) | 1.02 (0.94 – 1.11) | 1.02 (0.94 – 1.11) | 1.07 (0.96 – 1.20) | 1.03 (0.95 – 1.12) | 1.03 (0.95 – 1.12) |
| Birth Month: May |  | 1.05 (0.97 – 1.14) | 1.04 (0.96 – 1.13) | 1.03 (0.95 – 1.12) | 1.04 (0.95 – 1.13) | 1.00 (0.89 – 1.12) | 1.04 (0.96 – 1.12) | 1.04 (0.96 – 1.12) |
| Birth Month: June |  | 1.03 (0.95 – 1.12) | 1.02 (0.94 – 1.11) | 1.00 (0.92 – 1.09) | 1.01 (0.93 – 1.10) | 0.95 (0.84 – 1.07) | 1.02 (0.94 – 1.11) | 1.02 (0.94 – 1.11) |
| Birth Month: July |  | 1.09 (1.00 – 1.18) | 1.06 (0.98 – 1.15) | 1.06 (0.97 – 1.15) | 1.06 (0.98 – 1.16) | 0.93 (0.82 – 1.04) | 1.06 (0.98 – 1.15) | 1.06 (0.98 – 1.15) |
| Birth Month: August |  | 1.08 (1.00 – 1.17) | 1.05 (0.97 – 1.14) | 1.03 (0.95 – 1.12) | 1.04 (0.96 – 1.13) | 0.95 (0.85 – 1.07) | 1.05 (0.97 – 1.14) | 1.05 (0.97 – 1.14) |
| Birth Month: September |  | 1.10 (1.02 – 1.19) | 1.07 (0.99 – 1.16) | 1.07 (0.98 – 1.16) | 1.07 (0.98 – 1.16) | 0.97 (0.86 – 1.09) | 1.07 (0.99 – 1.16) | 1.07 (0.99 – 1.16) |
| Birth Month: October |  | 1.13 (1.04 – 1.23) | 1.09 (1.01 – 1.19) | 1.08 (0.99 – 1.17) | 1.08 (0.99 – 1.17) | 1.00 (0.89 – 1.12) | 1.09 (1.01 – 1.19) | 1.09 (1.01 – 1.19) |
| Birth Month: November |  | 1.16 (1.07 – 1.26) | 1.10 (1.01 – 1.20) | 1.08 (0.99 – 1.17) | 1.09 (1.00 – 1.19) | 0.96 (0.85 – 1.08) | 1.10 (1.01 – 1.20) | 1.10 (1.01 – 1.20) |
| Birth Month: December |  | 1.36 (1.26 – 1.47) | 1.30 (1.20 – 1.41) | 1.28 (1.18 – 1.39) | 1.29 (1.19 – 1.40) | 1.24 (1.11 – 1.39) | 1.30 (1.20 – 1.41) | 1.30 (1.20 – 1.41) |
| Parity: First-Born |  | *Reference* | *Reference* | *Reference* | *Reference* | *Reference* | *Reference* | *Reference* |
| Parity: Second-Born |  | 0.99 (0.95 – 1.03) | 1.00 (0.96 – 1.04) | 1.04 (1.00 – 1.08) | 1.04 (0.99 – 1.08) | 0.94 (0.89 – 0.99) | 1.00 (0.96 – 1.04) | 1.00 (0.96 – 1.04) |
| Parity: Third-Born |  | 1.03 (0.99 – 1.08) | 1.06 (1.01 – 1.11) | 1.11 (1.05 – 1.16) | 1.10 (1.05 – 1.15) | 1.00 (0.94 – 1.07) | 1.06 (1.01 – 1.11) | 1.06 (1.01 – 1.11) |
| Parity: Fourth-Born |  | 1.22 (1.14 – 1.32) | 1.26 (1.17 – 1.36) | 1.33 (1.24 – 1.44) | 1.32 (1.22 – 1.42) | 1.08 (0.97 – 1.20) | 1.26 (1.17 – 1.36) | 1.26 (1.17 – 1.36) |
| Parity: Fifth-Born or later |  | 1.37 (1.25 – 1.51) | 1.45 (1.31 – 1.59) | 1.61 (1.46 – 1.78) | 1.54 (1.39 – 1.70) | 1.22 (1.05 – 1.41) | 1.45 (1.31 – 1.59) | 1.44 (1.31 – 1.59) |
| Parity: Missing |  | 2.21 (1.72 – 2.85) | 2.38 (1.85 – 3.07) | 2.80 (2.15 – 3.65) | 2.65 (2.05 – 3.43) | 2.10 (1.35 – 3.26) | 2.38 (1.84 – 3.07) | 2.29 (1.78 – 2.95) |
| (Intercept) | 0.04 (0.04 – 0.04) | 0.08 (0.07 – 0.08) | 0.06 (0.06 – 0.07) | 0.05 (0.04 – 0.05) | 0.05 (0.05 – 0.06) | 0.02 (0.02 – 0.03) | 0.06 (0.06 – 0.07) | 0.06 (0.06 – 0.07) |
| Observations | 359492 | 359492 | 359492 | 359492 | 359492 | 322605 | 359492 | 359492 |
| R2 Tjur | 0.014 | 0.012 | 0.025 | 0.086 | 0.069 | 0.015 | 0.025 | 0.025 |
