# Supplementary material for: The ADHD deficit in school performance across sex and parental education: A prospective sibling‐comparison register study of 344,152 Norwegian adolescents
Source: JCPP Adv. 2022 Feb 12;2(1):e12064. doi: 10.1002/jcv2.12064 (PMC10242882; doi:10.1002/jcv2.12064)
Supplement: Supplementary file 1 — Supplementary Material S1 [file JCV2-2-e12064-s001.zip › Supporting Information/Supplementary Tables/Table S2.docx]

# Table S2: Prevalence of Different Disorders

| **Table S2** | | | |
| --- | --- | --- | --- |
| Prevalence of Different Disorders | | | |
| **ICPC-2 Code** | **Name** | **Prevalence*** | **Included**** |
| P70 | Dementia | 0.00 % | No |
| P71 | Organic Psychosis Disorder | 0.02 % | No |
| P72 | Schizophrenia | 0.01 % | No |
| P73 | Affective Psychosis | 0.05 % | No |
| **P74** | **Anxiety Disorder / Anxiety State** | **1.09 %** | **Yes** |
| **P75** | **Somatization Disorder** | **0.30 %** | **Yes** |
| **P76** | **Depressive Disorder** | **2.43 %** | **Yes** |
| **P77** | **Suicide / Suicide Attempt** | **0.30 %** | **Yes** |
| P78 | Neuraesthenia / Surmenage | 0.07 % | No |
| **P79** | **Phobia / Compulsive Disorder** | **0.85 %** | **Yes** |
| **P80** | **Personality Disorder** | **0.13 %** | **Yes** |
| **P81** | **Hyperkinetic Disorder** | **4.01 %** | **Yes** |
| **P82** | **Post-Traumatic Stress Disorder** | **0.18 %** | **Yes** |
| P85 | Mental Retardation | 0.06 % | No |
| **P86** | **Anorexia Nervosa / Bulimia** | **0.19 %** | **Yes** |
| P98 | Psychosis NOS / Other | 0.05 % | No |
| **P99** | **Psychological Disorders, Other** | **1.26 %** | **Yes** |
| * *At least one registration between age 10 to 16* | | | |
| ** *Disorders with a prevalence > 0.1% (marked in bold) are included as separate indicator variables in the analyses that adjusts for individual comorbid disorders. All diagnoses were included in the analyses that adjusts for number of comorbid disorders.* | | | |
