# Supplementary material for: The ADHD deficit in school performance across sex and parental education: A prospective sibling‐comparison register study of 344,152 Norwegian adolescents
Source: JCPP Adv. 2022 Feb 12;2(1):e12064. doi: 10.1002/jcv2.12064 (PMC10242882; doi:10.1002/jcv2.12064)
Supplement: Supplementary file 1 — Supplementary Material S1 [file JCV2-2-e12064-s001.zip › Supporting Information/Supplementary Tables/Table S3.html]

Table S3: Regression Table – GPA (Bivariate and Adjusted Models)

| Dependent Variable: GPA (z-score) | Bivariate: ADHD | Covariates Only | Fully Adjusted | + Number of Diagnoses | + Specific Diagnoses | + Early School Performance | Interaction w/ Sex | Interaction w/ Parental Education |
| Predictors | Estimates (95% CIs) | Estimates (95% CIs) | Estimates (95% CIs) | Estimates (95% CIs) | Estimates (95% CIs) | Estimates (95% CIs) | Estimates (95% CIs) | Estimates (95% CIs) |
| ADHD (P81) | -1.11 (-1.12 – -1.09) |  | -0.86 (-0.88 – -0.85) | -0.82 (-0.83 – -0.80) | -0.82 (-0.83 – -0.80) | -0.54 (-0.56 – -0.53) | -0.81 (-0.83 – -0.79) | -0.70 (-0.74 – -0.67) |
| Sex: Boys |  | *Reference* | *Reference* | *Reference* | *Reference* | *Reference* | *Reference* | *Reference* |
| Sex: Girls |  | 0.52 (0.52 – 0.53) | 0.50 (0.49 – 0.50) | 0.51 (0.50 – 0.52) | 0.51 (0.50 – 0.51) | 0.50 (0.49 – 0.50) | 0.50 (0.50 – 0.51) | 0.50 (0.49 – 0.50) |
| Parental Education: No High School |  | *Reference* | *Reference* | *Reference* | *Reference* | *Reference* | *Reference* | *Reference* |
| Parental Education: High School |  | 0.36 (0.35 – 0.38) | 0.35 (0.34 – 0.36) | 0.34 (0.33 – 0.36) | 0.34 (0.33 – 0.35) | 0.18 (0.17 – 0.19) | 0.35 (0.34 – 0.36) | 0.36 (0.35 – 0.37) |
| Parental Education: Bachelor's Degree (or equiv) |  | 0.86 (0.85 – 0.87) | 0.82 (0.81 – 0.83) | 0.82 (0.81 – 0.83) | 0.82 (0.81 – 0.83) | 0.45 (0.44 – 0.46) | 0.82 (0.81 – 0.83) | 0.84 (0.83 – 0.85) |
| Parental Education: Master's Degree (or equiv) |  | 1.26 (1.25 – 1.27) | 1.21 (1.20 – 1.23) | 1.20 (1.19 – 1.22) | 1.20 (1.19 – 1.22) | 0.65 (0.64 – 0.66) | 1.21 (1.20 – 1.23) | 1.23 (1.22 – 1.24) |
| Parental Education: Missing |  | 0.20 (0.19 – 0.22) | 0.18 (0.16 – 0.20) | 0.18 (0.16 – 0.19) | 0.18 (0.16 – 0.19) | 0.20 (0.18 – 0.21) | 0.18 (0.16 – 0.20) | 0.18 (0.17 – 0.20) |
| ADHD \* Girls *(Interaction)* |  |  |  |  |  |  | -0.17 (-0.20 – -0.14) |  |
| ADHD \* Parental Ed: High School *(Interaction)* |  |  |  |  |  |  |  | -0.15 (-0.19 – -0.11) |
| ADHD \* Parental Ed: Bachelor *(Interaction)* |  |  |  |  |  |  |  | -0.25 (-0.29 – -0.20) |
| ADHD \* Parental Ed: Master *(Interaction)* |  |  |  |  |  |  |  | -0.26 (-0.33 – -0.20) |
| ADHD \* Parental Ed: Missing *(Interaction)* |  |  |  |  |  |  |  | -0.02 (-0.11 – 0.06) |
| Early School Performance: Mathematics (z-score) |  |  |  |  |  | 0.30 (0.30 – 0.31) |  |  |
| Early School Performance: Reading (z-score) |  |  |  |  |  | 0.28 (0.27 – 0.28) |  |  |
| Number of Diagnoses: No other diagnoses |  |  |  | *Reference* |  |  |  |  |
| Number of Diagnoses: One other diagnosis |  |  |  | -0.37 (-0.39 – -0.36) |  |  |  |  |
| Number of Diagnoses: Two other diagnoses |  |  |  | -0.57 (-0.60 – -0.54) |  |  |  |  |
| Number of Diagnoses: Three or more other diagnoses |  |  |  | -0.66 (-0.75 – -0.58) |  |  |  |  |
| Anxiety Disorder (P74) |  |  |  |  | -0.25 (-0.27 – -0.22) |  |  |  |
| Somatization Disorder (P75) |  |  |  |  | -0.15 (-0.20 – -0.10) |  |  |  |
| Depressive Disorder (P76 |  |  |  |  | -0.39 (-0.41 – -0.37) |  |  |  |
| Suicide / Suicide Attempt (P77) |  |  |  |  | -0.51 (-0.57 – -0.46) |  |  |  |
| Phobia / Compulsive Disorder (P79) |  |  |  |  | -0.12 (-0.15 – -0.09) |  |  |  |
| Personality Disorder (P80) |  |  |  |  | -0.43 (-0.51 – -0.35) |  |  |  |
| PTSD (P82) |  |  |  |  | -0.47 (-0.53 – -0.40) |  |  |  |
| Anorexia Nervosa / Bulimia (P86) |  |  |  |  | 0.14 (0.07 – 0.20) |  |  |  |
| Other Psychological Disorders (P99) |  |  |  |  | -0.42 (-0.45 – -0.39) |  |  |  |
| Birth Year: 1997 |  | *Reference* | *Reference* | *Reference* | *Reference* | *Reference* | *Reference* | *Reference* |
| Birth Year: 1998 |  | 0.03 (0.02 – 0.04) | 0.03 (0.02 – 0.04) | 0.03 (0.02 – 0.04) | 0.03 (0.02 – 0.04) | 0.14 (0.13 – 0.15) | 0.03 (0.02 – 0.04) | 0.03 (0.02 – 0.04) |
| Birth Year: 1999 |  | 0.06 (0.05 – 0.07) | 0.06 (0.05 – 0.07) | 0.06 (0.05 – 0.07) | 0.06 (0.05 – 0.07) | 0.13 (0.12 – 0.13) | 0.06 (0.05 – 0.07) | 0.06 (0.05 – 0.07) |
| Birth Year: 2000 |  | 0.09 (0.08 – 0.10) | 0.10 (0.09 – 0.11) | 0.10 (0.09 – 0.11) | 0.10 (0.09 – 0.11) | 0.22 (0.21 – 0.23) | 0.10 (0.09 – 0.11) | 0.10 (0.09 – 0.11) |
| Birth Year: 2001 |  | 0.11 (0.10 – 0.12) | 0.11 (0.10 – 0.12) | 0.12 (0.11 – 0.13) | 0.12 (0.11 – 0.13) | 0.13 (0.12 – 0.14) | 0.11 (0.10 – 0.12) | 0.12 (0.11 – 0.12) |
| Birth Year: 2002 |  | 0.15 (0.14 – 0.16) | 0.16 (0.15 – 0.17) | 0.16 (0.15 – 0.17) | 0.16 (0.15 – 0.17) | 0.27 (0.26 – 0.28) | 0.16 (0.15 – 0.17) | 0.16 (0.15 – 0.17) |
| Birth Month: January |  | *Reference* | *Reference* | *Reference* | *Reference* | *Reference* | *Reference* | *Reference* |
| Birth Month: February |  | -0.02 (-0.04 – -0.01) | -0.02 (-0.04 – -0.01) | -0.02 (-0.04 – -0.01) | -0.02 (-0.04 – -0.01) | -0.00 (-0.02 – 0.01) | -0.02 (-0.04 – -0.01) | -0.02 (-0.04 – -0.01) |
| Birth Month: March |  | -0.04 (-0.06 – -0.03) | -0.04 (-0.06 – -0.03) | -0.04 (-0.06 – -0.03) | -0.04 (-0.06 – -0.03) | -0.01 (-0.02 – 0.00) | -0.04 (-0.06 – -0.03) | -0.04 (-0.06 – -0.03) |
| Birth Month: April |  | -0.04 (-0.06 – -0.03) | -0.04 (-0.05 – -0.03) | -0.04 (-0.05 – -0.02) | -0.04 (-0.05 – -0.02) | 0.01 (-0.01 – 0.02) | -0.04 (-0.05 – -0.03) | -0.04 (-0.05 – -0.03) |
| Birth Month: May |  | -0.07 (-0.09 – -0.06) | -0.07 (-0.08 – -0.06) | -0.07 (-0.08 – -0.06) | -0.07 (-0.08 – -0.06) | 0.00 (-0.01 – 0.02) | -0.07 (-0.08 – -0.06) | -0.07 (-0.08 – -0.06) |
| Birth Month: June |  | -0.08 (-0.10 – -0.07) | -0.08 (-0.09 – -0.07) | -0.08 (-0.09 – -0.06) | -0.08 (-0.09 – -0.06) | 0.02 (0.00 – 0.03) | -0.08 (-0.09 – -0.07) | -0.08 (-0.09 – -0.07) |
| Birth Month: July |  | -0.12 (-0.13 – -0.10) | -0.11 (-0.12 – -0.10) | -0.11 (-0.12 – -0.10) | -0.11 (-0.12 – -0.10) | 0.01 (-0.01 – 0.02) | -0.11 (-0.12 – -0.10) | -0.11 (-0.12 – -0.10) |
| Birth Month: August |  | -0.13 (-0.14 – -0.12) | -0.12 (-0.14 – -0.11) | -0.12 (-0.13 – -0.11) | -0.12 (-0.13 – -0.11) | 0.01 (-0.00 – 0.02) | -0.12 (-0.14 – -0.11) | -0.12 (-0.14 – -0.11) |
| Birth Month: September |  | -0.15 (-0.17 – -0.14) | -0.14 (-0.16 – -0.13) | -0.14 (-0.16 – -0.13) | -0.14 (-0.16 – -0.13) | 0.01 (0.00 – 0.02) | -0.14 (-0.16 – -0.13) | -0.14 (-0.16 – -0.13) |
| Birth Month: October |  | -0.17 (-0.19 – -0.16) | -0.16 (-0.17 – -0.15) | -0.16 (-0.17 – -0.14) | -0.16 (-0.17 – -0.14) | 0.02 (0.00 – 0.03) | -0.16 (-0.17 – -0.15) | -0.16 (-0.17 – -0.15) |
| Birth Month: November |  | -0.20 (-0.21 – -0.18) | -0.18 (-0.20 – -0.17) | -0.18 (-0.19 – -0.16) | -0.18 (-0.19 – -0.16) | 0.02 (0.01 – 0.04) | -0.18 (-0.20 – -0.17) | -0.18 (-0.20 – -0.17) |
| Birth Month: December |  | -0.21 (-0.23 – -0.20) | -0.20 (-0.21 – -0.19) | -0.20 (-0.21 – -0.18) | -0.20 (-0.21 – -0.18) | 0.02 (0.00 – 0.03) | -0.20 (-0.21 – -0.19) | -0.20 (-0.22 – -0.19) |
| Parity: First-Born |  | *Reference* | *Reference* | *Reference* | *Reference* | *Reference* | *Reference* | *Reference* |
| Parity: Second-Born |  | -0.07 (-0.08 – -0.07) | -0.08 (-0.08 – -0.07) | -0.08 (-0.08 – -0.07) | -0.08 (-0.08 – -0.07) | -0.02 (-0.03 – -0.02) | -0.08 (-0.08 – -0.07) | -0.08 (-0.08 – -0.07) |
| Parity: Third-Born |  | -0.11 (-0.12 – -0.10) | -0.12 (-0.13 – -0.11) | -0.12 (-0.13 – -0.12) | -0.12 (-0.13 – -0.11) | -0.03 (-0.04 – -0.03) | -0.12 (-0.13 – -0.11) | -0.12 (-0.13 – -0.11) |
| Parity: Fourth-Born |  | -0.18 (-0.20 – -0.17) | -0.19 (-0.20 – -0.18) | -0.19 (-0.21 – -0.18) | -0.19 (-0.21 – -0.18) | -0.06 (-0.07 – -0.05) | -0.19 (-0.20 – -0.18) | -0.19 (-0.20 – -0.18) |
| Parity: Fifth-Born or later |  | -0.24 (-0.26 – -0.22) | -0.25 (-0.27 – -0.23) | -0.26 (-0.28 – -0.24) | -0.26 (-0.28 – -0.24) | -0.07 (-0.09 – -0.05) | -0.25 (-0.27 – -0.23) | -0.25 (-0.27 – -0.23) |
| Parity: Missing |  | -0.13 (-0.23 – -0.03) | -0.14 (-0.24 – -0.04) | -0.15 (-0.25 – -0.05) | -0.15 (-0.24 – -0.05) | 0.03 (-0.07 – 0.13) | -0.14 (-0.24 – -0.04) | -0.14 (-0.23 – -0.04) |
| (Intercept) | 0.04 (0.04 – 0.05) | -0.82 (-0.84 – -0.80) | -0.75 (-0.77 – -0.74) | -0.73 (-0.75 – -0.72) | -0.73 (-0.75 – -0.72) | -0.66 (-0.68 – -0.65) | -0.76 (-0.77 – -0.74) | -0.76 (-0.78 – -0.75) |
| Observations | 344152 | 344152 | 344152 | 344152 | 344152 | 315387 | 344152 | 344152 |
| R2 / R2 adjusted | 0.047 / 0.047 | 0.231 / 0.231 | 0.260 / 0.260 | 0.269 / 0.269 | 0.270 / 0.269 | 0.480 / 0.480 | 0.260 / 0.260 | 0.260 / 0.260 |
