# Supplementary material for: The ADHD deficit in school performance across sex and parental education: A prospective sibling‐comparison register study of 344,152 Norwegian adolescents
Source: JCPP Adv. 2022 Feb 12;2(1):e12064. doi: 10.1002/jcv2.12064 (PMC10242882; doi:10.1002/jcv2.12064)
Supplement: Supplementary file 1 — Supplementary Material S1 [file JCV2-2-e12064-s001.zip › Supporting Information/Supplementary Tables/Table S4.html]

Table S4: Regression Table – GPA (Sibling Models)

| Dependent Variable: GPA (z-score) | Empty Sibling Model | ADHD Only | Covariates Only | Full Sibling Model | + Number of Diagnoses | + Specific Diagnoses | + Early School Performance | Interaction w/ Sex |
| Predictors | Estimates (95% CIs) | Estimates (95% CIs) | Estimates (95% CIs) | Estimates (95% CIs) | Estimates (95% CIs) | Estimates (95% CIs) | Estimates (95% CIs) | Estimates (95% CIs) |
| ADHD (P81) Within Families |  | -0.74 (-0.77 – -0.71) |  | -0.60 (-0.63 – -0.58) | -0.58 (-0.61 – -0.55) | -0.58 (-0.61 – -0.55) | -0.33 (-0.36 – -0.30) | -0.56 (-0.59 – -0.53) |
| ADHD (P81) Between Families |  | -0.65 (-0.70 – -0.59) |  | -0.66 (-0.71 – -0.61) | -0.63 (-0.68 – -0.58) | -0.63 (-0.68 – -0.58) | -0.43 (-0.48 – -0.39) | -0.66 (-0.71 – -0.61) |
| Sex: Boys |  |  | *Reference* | *Reference* | *Reference* | *Reference* | *Reference* | *Reference* |
| Sex: Girls |  |  | 0.51 (0.51 – 0.52) | 0.49 (0.49 – 0.50) | 0.50 (0.49 – 0.51) | 0.50 (0.49 – 0.51) | 0.50 (0.50 – 0.51) | 0.50 (0.49 – 0.51) |
| ADHD \* Girls *(Interaction)* |  |  |  |  |  |  |  | -0.16 (-0.21 – -0.11) |
| Early School Performance: Mathematics (z-score) |  |  |  |  |  |  | 0.31 (0.30 – 0.31) |  |
| Early School Performance: Reading (z-score) |  |  |  |  |  |  | 0.29 (0.28 – 0.29) |  |
| Number of Diagnoses: No other diagnoses |  |  |  |  | *Reference* |  |  |  |
| Number of Diagnoses: One other diagnosis |  |  |  |  | -0.35 (-0.37 – -0.33) |  |  |  |
| Number of Diagnoses: Two other diagnoses |  |  |  |  | -0.50 (-0.56 – -0.45) |  |  |  |
| Number of Diagnoses: Three or more other diagnoses |  |  |  |  | -0.62 (-0.76 – -0.49) |  |  |  |
| Anxiety Disorder (P74) |  |  |  |  |  | -0.20 (-0.24 – -0.16) |  |  |
| Somatization Disorder (P75) |  |  |  |  |  | -0.16 (-0.23 – -0.08) |  |  |
| Depressive Disorder (P76) |  |  |  |  |  | -0.37 (-0.40 – -0.34) |  |  |
| Suicide / Suicide Attempt (P77) |  |  |  |  |  | -0.45 (-0.54 – -0.36) |  |  |
| Phobia / Compulsive Disorder (P79) |  |  |  |  |  | -0.13 (-0.18 – -0.09) |  |  |
| Personality Disorder (P80) |  |  |  |  |  | -0.35 (-0.47 – -0.23) |  |  |
| PTSD (P82) |  |  |  |  |  | -0.46 (-0.56 – -0.35) |  |  |
| Anorexia Nervosa / Bulimia (P86) |  |  |  |  |  | 0.14 (0.04 – 0.23) |  |  |
| Other Psychological Disorders (P99) |  |  |  |  |  | -0.40 (-0.44 – -0.36) |  |  |
| Birth Year: 1997 |  |  | *Reference* | *Reference* | *Reference* | *Reference* | *Reference* | *Reference* |
| Birth Year: 1998 |  |  | 0.05 (0.04 – 0.07) | 0.05 (0.04 – 0.06) | 0.05 (0.04 – 0.06) | 0.05 (0.04 – 0.06) | 0.16 (0.15 – 0.17) | 0.05 (0.04 – 0.06) |
| Birth Year: 1999 |  |  | 0.09 (0.08 – 0.11) | 0.09 (0.08 – 0.11) | 0.10 (0.08 – 0.11) | 0.09 (0.08 – 0.11) | 0.14 (0.12 – 0.15) | 0.09 (0.08 – 0.11) |
| Birth Year: 2000 |  |  | 0.16 (0.15 – 0.18) | 0.16 (0.15 – 0.18) | 0.16 (0.15 – 0.18) | 0.16 (0.15 – 0.18) | 0.25 (0.24 – 0.27) | 0.16 (0.15 – 0.18) |
| Birth Year: 2001 |  |  | 0.20 (0.19 – 0.22) | 0.20 (0.19 – 0.22) | 0.20 (0.19 – 0.22) | 0.20 (0.19 – 0.22) | 0.16 (0.15 – 0.18) | 0.20 (0.19 – 0.22) |
| Birth Year: 2002 |  |  | 0.25 (0.24 – 0.27) | 0.25 (0.24 – 0.27) | 0.26 (0.24 – 0.27) | 0.26 (0.24 – 0.27) | 0.31 (0.29 – 0.32) | 0.25 (0.24 – 0.27) |
| Birth Month: January |  |  | *Reference* | *Reference* | *Reference* | *Reference* | *Reference* | *Reference* |
| Birth Month: February |  |  | -0.03 (-0.05 – -0.01) | -0.03 (-0.05 – -0.01) | -0.03 (-0.05 – -0.01) | -0.03 (-0.05 – -0.01) | -0.01 (-0.03 – 0.01) | -0.03 (-0.05 – -0.01) |
| Birth Month: March |  |  | -0.04 (-0.06 – -0.02) | -0.04 (-0.06 – -0.02) | -0.04 (-0.06 – -0.02) | -0.04 (-0.06 – -0.02) | -0.01 (-0.02 – 0.01) | -0.04 (-0.06 – -0.02) |
| Birth Month: April |  |  | -0.03 (-0.05 – -0.01) | -0.03 (-0.05 – -0.01) | -0.03 (-0.05 – -0.01) | -0.03 (-0.05 – -0.01) | 0.02 (-0.00 – 0.03) | -0.03 (-0.05 – -0.01) |
| Birth Month: May |  |  | -0.07 (-0.09 – -0.05) | -0.07 (-0.09 – -0.05) | -0.07 (-0.09 – -0.05) | -0.07 (-0.09 – -0.05) | 0.01 (-0.01 – 0.02) | -0.07 (-0.09 – -0.05) |
| Birth Month: June |  |  | -0.08 (-0.10 – -0.06) | -0.08 (-0.10 – -0.06) | -0.07 (-0.09 – -0.05) | -0.07 (-0.09 – -0.05) | 0.01 (-0.01 – 0.03) | -0.08 (-0.10 – -0.06) |
| Birth Month: July |  |  | -0.11 (-0.13 – -0.09) | -0.10 (-0.12 – -0.08) | -0.10 (-0.12 – -0.08) | -0.10 (-0.12 – -0.08) | 0.02 (-0.00 – 0.03) | -0.10 (-0.12 – -0.08) |
| Birth Month: August |  |  | -0.11 (-0.13 – -0.09) | -0.10 (-0.12 – -0.08) | -0.10 (-0.12 – -0.08) | -0.10 (-0.12 – -0.08) | 0.03 (0.01 – 0.04) | -0.10 (-0.12 – -0.08) |
| Birth Month: September |  |  | -0.14 (-0.16 – -0.12) | -0.13 (-0.15 – -0.11) | -0.13 (-0.15 – -0.11) | -0.13 (-0.15 – -0.11) | 0.03 (0.01 – 0.05) | -0.13 (-0.15 – -0.11) |
| Birth Month: October |  |  | -0.15 (-0.17 – -0.13) | -0.15 (-0.17 – -0.13) | -0.14 (-0.16 – -0.12) | -0.14 (-0.16 – -0.12) | 0.03 (0.01 – 0.05) | -0.15 (-0.17 – -0.13) |
| Birth Month: November |  |  | -0.18 (-0.20 – -0.16) | -0.17 (-0.19 – -0.15) | -0.17 (-0.19 – -0.15) | -0.17 (-0.19 – -0.15) | 0.03 (0.01 – 0.05) | -0.17 (-0.19 – -0.15) |
| Birth Month: December |  |  | -0.19 (-0.21 – -0.17) | -0.18 (-0.20 – -0.16) | -0.18 (-0.20 – -0.15) | -0.18 (-0.20 – -0.15) | 0.04 (0.02 – 0.06) | -0.18 (-0.20 – -0.16) |
| Parity: First-Born |  |  | *Reference* | *Reference* | *Reference* | *Reference* | *Reference* | *Reference* |
| Parity: Second-Born |  |  | -0.17 (-0.18 – -0.16) | -0.17 (-0.18 – -0.15) | -0.17 (-0.18 – -0.16) | -0.17 (-0.18 – -0.16) | -0.08 (-0.09 – -0.07) | -0.17 (-0.18 – -0.15) |
| Parity: Third-Born |  |  | -0.29 (-0.31 – -0.28) | -0.29 (-0.31 – -0.27) | -0.29 (-0.31 – -0.28) | -0.29 (-0.31 – -0.28) | -0.13 (-0.15 – -0.12) | -0.29 (-0.31 – -0.27) |
| Parity: Fourth-Born |  |  | -0.42 (-0.44 – -0.39) | -0.41 (-0.43 – -0.39) | -0.41 (-0.44 – -0.39) | -0.41 (-0.44 – -0.39) | -0.18 (-0.20 – -0.16) | -0.41 (-0.43 – -0.39) |
| Parity: Fifth-Born or later |  |  | -0.56 (-0.59 – -0.52) | -0.55 (-0.59 – -0.52) | -0.56 (-0.59 – -0.52) | -0.56 (-0.59 – -0.52) | -0.26 (-0.29 – -0.23) | -0.55 (-0.59 – -0.52) |
| (Intercept) | 0.09 (0.08 – 0.09) | 0.13 (0.13 – 0.14) | -0.04 (-0.06 – -0.03) | 0.00 (-0.02 – 0.02) | 0.01 (-0.00 – 0.03) | 0.01 (-0.00 – 0.03) | -0.24 (-0.26 – -0.23) | -0.00 (-0.02 – 0.02) |
| Random Effects | | | | | | | | |
| σ2 | 0.49 | 0.47 | 0.42 | 0.41 | 0.41 | 0.41 | 0.32 | 0.41 || τ00 | 0.47 parents | 0.45 parents | 0.45 parents | 0.43 parents | 0.42 parents | 0.42 parents | 0.19 parents | 0.43 parents || ICC | 0.49 | 0.49 | 0.52 | 0.51 | 0.51 | 0.51 | 0.38 | 0.51 || N | 69765 parents | 69765 parents | 69765 parents | 69765 parents | 69765 parents | 69765 parents | 68730 parents | 69765 parents || Observations | 145051 | 145051 | 145051 | 145051 | 145051 | 145051 | 134018 | 145051 |
| Marginal R2 / Conditional R2 | 0.000 / 0.493 | 0.045 / 0.508 | 0.091 / 0.562 | 0.128 / 0.573 | 0.136 / 0.576 | 0.136 / 0.576 | 0.426 / 0.641 | 0.129 / 0.573 |
