# Supplementary material for: The ADHD deficit in school performance across sex and parental education: A prospective sibling‐comparison register study of 344,152 Norwegian adolescents
Source: JCPP Adv. 2022 Feb 12;2(1):e12064. doi: 10.1002/jcv2.12064 (PMC10242882; doi:10.1002/jcv2.12064)
Supplement: Supplementary file 1 — Supplementary Material S1 [file JCV2-2-e12064-s001.zip › Supporting Information/Supplementary Tables/Table S6.html]

Table S6: Regression Table – Individual School Subjects (Sibling Models)

| Dependent Variable: Grades (z-score) | Norwegian (Primary) | Norwegian (Oral) | Norwegian (Secondary) | English (Written) | English (Oral) | Mathematics | Science | Social Studies | Religion | Sports | Food and Health | Music | Arts and Crafts |
| Predictors | Estimates (95% CIs) | Estimates (95% CIs) | Estimates (95% CIs) | Estimates (95% CIs) | Estimates (95% CIs) | Estimates (95% CIs) | Estimates (95% CIs) | Estimates (95% CIs) | Estimates (95% CIs) | Estimates (95% CIs) | Estimates (95% CIs) | Estimates (95% CIs) | Estimates (95% CIs) |
| ADHD (P81) Within Families | -0.51 (-0.54 – -0.48) | -0.51 (-0.54 – -0.48) | -0.42 (-0.46 – -0.39) | -0.54 (-0.57 – -0.51) | -0.49 (-0.52 – -0.46) | -0.58 (-0.61 – -0.54) | -0.58 (-0.61 – -0.54) | -0.57 (-0.60 – -0.53) | -0.54 (-0.58 – -0.51) | -0.39 (-0.42 – -0.36) | -0.39 (-0.41 – -0.36) | -0.40 (-0.43 – -0.37) | -0.33 (-0.36 – -0.31) |
| ADHD (P81) Between Families | -0.51 (-0.56 – -0.46) | -0.53 (-0.59 – -0.48) | -0.58 (-0.63 – -0.52) | -0.48 (-0.53 – -0.42) | -0.41 (-0.47 – -0.36) | -0.69 (-0.76 – -0.63) | -0.59 (-0.65 – -0.53) | -0.63 (-0.68 – -0.57) | -0.65 (-0.70 – -0.59) | -0.47 (-0.52 – -0.43) | -0.38 (-0.42 – -0.34) | -0.44 (-0.49 – -0.39) | -0.29 (-0.33 – -0.24) |
| Sex: Boys | *Reference* | *Reference* | *Reference* | *Reference* | *Reference* | *Reference* | *Reference* | *Reference* | *Reference* | *Reference* | *Reference* | *Reference* | *Reference* |
| Sex: Girls | 0.60 (0.59 – 0.61) | 0.50 (0.49 – 0.51) | 0.58 (0.58 – 0.59) | 0.39 (0.38 – 0.40) | 0.30 (0.29 – 0.31) | 0.18 (0.17 – 0.19) | 0.34 (0.33 – 0.35) | 0.33 (0.32 – 0.34) | 0.54 (0.53 – 0.55) | -0.16 (-0.17 – -0.15) | 0.55 (0.54 – 0.55) | 0.41 (0.40 – 0.42) | 0.56 (0.55 – 0.56) |
| Birth Year: 1997 | *Reference* | *Reference* | *Reference* | *Reference* | *Reference* | *Reference* | *Reference* | *Reference* | *Reference* | *Reference* | *Reference* | *Reference* | *Reference* |
| Birth Year: 1998 | 0.01 (-0.01 – 0.02) | 0.05 (0.03 – 0.06) | 0.01 (-0.00 – 0.03) | 0.06 (0.04 – 0.07) | 0.06 (0.05 – 0.08) | 0.03 (0.02 – 0.05) | 0.05 (0.04 – 0.07) | 0.05 (0.03 – 0.06) | 0.06 (0.04 – 0.07) | 0.06 (0.05 – 0.07) | 0.03 (0.02 – 0.04) | 0.04 (0.03 – 0.06) | 0.04 (0.03 – 0.05) |
| Birth Year: 1999 | -0.01 (-0.03 – 0.00) | 0.07 (0.06 – 0.09) | -0.02 (-0.03 – -0.00) | 0.03 (0.02 – 0.05) | 0.08 (0.07 – 0.10) | -0.03 (-0.05 – -0.02) | 0.08 (0.06 – 0.09) | 0.08 (0.06 – 0.09) | 0.08 (0.07 – 0.10) | 0.10 (0.09 – 0.11) | 0.05 (0.04 – 0.07) | 0.08 (0.07 – 0.10) | 0.05 (0.04 – 0.06) |
| Birth Year: 2000 | 0.02 (0.00 – 0.03) | 0.12 (0.11 – 0.14) | 0.01 (-0.01 – 0.02) | 0.08 (0.06 – 0.10) | 0.12 (0.11 – 0.14) | 0.06 (0.04 – 0.07) | 0.12 (0.11 – 0.14) | 0.10 (0.09 – 0.12) | 0.12 (0.10 – 0.14) | 0.13 (0.12 – 0.15) | 0.10 (0.09 – 0.11) | 0.11 (0.09 – 0.12) | 0.08 (0.07 – 0.09) |
| Birth Year: 2001 | 0.04 (0.02 – 0.05) | 0.14 (0.12 – 0.16) | 0.05 (0.03 – 0.06) | 0.08 (0.07 – 0.10) | 0.16 (0.14 – 0.17) | 0.09 (0.07 – 0.11) | 0.13 (0.11 – 0.15) | 0.13 (0.12 – 0.15) | 0.15 (0.13 – 0.17) | 0.19 (0.17 – 0.20) | 0.11 (0.09 – 0.12) | 0.13 (0.11 – 0.14) | 0.10 (0.09 – 0.11) |
| Birth Year: 2002 | 0.07 (0.05 – 0.09) | 0.16 (0.15 – 0.18) | 0.09 (0.07 – 0.11) | 0.14 (0.12 – 0.16) | 0.19 (0.17 – 0.21) | 0.18 (0.16 – 0.20) | 0.15 (0.13 – 0.17) | 0.17 (0.15 – 0.19) | 0.19 (0.17 – 0.20) | 0.21 (0.19 – 0.22) | 0.13 (0.12 – 0.15) | 0.16 (0.14 – 0.18) | 0.13 (0.11 – 0.15) |
| Birth Month: January | *Reference* | *Reference* | *Reference* | *Reference* | *Reference* | *Reference* | *Reference* | *Reference* | *Reference* | *Reference* | *Reference* | *Reference* | *Reference* |
| Birth Month: February | -0.04 (-0.06 – -0.02) | -0.02 (-0.04 – 0.00) | -0.02 (-0.04 – -0.00) | -0.02 (-0.05 – -0.00) | -0.01 (-0.03 – 0.02) | -0.02 (-0.04 – 0.01) | -0.03 (-0.05 – -0.01) | -0.04 (-0.06 – -0.02) | -0.02 (-0.04 – 0.00) | -0.00 (-0.02 – 0.02) | -0.01 (-0.03 – 0.00) | -0.01 (-0.03 – 0.00) | -0.03 (-0.04 – -0.01) |
| Birth Month: March | -0.05 (-0.07 – -0.03) | -0.03 (-0.05 – -0.01) | -0.04 (-0.06 – -0.01) | -0.04 (-0.07 – -0.02) | -0.02 (-0.04 – 0.01) | -0.02 (-0.05 – 0.00) | -0.03 (-0.06 – -0.01) | -0.05 (-0.07 – -0.03) | -0.04 (-0.06 – -0.02) | -0.02 (-0.03 – 0.00) | -0.02 (-0.04 – -0.01) | -0.01 (-0.03 – 0.01) | -0.03 (-0.04 – -0.01) |
| Birth Month: April | -0.03 (-0.05 – -0.01) | -0.03 (-0.05 – -0.01) | -0.01 (-0.04 – 0.01) | -0.02 (-0.04 – 0.00) | -0.00 (-0.02 – 0.02) | -0.02 (-0.05 – 0.00) | -0.01 (-0.04 – 0.01) | -0.02 (-0.05 – -0.00) | -0.02 (-0.04 – 0.01) | -0.02 (-0.03 – 0.00) | -0.02 (-0.04 – -0.00) | -0.01 (-0.03 – 0.01) | -0.02 (-0.04 – -0.00) |
| Birth Month: May | -0.08 (-0.10 – -0.06) | -0.06 (-0.08 – -0.04) | -0.06 (-0.08 – -0.04) | -0.06 (-0.08 – -0.04) | -0.03 (-0.05 – -0.01) | -0.04 (-0.07 – -0.02) | -0.05 (-0.08 – -0.03) | -0.06 (-0.08 – -0.04) | -0.05 (-0.07 – -0.03) | -0.05 (-0.07 – -0.03) | -0.05 (-0.06 – -0.03) | -0.03 (-0.05 – -0.01) | -0.05 (-0.07 – -0.03) |
| Birth Month: June | -0.08 (-0.10 – -0.06) | -0.06 (-0.08 – -0.03) | -0.06 (-0.08 – -0.04) | -0.07 (-0.09 – -0.05) | -0.04 (-0.07 – -0.02) | -0.06 (-0.09 – -0.04) | -0.06 (-0.09 – -0.04) | -0.08 (-0.10 – -0.05) | -0.06 (-0.08 – -0.04) | -0.06 (-0.08 – -0.04) | -0.06 (-0.08 – -0.04) | -0.04 (-0.06 – -0.02) | -0.06 (-0.08 – -0.04) |
| Birth Month: July | -0.10 (-0.12 – -0.08) | -0.08 (-0.10 – -0.06) | -0.08 (-0.10 – -0.06) | -0.09 (-0.11 – -0.07) | -0.05 (-0.07 – -0.03) | -0.10 (-0.12 – -0.07) | -0.11 (-0.13 – -0.08) | -0.09 (-0.11 – -0.07) | -0.09 (-0.11 – -0.06) | -0.08 (-0.09 – -0.06) | -0.07 (-0.09 – -0.06) | -0.05 (-0.07 – -0.03) | -0.07 (-0.09 – -0.05) |
| Birth Month: August | -0.11 (-0.14 – -0.09) | -0.08 (-0.10 – -0.06) | -0.09 (-0.11 – -0.07) | -0.09 (-0.12 – -0.07) | -0.06 (-0.08 – -0.04) | -0.08 (-0.11 – -0.06) | -0.09 (-0.11 – -0.07) | -0.09 (-0.12 – -0.07) | -0.08 (-0.10 – -0.06) | -0.08 (-0.10 – -0.06) | -0.09 (-0.10 – -0.07) | -0.05 (-0.07 – -0.04) | -0.08 (-0.10 – -0.06) |
| Birth Month: September | -0.14 (-0.16 – -0.12) | -0.10 (-0.12 – -0.08) | -0.11 (-0.13 – -0.09) | -0.11 (-0.13 – -0.09) | -0.08 (-0.10 – -0.06) | -0.11 (-0.14 – -0.09) | -0.11 (-0.13 – -0.09) | -0.12 (-0.14 – -0.10) | -0.10 (-0.13 – -0.08) | -0.10 (-0.12 – -0.09) | -0.11 (-0.12 – -0.09) | -0.07 (-0.09 – -0.05) | -0.09 (-0.11 – -0.07) |
| Birth Month: October | -0.15 (-0.17 – -0.13) | -0.12 (-0.14 – -0.10) | -0.12 (-0.14 – -0.10) | -0.13 (-0.15 – -0.11) | -0.08 (-0.11 – -0.06) | -0.12 (-0.15 – -0.10) | -0.12 (-0.14 – -0.10) | -0.14 (-0.16 – -0.11) | -0.11 (-0.13 – -0.09) | -0.13 (-0.14 – -0.11) | -0.11 (-0.13 – -0.09) | -0.07 (-0.09 – -0.05) | -0.10 (-0.12 – -0.08) |
| Birth Month: November | -0.17 (-0.19 – -0.15) | -0.13 (-0.15 – -0.11) | -0.14 (-0.16 – -0.12) | -0.15 (-0.18 – -0.13) | -0.10 (-0.12 – -0.07) | -0.15 (-0.18 – -0.13) | -0.15 (-0.17 – -0.12) | -0.15 (-0.18 – -0.13) | -0.14 (-0.16 – -0.11) | -0.14 (-0.16 – -0.12) | -0.13 (-0.15 – -0.11) | -0.09 (-0.11 – -0.07) | -0.11 (-0.13 – -0.09) |
| Birth Month: December | -0.19 (-0.21 – -0.17) | -0.16 (-0.18 – -0.13) | -0.16 (-0.18 – -0.14) | -0.16 (-0.18 – -0.14) | -0.10 (-0.13 – -0.08) | -0.15 (-0.17 – -0.12) | -0.15 (-0.17 – -0.13) | -0.17 (-0.19 – -0.14) | -0.15 (-0.18 – -0.13) | -0.14 (-0.16 – -0.12) | -0.14 (-0.16 – -0.12) | -0.10 (-0.12 – -0.08) | -0.12 (-0.14 – -0.10) |
| Parity: First-Born | *Reference* | *Reference* | *Reference* | *Reference* | *Reference* | *Reference* | *Reference* | *Reference* | *Reference* | *Reference* | *Reference* | *Reference* | *Reference* |
| Parity: Second-Born | -0.15 (-0.16 – -0.14) | -0.15 (-0.16 – -0.14) | -0.14 (-0.15 – -0.13) | -0.12 (-0.13 – -0.11) | -0.12 (-0.13 – -0.11) | -0.18 (-0.19 – -0.16) | -0.18 (-0.19 – -0.17) | -0.17 (-0.18 – -0.16) | -0.17 (-0.18 – -0.15) | -0.03 (-0.04 – -0.02) | -0.06 (-0.07 – -0.05) | -0.08 (-0.09 – -0.07) | -0.07 (-0.08 – -0.06) |
| Parity: Third-Born | -0.26 (-0.27 – -0.24) | -0.24 (-0.26 – -0.23) | -0.24 (-0.25 – -0.22) | -0.21 (-0.23 – -0.19) | -0.20 (-0.21 – -0.18) | -0.32 (-0.34 – -0.30) | -0.30 (-0.32 – -0.28) | -0.28 (-0.30 – -0.26) | -0.28 (-0.30 – -0.26) | -0.09 (-0.11 – -0.08) | -0.14 (-0.15 – -0.12) | -0.16 (-0.17 – -0.14) | -0.14 (-0.16 – -0.13) |
| Parity: Fourth-Born | -0.36 (-0.39 – -0.34) | -0.37 (-0.40 – -0.35) | -0.32 (-0.34 – -0.30) | -0.29 (-0.32 – -0.27) | -0.28 (-0.30 – -0.25) | -0.47 (-0.50 – -0.44) | -0.44 (-0.47 – -0.41) | -0.42 (-0.44 – -0.39) | -0.39 (-0.42 – -0.36) | -0.17 (-0.19 – -0.14) | -0.23 (-0.25 – -0.21) | -0.24 (-0.26 – -0.21) | -0.21 (-0.23 – -0.19) |
| Parity: Fifth-Born or later | -0.50 (-0.53 – -0.47) | -0.48 (-0.52 – -0.45) | -0.44 (-0.47 – -0.40) | -0.39 (-0.43 – -0.35) | -0.38 (-0.42 – -0.35) | -0.61 (-0.65 – -0.56) | -0.57 (-0.61 – -0.54) | -0.51 (-0.55 – -0.48) | -0.48 (-0.52 – -0.44) | -0.26 (-0.29 – -0.23) | -0.31 (-0.33 – -0.28) | -0.35 (-0.38 – -0.32) | -0.31 (-0.34 – -0.28) |
| (Intercept) | -0.27 (-0.29 – -0.25) | 0.04 (0.02 – 0.06) | -0.50 (-0.52 – -0.48) | -0.19 (-0.21 – -0.17) | 0.03 (0.01 – 0.05) | -0.30 (-0.32 – -0.27) | 0.08 (0.06 – 0.10) | 0.20 (0.18 – 0.22) | 0.04 (0.02 – 0.06) | 0.55 (0.53 – 0.56) | 0.21 (0.20 – 0.23) | 0.16 (0.14 – 0.18) | 0.09 (0.07 – 0.10) |
| Random Effects | | | | | | | | | | | | | |
| σ2 | 0.45 | 0.49 | 0.45 | 0.54 | 0.52 | 0.65 | 0.60 | 0.56 | 0.56 | 0.40 | 0.34 | 0.43 | 0.42 || τ00 | 0.29 parents | 0.30 parents | 0.27 parents | 0.36 parents | 0.33 parents | 0.57 parents | 0.41 parents | 0.37 parents | 0.36 parents | 0.25 parents | 0.19 parents | 0.25 parents | 0.18 parents || ICC | 0.40 | 0.38 | 0.37 | 0.40 | 0.39 | 0.47 | 0.41 | 0.40 | 0.39 | 0.39 | 0.35 | 0.36 | 0.30 || N | 69369 parents | 69372 parents | 67243 parents | 69400 parents | 69424 parents | 69429 parents | 69273 parents | 69286 parents | 68406 parents | 69431 parents | 69237 parents | 69245 parents | 69224 parents || Observations | 142935 | 142921 | 131234 | 142532 | 142760 | 142771 | 143031 | 143141 | 140065 | 142525 | 142590 | 142478 | 142844 |
| Marginal R2 / Conditional R2 | 0.154 / 0.489 | 0.118 / 0.455 | 0.141 / 0.461 | 0.077 / 0.446 | 0.059 / 0.425 | 0.053 / 0.494 | 0.076 / 0.451 | 0.079 / 0.447 | 0.119 / 0.460 | 0.044 / 0.418 | 0.159 / 0.455 | 0.091 / 0.422 | 0.139 / 0.399 |
