# Supplementary material for: The ADHD deficit in school performance across sex and parental education: A prospective sibling‐comparison register study of 344,152 Norwegian adolescents
Source: JCPP Adv. 2022 Feb 12;2(1):e12064. doi: 10.1002/jcv2.12064 (PMC10242882; doi:10.1002/jcv2.12064)
Supplement: Supplementary file 1 — Supplementary Material S1 [file JCV2-2-e12064-s001.zip › Supporting Information/Supplementary Tables/Table S8.html]

Table S8: Regression Table – Individual School Subjects and Interaction with Parental Education (Fully Adjusted Models)

| Dependent Variable: Grades (z-score) | Norwegian (Primary) | Norwegian (Oral) | Norwegian (Secondary) | English (Written) | English (Oral) | Mathematics | Science | Social Studies | Religion | Sports | Food and Health | Music | Arts and Crafts |
| Predictors | Estimates (95% CIs) | Estimates (95% CIs) | Estimates (95% CIs) | Estimates (95% CIs) | Estimates (95% CIs) | Estimates (95% CIs) | Estimates (95% CIs) | Estimates (95% CIs) | Estimates (95% CIs) | Estimates (95% CIs) | Estimates (95% CIs) | Estimates (95% CIs) | Estimates (95% CIs) |
| ADHD (P81) | -0.54 (-0.57 – -0.50) | -0.60 (-0.64 – -0.56) | -0.49 (-0.53 – -0.44) | -0.60 (-0.64 – -0.56) | -0.52 (-0.56 – -0.48) | -0.60 (-0.65 – -0.56) | -0.66 (-0.70 – -0.61) | -0.67 (-0.71 – -0.63) | -0.70 (-0.74 – -0.65) | -0.52 (-0.56 – -0.49) | -0.46 (-0.50 – -0.43) | -0.49 (-0.52 – -0.45) | -0.41 (-0.44 – -0.37) |
| ADHD \* Parental Ed: High School *(Interaction)* | -0.15 (-0.19 – -0.11) | -0.11 (-0.15 – -0.07) | -0.13 (-0.18 – -0.07) | -0.11 (-0.16 – -0.06) | -0.10 (-0.15 – -0.05) | -0.19 (-0.24 – -0.13) | -0.13 (-0.18 – -0.08) | -0.12 (-0.17 – -0.07) | -0.11 (-0.16 – -0.06) | -0.08 (-0.12 – -0.04) | -0.09 (-0.12 – -0.05) | -0.08 (-0.13 – -0.04) | -0.07 (-0.11 – -0.03) |
| ADHD \* Parental Ed: Bachelor *(Interaction)* | -0.23 (-0.28 – -0.19) | -0.20 (-0.25 – -0.16) | -0.21 (-0.26 – -0.15) | -0.15 (-0.21 – -0.10) | -0.16 (-0.21 – -0.11) | -0.35 (-0.41 – -0.29) | -0.24 (-0.29 – -0.19) | -0.21 (-0.26 – -0.16) | -0.18 (-0.24 – -0.13) | -0.12 (-0.17 – -0.08) | -0.13 (-0.17 – -0.10) | -0.12 (-0.17 – -0.08) | -0.07 (-0.11 – -0.03) |
| ADHD \* Parental Ed: Master*(Interaction)* | -0.25 (-0.31 – -0.18) | -0.17 (-0.24 – -0.10) | -0.20 (-0.27 – -0.12) | -0.14 (-0.21 – -0.07) | -0.13 (-0.20 – -0.06) | -0.40 (-0.48 – -0.32) | -0.24 (-0.31 – -0.17) | -0.16 (-0.23 – -0.09) | -0.13 (-0.20 – -0.06) | -0.12 (-0.18 – -0.05) | -0.17 (-0.22 – -0.11) | -0.11 (-0.18 – -0.05) | -0.08 (-0.14 – -0.02) |
| ADHD \* Parental Ed: Missing *(Interaction)* | 0.04 (-0.04 – 0.12) | -0.02 (-0.10 – 0.07) | -0.06 (-0.16 – 0.05) | 0.02 (-0.07 – 0.12) | 0.03 (-0.06 – 0.12) | -0.00 (-0.11 – 0.11) | 0.04 (-0.06 – 0.13) | -0.01 (-0.10 – 0.08) | -0.03 (-0.12 – 0.07) | 0.04 (-0.04 – 0.12) | 0.03 (-0.04 – 0.10) | 0.06 (-0.02 – 0.14) | -0.07 (-0.15 – 0.00) |
| Parental Education: No High School | *Reference* | *Reference* | *Reference* | *Reference* | *Reference* | *Reference* | *Reference* | *Reference* | *Reference* | *Reference* | *Reference* | *Reference* | *Reference* |
| Parental Education: High School | 0.26 (0.25 – 0.27) | 0.29 (0.27 – 0.30) | 0.24 (0.23 – 0.25) | 0.23 (0.22 – 0.24) | 0.23 (0.22 – 0.24) | 0.38 (0.36 – 0.39) | 0.35 (0.34 – 0.36) | 0.32 (0.31 – 0.33) | 0.29 (0.28 – 0.30) | 0.28 (0.27 – 0.29) | 0.23 (0.22 – 0.24) | 0.25 (0.24 – 0.26) | 0.22 (0.21 – 0.23) |
| Parental Education: Bachelor's Degree (or equiv) | 0.65 (0.64 – 0.66) | 0.69 (0.68 – 0.70) | 0.60 (0.59 – 0.61) | 0.63 (0.61 – 0.64) | 0.62 (0.61 – 0.63) | 0.89 (0.87 – 0.90) | 0.79 (0.78 – 0.80) | 0.77 (0.76 – 0.78) | 0.73 (0.72 – 0.74) | 0.54 (0.53 – 0.55) | 0.47 (0.46 – 0.48) | 0.55 (0.54 – 0.56) | 0.43 (0.42 – 0.44) |
| Parental Education: Master's Degree (or equiv) | 0.96 (0.94 – 0.97) | 1.00 (0.99 – 1.01) | 0.88 (0.87 – 0.90) | 0.98 (0.96 – 0.99) | 0.95 (0.94 – 0.96) | 1.35 (1.33 – 1.36) | 1.15 (1.14 – 1.17) | 1.11 (1.10 – 1.12) | 1.06 (1.05 – 1.07) | 0.68 (0.67 – 0.69) | 0.62 (0.61 – 0.63) | 0.78 (0.77 – 0.79) | 0.59 (0.58 – 0.60) |
| Parental Education: Missing | 0.06 (0.04 – 0.07) | 0.14 (0.12 – 0.15) | 0.11 (0.09 – 0.13) | 0.20 (0.18 – 0.22) | 0.24 (0.22 – 0.26) | 0.14 (0.12 – 0.16) | 0.15 (0.13 – 0.17) | 0.18 (0.16 – 0.20) | 0.20 (0.18 – 0.22) | 0.13 (0.11 – 0.14) | 0.03 (0.02 – 0.05) | 0.09 (0.07 – 0.11) | 0.06 (0.05 – 0.08) |
| Sex: Boys | *Reference* | *Reference* | *Reference* | *Reference* | *Reference* | *Reference* | *Reference* | *Reference* | *Reference* | *Reference* | *Reference* | *Reference* | *Reference* |
| Sex: Girls | 0.61 (0.60 – 0.61) | 0.51 (0.50 – 0.51) | 0.58 (0.58 – 0.59) | 0.39 (0.38 – 0.39) | 0.30 (0.29 – 0.30) | 0.18 (0.17 – 0.19) | 0.35 (0.34 – 0.35) | 0.33 (0.32 – 0.34) | 0.54 (0.53 – 0.54) | -0.16 (-0.17 – -0.16) | 0.55 (0.55 – 0.56) | 0.42 (0.41 – 0.43) | 0.57 (0.56 – 0.57) |
| Birth Year: 1997 | *Reference* | *Reference* | *Reference* | *Reference* | *Reference* | *Reference* | *Reference* | *Reference* | *Reference* | *Reference* | *Reference* | *Reference* | *Reference* |
| Birth Year: 1998 | -0.01 (-0.02 – 0.00) | 0.03 (0.02 – 0.04) | -0.00 (-0.01 – 0.01) | 0.03 (0.02 – 0.04) | 0.05 (0.04 – 0.06) | 0.02 (0.01 – 0.03) | 0.04 (0.02 – 0.05) | 0.04 (0.02 – 0.05) | 0.05 (0.04 – 0.06) | 0.04 (0.03 – 0.05) | 0.02 (0.01 – 0.03) | 0.03 (0.02 – 0.04) | 0.03 (0.02 – 0.04) |
| Birth Year: 1999 | -0.04 (-0.05 – -0.03) | 0.04 (0.03 – 0.05) | -0.05 (-0.05 – -0.04) | -0.01 (-0.02 – 0.00) | 0.04 (0.03 – 0.05) | -0.06 (-0.07 – -0.05) | 0.05 (0.04 – 0.06) | 0.06 (0.05 – 0.07) | 0.06 (0.05 – 0.07) | 0.08 (0.07 – 0.08) | 0.04 (0.03 – 0.05) | 0.06 (0.05 – 0.07) | 0.04 (0.03 – 0.05) |
| Birth Year: 2000 | -0.03 (-0.04 – -0.02) | 0.06 (0.05 – 0.07) | -0.03 (-0.04 – -0.02) | 0.00 (-0.01 – 0.02) | 0.05 (0.04 – 0.06) | -0.00 (-0.01 – 0.01) | 0.07 (0.06 – 0.08) | 0.06 (0.05 – 0.07) | 0.08 (0.07 – 0.09) | 0.09 (0.08 – 0.10) | 0.07 (0.07 – 0.08) | 0.06 (0.05 – 0.07) | 0.06 (0.05 – 0.07) |
| Birth Year: 2001 | -0.04 (-0.05 – -0.03) | 0.06 (0.05 – 0.07) | -0.01 (-0.02 – -0.00) | -0.01 (-0.02 – -0.00) | 0.06 (0.05 – 0.07) | 0.02 (0.01 – 0.04) | 0.07 (0.06 – 0.08) | 0.07 (0.06 – 0.08) | 0.08 (0.07 – 0.09) | 0.14 (0.13 – 0.14) | 0.07 (0.06 – 0.08) | 0.07 (0.06 – 0.08) | 0.07 (0.06 – 0.08) |
| Birth Year: 2002 | -0.02 (-0.02 – -0.01) | 0.07 (0.06 – 0.08) | 0.03 (0.02 – 0.04) | 0.03 (0.02 – 0.04) | 0.08 (0.07 – 0.09) | 0.09 (0.08 – 0.10) | 0.08 (0.06 – 0.09) | 0.09 (0.08 – 0.11) | 0.11 (0.10 – 0.12) | 0.16 (0.15 – 0.16) | 0.09 (0.08 – 0.10) | 0.09 (0.08 – 0.10) | 0.09 (0.09 – 0.10) |
| Birth Month: January | *Reference* | *Reference* | *Reference* | *Reference* | *Reference* | *Reference* | *Reference* | *Reference* | *Reference* | *Reference* | *Reference* | *Reference* | *Reference* |
| Birth Month: February | -0.04 (-0.05 – -0.02) | -0.02 (-0.03 – -0.00) | -0.02 (-0.03 – -0.01) | -0.03 (-0.05 – -0.01) | -0.02 (-0.04 – -0.01) | -0.02 (-0.04 – -0.01) | -0.02 (-0.04 – -0.00) | -0.03 (-0.04 – -0.01) | -0.02 (-0.04 – -0.00) | 0.01 (-0.01 – 0.02) | -0.01 (-0.02 – 0.00) | -0.01 (-0.02 – 0.01) | -0.01 (-0.02 – 0.01) |
| Birth Month: March | -0.05 (-0.06 – -0.03) | -0.04 (-0.05 – -0.02) | -0.04 (-0.05 – -0.02) | -0.05 (-0.07 – -0.04) | -0.03 (-0.05 – -0.02) | -0.03 (-0.05 – -0.01) | -0.04 (-0.05 – -0.02) | -0.05 (-0.06 – -0.03) | -0.04 (-0.05 – -0.02) | -0.01 (-0.02 – 0.00) | -0.02 (-0.04 – -0.01) | -0.01 (-0.03 – -0.00) | -0.02 (-0.03 – -0.01) |
| Birth Month: April | -0.04 (-0.06 – -0.03) | -0.04 (-0.06 – -0.03) | -0.02 (-0.04 – -0.01) | -0.04 (-0.05 – -0.02) | -0.02 (-0.04 – -0.01) | -0.03 (-0.05 – -0.01) | -0.02 (-0.04 – -0.01) | -0.04 (-0.05 – -0.02) | -0.03 (-0.05 – -0.02) | -0.02 (-0.03 – -0.00) | -0.02 (-0.04 – -0.01) | -0.02 (-0.03 – -0.00) | -0.02 (-0.03 – -0.01) |
| Birth Month: May | -0.08 (-0.09 – -0.07) | -0.06 (-0.07 – -0.05) | -0.06 (-0.07 – -0.05) | -0.07 (-0.08 – -0.05) | -0.04 (-0.06 – -0.03) | -0.05 (-0.07 – -0.03) | -0.05 (-0.07 – -0.04) | -0.06 (-0.08 – -0.05) | -0.06 (-0.07 – -0.04) | -0.04 (-0.05 – -0.03) | -0.05 (-0.06 – -0.03) | -0.03 (-0.04 – -0.02) | -0.03 (-0.05 – -0.02) |
| Birth Month: June | -0.08 (-0.10 – -0.07) | -0.06 (-0.08 – -0.05) | -0.07 (-0.08 – -0.05) | -0.08 (-0.09 – -0.06) | -0.06 (-0.08 – -0.05) | -0.07 (-0.09 – -0.05) | -0.07 (-0.08 – -0.05) | -0.07 (-0.08 – -0.05) | -0.06 (-0.08 – -0.04) | -0.05 (-0.06 – -0.04) | -0.06 (-0.07 – -0.05) | -0.04 (-0.06 – -0.03) | -0.05 (-0.06 – -0.03) |
| Birth Month: July | -0.11 (-0.12 – -0.09) | -0.09 (-0.11 – -0.08) | -0.09 (-0.10 – -0.07) | -0.09 (-0.11 – -0.08) | -0.07 (-0.08 – -0.06) | -0.10 (-0.11 – -0.08) | -0.10 (-0.12 – -0.09) | -0.10 (-0.11 – -0.08) | -0.09 (-0.11 – -0.08) | -0.07 (-0.08 – -0.06) | -0.08 (-0.09 – -0.07) | -0.06 (-0.07 – -0.05) | -0.07 (-0.08 – -0.06) |
| Birth Month: August | -0.13 (-0.14 – -0.11) | -0.10 (-0.12 – -0.09) | -0.10 (-0.12 – -0.09) | -0.11 (-0.13 – -0.10) | -0.08 (-0.09 – -0.06) | -0.10 (-0.12 – -0.09) | -0.11 (-0.12 – -0.09) | -0.11 (-0.13 – -0.10) | -0.10 (-0.11 – -0.08) | -0.08 (-0.10 – -0.07) | -0.09 (-0.10 – -0.08) | -0.07 (-0.08 – -0.05) | -0.07 (-0.09 – -0.06) |
| Birth Month: September | -0.15 (-0.16 – -0.13) | -0.12 (-0.14 – -0.11) | -0.12 (-0.13 – -0.10) | -0.13 (-0.15 – -0.12) | -0.10 (-0.12 – -0.09) | -0.12 (-0.14 – -0.11) | -0.12 (-0.13 – -0.10) | -0.13 (-0.14 – -0.11) | -0.12 (-0.13 – -0.10) | -0.10 (-0.12 – -0.09) | -0.11 (-0.12 – -0.10) | -0.08 (-0.09 – -0.07) | -0.09 (-0.10 – -0.08) |
| Birth Month: October | -0.16 (-0.18 – -0.15) | -0.13 (-0.15 – -0.12) | -0.13 (-0.15 – -0.12) | -0.14 (-0.16 – -0.13) | -0.11 (-0.13 – -0.10) | -0.14 (-0.15 – -0.12) | -0.13 (-0.15 – -0.11) | -0.14 (-0.16 – -0.13) | -0.13 (-0.14 – -0.11) | -0.12 (-0.13 – -0.10) | -0.12 (-0.13 – -0.11) | -0.09 (-0.11 – -0.08) | -0.10 (-0.12 – -0.09) |
| Birth Month: November | -0.18 (-0.19 – -0.17) | -0.15 (-0.17 – -0.14) | -0.15 (-0.16 – -0.13) | -0.16 (-0.17 – -0.14) | -0.12 (-0.14 – -0.11) | -0.16 (-0.18 – -0.14) | -0.15 (-0.17 – -0.13) | -0.16 (-0.18 – -0.15) | -0.15 (-0.16 – -0.13) | -0.14 (-0.16 – -0.13) | -0.13 (-0.15 – -0.12) | -0.10 (-0.11 – -0.08) | -0.11 (-0.12 – -0.10) |
| Birth Month: December | -0.20 (-0.21 – -0.19) | -0.17 (-0.19 – -0.16) | -0.17 (-0.19 – -0.16) | -0.18 (-0.20 – -0.17) | -0.14 (-0.15 – -0.12) | -0.17 (-0.19 – -0.16) | -0.16 (-0.18 – -0.15) | -0.18 (-0.20 – -0.17) | -0.17 (-0.18 – -0.15) | -0.15 (-0.16 – -0.14) | -0.14 (-0.15 – -0.13) | -0.11 (-0.13 – -0.10) | -0.12 (-0.13 – -0.10) |
| Parity: First-Born | *Reference* | *Reference* | *Reference* | *Reference* | *Reference* | *Reference* | *Reference* | *Reference* | *Reference* | *Reference* | *Reference* | *Reference* | *Reference* |
| Parity: Second-Born | -0.09 (-0.09 – -0.08) | -0.07 (-0.08 – -0.07) | -0.08 (-0.08 – -0.07) | -0.05 (-0.06 – -0.05) | -0.05 (-0.05 – -0.04) | -0.09 (-0.09 – -0.08) | -0.09 (-0.10 – -0.09) | -0.09 (-0.10 – -0.08) | -0.08 (-0.09 – -0.08) | 0.02 (0.02 – 0.03) | -0.02 (-0.03 – -0.01) | -0.03 (-0.04 – -0.02) | -0.03 (-0.04 – -0.02) |
| Parity: Third-Born | -0.12 (-0.13 – -0.11) | -0.10 (-0.11 – -0.09) | -0.10 (-0.11 – -0.09) | -0.07 (-0.08 – -0.06) | -0.06 (-0.07 – -0.05) | -0.14 (-0.15 – -0.13) | -0.14 (-0.15 – -0.13) | -0.13 (-0.13 – -0.12) | -0.13 (-0.14 – -0.12) | -0.01 (-0.01 – 0.00) | -0.06 (-0.06 – -0.05) | -0.05 (-0.06 – -0.04) | -0.06 (-0.07 – -0.05) |
| Parity: Fourth-Born | -0.17 (-0.19 – -0.16) | -0.17 (-0.19 – -0.16) | -0.13 (-0.14 – -0.11) | -0.11 (-0.13 – -0.09) | -0.11 (-0.12 – -0.09) | -0.21 (-0.23 – -0.19) | -0.21 (-0.23 – -0.20) | -0.20 (-0.22 – -0.19) | -0.19 (-0.20 – -0.17) | -0.07 (-0.09 – -0.06) | -0.12 (-0.13 – -0.10) | -0.10 (-0.11 – -0.09) | -0.10 (-0.11 – -0.09) |
| Parity: Fifth-Born or later | -0.24 (-0.27 – -0.22) | -0.21 (-0.23 – -0.19) | -0.18 (-0.21 – -0.16) | -0.16 (-0.19 – -0.14) | -0.15 (-0.17 – -0.13) | -0.27 (-0.30 – -0.25) | -0.27 (-0.29 – -0.24) | -0.22 (-0.24 – -0.19) | -0.20 (-0.22 – -0.17) | -0.10 (-0.12 – -0.08) | -0.15 (-0.17 – -0.13) | -0.16 (-0.18 – -0.14) | -0.16 (-0.18 – -0.14) |
| Parity: Missing | -0.18 (-0.28 – -0.09) | -0.13 (-0.23 – -0.04) | -0.13 (-0.24 – -0.01) | -0.19 (-0.30 – -0.09) | -0.19 (-0.29 – -0.09) | -0.13 (-0.25 – -0.01) | -0.15 (-0.26 – -0.04) | -0.10 (-0.21 – 0.00) | -0.11 (-0.21 – 0.00) | 0.07 (-0.02 – 0.17) | -0.10 (-0.18 – -0.02) | -0.05 (-0.14 – 0.04) | -0.02 (-0.11 – 0.07) |
| (Intercept) | -0.84 (-0.86 – -0.83) | -0.57 (-0.59 – -0.56) | -1.05 (-1.07 – -1.03) | -0.70 (-0.72 – -0.69) | -0.46 (-0.48 – -0.45) | -1.14 (-1.16 – -1.12) | -0.67 (-0.69 – -0.65) | -0.52 (-0.53 – -0.50) | -0.63 (-0.64 – -0.61) | 0.04 (0.02 – 0.05) | -0.24 (-0.25 – -0.22) | -0.33 (-0.35 – -0.32) | -0.34 (-0.36 – -0.33) |
| Observations | 338399 | 338327 | 309304 | 337405 | 338011 | 337741 | 338659 | 338995 | 332536 | 337088 | 337567 | 337156 | 338174 |
| R2 / R2 adjusted | 0.246 / 0.246 | 0.213 / 0.213 | 0.213 / 0.213 | 0.160 / 0.160 | 0.145 / 0.145 | 0.173 / 0.173 | 0.177 / 0.177 | 0.182 / 0.182 | 0.211 / 0.211 | 0.100 / 0.099 | 0.212 / 0.212 | 0.160 / 0.159 | 0.181 / 0.181 |
